# Supplementary material for: Selenium Supplementation and Prostate Health in a New Zealand Cohort
Source: Nutrients. 2019 Dec 18;12(1):2. doi: 10.3390/nu12010002 (PMC7019779; doi:10.3390/nu12010002)
Supplement: Supplementary file 1 [file nutrients-12-00002-s001.zip › nutrients-609717-supplementary document 1.pdf]

TREND Statement Checklist

Paper Section/ Topic	Item No	Descriptor	Reported? ✓	Pg #
**Title and Abstract**				
Title and Abstract	1	• Information on how unit were allocated to interventions	✓	1
		• Structured abstract recommended	✓	1
		• Information on target population or study sample	✓	1
**Introduction**				
Background	2	• Scientific background and explanation of rationale	✓	1-2
		• Theories used in designing behavioral interventions	✓	2
**Methods**				
Participants	3	• Eligibility criteria for participants, including criteria at different levels in recruitment/sampling plan (e.g., cities, clinics, subjects)	✓	2-3
		Method of recruitment (e.g., referral, self-selection), including the sampling method if a systematic sampling plan was implemented	✓	3
		• Recruitment setting	✓	3
		• Settings and locations where the data were collected	✓	3
Interventions	4	• Details of the interventions intended for each study condition and how and when they were actually administered, specifically including:		
		o Content: what was given?	✓	3
		o Delivery method: how was the content given?	✓	3
		o Unit of delivery: how were the subjects grouped during delivery?	✓	3
		o Deliverer: who delivered the intervention?	✓	3
		o Setting: where was the intervention delivered?	✓	3
		o Exposure quantity and duration: how many sessions or episodes or events were intended to be delivered? How long were they intended to last?	✓	3
		o Time span: how long was it intended to take to deliver the intervention to each unit?	✓	3
		o Activities to increase compliance or adherence (e.g., incentives)	✓	3-4
Objectives	5	• Specific objectives and hypotheses	✓	4
Outcomes	6	• Clearly defined primary and secondary outcome measures	✓	4
		• Methods used to collect data and any methods used to enhance the quality of measurements	✓	4-6
		• Information on validated instruments such as psychometric and biometric properties	✓	6
Sample Size	7	How sample size was determined and, when applicable, explanation of any interim analyses and stopping rules	✓	6
Assignment Method	8	Unit of assignment (the unit being assigned to study condition, e.g., individual, group, community)	✓	6-7
		Method used to assign units to study conditions, including details of any restriction (e.g., blocking, stratification, minimization)	✓	7
		Inclusion of aspects employed to help minimize potential bias induced due to non-randomization (e.g., matching)	✓	7

TREND Statement Checklist

Blinding (masking)	9	• Whether or not participants, those administering the interventions, and those assessing the outcomes were blinded to study condition assignment; if so, statement regarding how the blinding was accomplished and how it was assessed.	✓	7
Unit of Analysis	10	• Description of the smallest unit that is being analyzed to assess intervention effects (e.g., individual, group, or community)	✓	7
		• If the unit of analysis differs from the unit of assignment, the analytical method used to account for this (e.g., adjusting the standard error estimates by the design effect or using multilevel analysis)	✓	7
Statistical Methods	11	• Statistical methods used to compare study groups for primary methods outcome(s), including complex methods of correlated data	✓	8
		• Statistical methods used for additional analyses, such as a subgroup analyses and adjusted analysis	✓	8
		• Methods for imputing missing data, if used		
		• Statistical software or programs used	✓	8
**Results**				
Participant flow	12	Flow of participants through each stage of the study: enrollment, assignment, allocation, and intervention exposure, follow-up, analysis (a diagram is strongly recommended)	✓	9
		o Enrollment: the numbers of participants screened for eligibility, found to be eligible or not eligible, declined to be enrolled, and enrolled in the study	✓	10
		o Assignment: the numbers of participants assigned to a study condition	✓	10
		o Allocation and intervention exposure: the number of participants assigned to each study condition and the number of participants who received each intervention	✓	10
		o Follow-up: the number of participants who completed the follow-up or did not complete the follow-up (i.e., lost to follow-up), by study condition	✓	10
		o Analysis: the number of participants included in or excluded from the main analysis, by study condition	✓	10
		• Description of protocol deviations from study as planned, along with reasons	✓	10
Recruitment	13	• Dates defining the periods of recruitment and follow-up	✓	10
Baseline Data	14	Baseline demographic and clinical characteristics of participants in each study condition	✓	10-11
		Baseline characteristics for each study condition relevant to specific disease prevention research	✓	11
		Baseline comparisons of those lost to follow-up and those retained, overall and by study condition	✓	12
		• Comparison between study population at baseline and target population of interest	✓	13
Baseline equivalence	15	• Data on study group equivalence at baseline and statistical methods used to control for baseline differences	✓	13

TREND Statement Checklist

Numbers analyzed	16	• Number of participants (denominator) included in each analysis for each study condition, particularly when the denominators change for different outcomes; statement of the results in absolute numbers when feasible	✓	13
		• Indication of whether the analysis strategy was "intention to treat" or, if not, description of how non-compliers were treated in the analyses	✓	13
Outcomes and estimation	17	• For each primary and secondary outcome, a summary of results for each estimation study condition, and the estimated effect size and a confidence interval to indicate the precision	✓	14-22
		• Inclusion of null and negative findings	✓	23
		Inclusion of results from testing pre-specified causal pathways through which the intervention was intended to operate, if any	✓	23
Ancillary analyses	18	• Summary of other analyses performed, including subgroup or restricted analyses, indicating which are pre-specified or exploratory	✓	23
Adverse events	19	• Summary of all important adverse events or unintended effects in each study condition (including summary measures, effect size estimates, and confidence intervals)	✓	24-25
**DISCUSSION**				
Interpretation	20	• Interpretation of the results, taking into account study hypotheses, sources of potential bias, imprecision of measures, multiplicative analyses, and other limitations or weaknesses of the study	✓	25-26
		Discussion of results taking into account the mechanism by which the intervention was intended to work (causal pathways) or alternative mechanisms or explanations	✓	25-26
		Discussion of the success of and barriers to implementing the intervention, fidelity of implementation	✓	26
		• Discussion of research, programmatic, or policy implications	✓	26
Generalizability	21	• Generalizability (external validity) of the trial findings, taking into account the study population, the characteristics of the intervention, length of follow-up, incentives, compliance rates, specific sites/settings involved in the study, and other contextual issues	✓	27
Overall Evidence	22	• General interpretation of the results in the context of current evidence and current theory	✓	27

From: Des Jarlais, D. C., Lyles, C., Crepaz, N., & the Trend Group (2004). Improving the reporting quality of nonrandomized evaluations of behavioral and public health interventions: The TREND statement. *American Journal of Public Health*, 94, 361-366. For more information, visit: http://www.cdc.gov/trendstatement/

TREND Statement Checklist

1.Title- Optimising selenium intake for health- a pilot study

Abstract-

Introduction

New Zealand records the highest global incidence of age standardised prostate cancer incidence. Meanwhile, New Zealand soil is known for its low levels and hence low dietary selenium intakes. Since Prof Larry Clark and his group showed the benefits of 200µg/d selenium for a long duration of around 10 years was beneficial in reducing the risk of overall and many cancers including prostate cancer, this study was started as a biomarker based nutrigenomics study. This study wished to verify the impact of selenium supplementation on biomarkers known for a possibility of indirect associations with cancer aetiology.

Method

The target population was New Zealand men, in the age range 20-80y, residents of Auckland, with no-known cancers except for non-melanoma skin cancer, not taking any selenium supplements >50µg/d, willing to come to the study centre at the Faculty of Medical and Health Sciences, University of Auckland for evaluations, willing to give blood samples for analysis at 2-3 time points. Supplementation was within a short duration of six months which was the time required for plasma selenium stabilisation according to Prof. Larry Clark et al study. The primary outcome measures were DNA damage levels in leukocytes and activities of seleno antioxidant enzymes glutathione peroxidase and thioredoxin reductase in the red blood cells. We have additionally looked at the impact of supplemented selenium benefits for the prostate microarchitecture stability, using the secondary outcome prostate-specific antigen (PSA) as the surrogate marker. The biomarker variations were further stratified by demographic, lifestyle, diet and genetic factors to understand their variable impacts on selenium supplementation benefits.

Results

We have shown that the benefits vary by all the said aspects as reported in our published work. We see an inverse correlation between changes taking place in the serum levels of PSA and changes in the serum levels of selenium. Further data stratifications indicate that the benefits are best achieved in men carrying prostate cancer risk genotypes reported in our Auckland, New Zealand studies. This work is to be submitted for publication. We further wish to analyse supplementation-related metabolomics, proteomic and trace element changes, which can be compared against the prostate cancer associated metabolome, proteome and trace element profiles stratified by a GWAS scale genetic analysis.

Discussion

Supplementation benefits of 200µg/d selenium as selenized yeast measured through biomarkers indicate benefit variation with demographic, lifestyle, diet and genetic factors.

Introduction

2. Background-

Scientific background and explanation of rationale

The trace mineral selenium (Se), and its 25 known selenoproteins, are important in human health, with lower levels being associated with various health issues including cancer [1; 2; 3]. The benefits of Se supplementation towards prostate cancer (PC) prevention is however controversial (SELECT trial vs Se for Prostate Cancer Prevention Trial) [4; 5]. A recent systemic review and meta-analysis indicates the possible risk of diabetes associated with serum/plasma levels above 140ng/ml and the risk being higher in women compared to men [6]. The most recent Cochrane systematic review on Se for preventing cancer concludes that the risk ratio for PC incidence or mortality has no strong negative association with Se exposure [7]. These authors state that there may be other confounding factors in

understanding Se benefit variability for cancer prevention.

Se and biological functions- Influence of Se on various molecular mechanisms including that of DNA stability, DNA methylation, one-carbon metabolism, redox balance, histone acetylation and miRNA functioning has been recorded previously[2; 8; 9; 10; 11; 12; 13; 14]. Most molecular studies on Se benefits are either cell-line based or used animal models, with a few derived from human studies. Based on that, it is of high relevance to study the above impacts from human Se intervention studies. A recent review suggests that Se may increase risks of high-grade PC and type 2 diabetes [6; 7]. Another systemic review on Se concentration in blood and type-2 diabetes (DM2) risk records a summary odds ratio of 2.03 in observational studies, but not in randomised controlled studies [15] . A/Prof. Sinha and colleagues have shown that Se supplementation in US men have changed the plasma cellular proteome [16] that can have significant effects on health. Their study was based on a group of healthy men in US who have received 300µg/d selenized yeast for 48 weeks. Their assessments using isobaric tags for relative and absolute quantitation (iTRAQ) assays have shown differentially expressed proteins by Se supplementation including proteins involved in the complement and coagulation pathways, immune functions, lipid metabolism, and insulin resistance. Plasma Se levels at baseline in this group of men was 142±19ng/ml and supplementation has increased the levels to around 220ng/ml and 228±63ng/ml by weeks 24 and 48 respectively [17]. In comparison to this US cohort, the NZ cohort supplemented with 200µg/d Se as selenized yeast for 24 weeks increased the serum Se levels from a baseline of 112±24ng/ml to 160±25ng/ml [18]. It is interesting to know the impact on the proteome, when the serum Se levels were elevated to a lesser extent in the NZ cohort compared to the US cohort. According to Fairweather-Tait et al 2011, Se toxicity symptoms start when plasma/serum level reaches >250ng/ml [1]. The above makes us believe that adjusting serum Se levels to reach 120-150ng/ml (as estimated by us as the optimum level for DNA stability [10]) could provide beneficial effects on prostate integrity especially in NZ men who could be genetically prone to PC risk[19; 20]. Interestingly, our studies from New Zealand indicate that Se supplementation can reduce the levels of prostate-specific antigen (PSA), a well-known prostate glandular epithelial microarchitecture stability marker [21]. The inverse correlation between Se gain by supplementation and change is serum PSA is stronger only in men carrying risk genotypes for PC. This correlation was also stronger in men with alcohol consuming and never-smoking lifestyles with a BMI <26 (manuscript submitted for publication review). There is a possibility that adjustments of serum Se levels at least in these stratified groups can produce optimum benefits on prostate glandular epithelium health, leading to reduction of prostate disease including PC. However, before such practice can take place, it is important to understand detailed implications of Se supplementation on other cellular pathways that are important in health. However, it is worth investigating parallel molecular and nutritional interactions and impacts of adjusted serum Se levels before such recommendations can be derived for the prophylactic benefit of PC prone NZ men.

Theories used in designing behavioral interventions

The general New Zealand diet is low in Se and therefore a pre- and post- Se supplemented time points can be considered as low and medium dietary Se exposures points. Therefore, the current study considered the supplementation effect of 200µg/day of Se as selenized yeast among all of male subjects from this New Zealand cohort as a non-randomised study, and evaluated the outcomes associated with pre- and six month post-supplemented serum Se levels.

Methods

3. Participants

Eligibility criteria for participants, including criteria at different levels in recruitment/sampling plan (e.g., cities, clinics, subjects)-

Male subjects from Auckland, New Zealand, self-reported as having Caucasian (European) ancestry, within ages ranging from 20-80 years, with no history of cancers other than non-

melanoma skin cancers, and were not taking more than 50µg Selenium/day as supplements were eligible for the study.

Method of recruitment (e.g., referral, self-selection)
Recruitment was based on public advertisements placed in various formats, including area News Papers and National News Papers as well as circulation of study advert through the email system of the University of Auckland and at the New Zealand Blood Bank.

Recruitment setting
Participant recruitment took place at the Faculty of Medical and Health Sciences, University of Auckland, 85, Park Road, Grafton, Auckland 1023, New Zealand.

Settings and locations where the data and blood samples were collected
As above.

4. Intervention
Content: what was given?
An initial blood sample collected at baseline was used to measure serum selenium levels. Men having an inherent serum selenium level <200ng/ml were given the first three months of selenium supplements (90 capsules each containing 200µg Se as selenized yeast. These men were given instructions on who to report in the case of adverse incidents. They were instructed to send back a completed compliance sheet a week before the end of three months (11th week since start of supplementation) with whatever recorded changes observed since supplementation. Those recording no adverse events were supplied with an additional three month supply with the same instructions.

Content and delivery method
Supplementation was one capsule a day containing selenized yeast equivalent to 200µg selenium. This supplement was provided to us as SEL-PLEX from Alltech, Ireland, sourced from Republic of Serbia.

Unit of delivery
Delivery was non-randomised, therefore all recruited participants, received the supplement.

Deliverer
Supplements were delivered by Dr. Nishi Karunasinghe (the study Co-ordinator and Co-Investigator), under the supervision of Prof. Lynnette R Ferguson (New Zealand Nutrition Society, Registered Nutritionist (Scientific research) since 2001).

Setting: where was the intervention delivered
The first batch of three month worth supplements were delivered on-site at the Faculty of Medical and Health Sciences except for the participants from the New Zealand Blood Bank, who received them by post. The second batch was delivered by post to all after receiving and checking compliance and side effect recording.

Exposure quantity and duration-
200µg selenium per day for a total duration of six months.

Time span-
A total of six months or 180 days.

Activities to increase compliance or adherence
No monetary incentives were given to increase the compliance; instead, each participant had several

opportunities to understand the reasons behind the study. These included-

1. Email or postal mail communication to answer participant queries before and during the study.
2. A one-on-one communication for 25-30mins at the baseline visit where there was an information sharing process about the reasons behind this intervention study.

5. Objectives-

Specific objectives and hypotheses-

We know that low levels of micronutrients such as selenium (Se) enhance the risk of cancer, and may be a particular problem in New Zealand, which has low Se in its soils. Recent work suggests that small changes in genes, called single nucleotide polymorphisms or SNPs, may affect the amount of Se required by individuals. There is reason to believe that there are a small number of individuals in New Zealand and other countries, who are cancer prone because they require a higher than normal intake of Se. We intended to screen a sample of the population to consider whether supplementation with 200µg Se per day, in the form of selenized yeast, can help to overcome the associated deficiencies especially among men with specific genotypes.

- Define, how baseline serum Se and supplemented Se impacts the selected biomarkers of the participants both with and without stratifications based on demographic and lifestyle characteristics.
- Consider how the different genotypes respond to Se supplementation at a single level for 6 months, in terms of serum Se levels, DNA damage levels, and the activity of selenoenzymes glutathione peroxidase (GPX) and thioredoxin reductase (TR) and other biomarkers of cancer risk.
- Consider how the different genotypes respond to Se supplementation at a single level for 6 months, in terms of their proteome, metabolome and mineral profiles.

Our hypothesis is that supplementation of 200µg/d selenium as selenized yeast can modulate prostate cancer risk markers at least among sub-groups of New Zealand men.

6. Outcomes

Primary and secondary outcomes-

Approximately one third of all male deaths in New Zealand, are caused by cancer (in 2005 31% and 2015 32%)[22]. This is a huge public health burden for New Zealand. The Se intervention was to collate information necessary for determining personalised selenium levels for optimum benefits for New Zealanders bringing about a reduction in cancers caused by selenium deficiencies in men, especially prostate cancer.

Therefore, the primary outcomes were measured through surrogate biomarkers that can potentially induce cancer risk including the DNA damage levels and the deficiency of activity levels of seleno antioxidant enzymes GPX and TR (lower levels with potential to impair oxidative stress). These were assessed against pre- and post- Se levels.

Among the secondary endpoints measured were quantitation of apoptosis levels in a sub-cohort and serum levels of prostate-specific antigen levels in all available samples as a surrogate marker for prostate microarchitecture stability. Further secondary outcomes that are to be assessed include changes taking place due to Se supplementation, including that of the proteome, metabolome and trace mineral profiles.

Analysis of the above data stratified against seleno and other genome wide genotypes are to be implemented to understand genetic variation in supplementation benefits.

Methods used to collect data and any methods used to enhance the quality of measurements-

Participants visited the study centre at the Faculty of Medical and Health Sciences, University of Auckland for both the baseline and six month post-supplemented time points. At the baseline time point participants provided their informed consent, and submitted a completed health and lifestyle questionnaire. They also provided blood samples for serum selenium and biomarker assays and for DNA extraction for genotyping. They posted back the four day diet and activity diary records. The initial three month supplements of selenium were either handed over to the participants at the study centre or were posted only if their baseline serum Se level was <200ng/ml. As a safety issue, three out of 571 men took part in the baseline assessments were denied supplements as their serum Se level was beyond 200ng/ml. Participants posted back a compliance assessment along with notification of any changes observed during the initial 11 weeks of supplementation. If no significant side effects were notified, the second set of three month supple of Se was posted to the participants. Those recording adverse events were requested to stop supplementation. At the 23rd week, men contacted the study centre for organising the final blood draw and submission of compliance and adverse event records.

Processing of supplemented bloods and measurements-
Blood collected at each study visit was kept on ice until processed within 4 hrs of collection. One millilitre of heparinised and EDTA bloods were aliquoted and stored for future use in a -80^{0}C freezer. The rest of the EDTA and heparin bloods and the bloods collected in a plain tube were centrifuged at 2000 x g for 10 min at 4^{0}C. Plasma and serum aliquots were stored in a -80^{0}C freezer.

DNA extraction
An aliquot of 300µl of EDTA blood was used for DNA extraction using a QIAamp genomic DNA kit (Qiagen, Hilden, Germany) following the manufacturers' protocol with the aid of a fully automated QIAcube (Qiagen, Hilden, Germany). These DNA samples were stored in a -20^{0}C freezer for future genotyping purposes.
SNP genotyping
Genotyping was carried out using the Sequenom or Taqman methods as described previously [10; 23] with no template and blank controls as well as HapMap controls.
DNA damage measurements
An aliquot of 20µl of heparinised blood was set aside for DNA damage analysis using the procedures recorded by us previously [23; 24]. Microgels containing blood cells were prepared on the same day and lysed overnight, before denaturing, electrophoresis and neutralising on the following day. DNA damage levels were quantitated using the procedures mentioned by us before [23; 24]. A fluorescent Zeiss Axioskop2 microscope fitted with100W mercury burner from Osram, an excitation filter of 515–560nm and a barrier filter of 590nm, Evolution VF cooled monochrome camera kit from Media Cybernetics Bethesda, USA and Komet 6.0 Single Cell Gel Electrophoresis Analysis software from Andor Technology, Ireland were used in the DNA damage quantitation process. The serum levels of selenium was measured in batches in all baseline samples within two months of sample collection. These measurements were made at the Gribbles Veterinary Pathology, Hamilton, New Zealand using a modified semi-automated fluorometric assay based on Watkinson [25], Watkinson and Brown [26] and Rongpu et al [27]. The fluorescence of the final benzopiazselenol extracted into cyclohexane was measured with an excitation wave length of 360nm and emission wave length of 518nm.The selenium assay recorded 2.6% intra assay coefficient of variation and 11.2% inter assay coefficient of variation.
Seleno enzyme measurements
An aliquot of 100µl EDTA blood was used to prepare hemolysates as reported by us before [28] and stored at -80^{0}C freezer for selenoenzyme activity measurements.
The activities of GPX and TR levels were measured using previously published protocols[28].
Apoptosis measured as caspase-cleaved K18 levels and was were carried out using two M30 Apoptosense ELISA solid-phase sandwich enzyme immunoassay kits, from Peviva, VLVbio, Sundbyberg Sweden, using manufacturer's protocol.

PSA measurements

Total PSA was measured in remaining baseline and post-supplemented serum aliquots at LabPlus, Auckland, New Zealand using electrochemiluminescence immunoassay (Roche Cat. #. 04641655 190) on a Roche Modular E170 anaylser (Roche Diagnostics, NZ). Total assay imprecision was 3.2% at a level of 1.12 ng/mL, 3.7% at 4.61 ng/mL, and 2.7% at 27.5 ng/ml.

Diet data analysis

At study entry, participants were provided with four-day diet and activity diary forms with specific instructions on completing them on four consecutive days, preferably including one weekend day. Participants were requested to select their regular activity pattern, from seven categories given with the diary recording sheets. These included, very sedentary, sedentary, light, light moderate, moderate, heavy and very heavy. The four-day diet and activity diary data along with the BMI estimated at baseline and age at recruitment were uploaded to the Foodworks Professional Version 9 (Xyris software). It was noted that some items listed in the food diaries lacked details in terms of the food item itself and the quantity. These data were independently checked for possible variable entries of food and drink quantity and quality. Based on these checks, a list of such commonly variable-entries on items and unknown quantities transferred to the FoofWorks Database were identified and listed. Default entries to be used in such circumstances were listed (Supplementary Table 1). General rules for Foodworks data entry were also written up (Supplementary document 1). Using the above table and rules, all data entries without sufficient information on quantity and quality of dietary items were independently standardised. Records that lacked sufficient information or provided unacceptable recordings (eg. basal metabolic rate being higher than the energy provided by the diet) were removed from the analysis. A total of 192 participants failed to provide diet and activity data recording while 37 were removed from analyses due to lack of adequate information. Therefore, only a total of 343 diet and activity records were considered for subsequent analyses.

Information on validated instruments such as psychometric and biometric properties

Not applicable

7. Sample Size determination

In order to estimate the required sample size for this study, a simple parametric t-test approach was assumed. Although more sophisticated statistical methods was to be applied to the data, this simple assumption provides a reasonable platform for the calculation of power. Performing a paired sample t-test (samples taken before and after Se supplementation) for each SNP genotype, for each of the six genes (originally planned) requires 18 hypothesis tests. Power calculations are based on use of the Bonferroni adjustment to account for multiple testing, under the assumption of test independence. Although the tests are likely to be dependent, treating them as independent will result in a conservative estimate of sample size requirements, making it likely that power is underestimated here. Based on the data of Gill et al.[29] a sample standard deviation of 10 is assumed for the power calculations. For a two-sided paired sample t-test using an alpha level of 0.05/18=0.00278 (Bonferroni correction), 23 samples are required to detect a change in response of 10 (25%) with a power of greater than 90%. Assuming a 15% drop-out rate, this level of power can be achieved with 27 patients per SNP genotype. SNP genotype frequencies in the preliminary data indicate that the rarest genotype (homozygous LL in GPX1) occurs in approximately 0.047 of the population. To achieve an expected sample size of 27 for this genotype requires a patient population of 27/0.047=574, which is slightly less than the proposed sample size of 600. Thus, based on the available data, a sample of 600 patients is expected to provide at least 90% power to detect a 25% change in response in any of the 18 SNP genotypes under investigation, with less than a 5% chance of any false positives across any of the 18 tests.

8. Assignment Method

Unit of assignment (the unit being assigned to study condition, e.g., individual, group, community)

This study was carried out with one Auckland, New Zealand cohort that received the same dose of Se without any randomisation. Therefore, instead of comparing between a control arm and a treatment arm, comparisons were carried out between baseline and post-treatment time points.

Method used to assign units to study conditions, including details of any restriction (e.g., blocking, stratification, minimization)
Not applicable

Inclusion of aspects employed to help minimize potential bias induced due to non-randomization (e.g., matching)
Being a non-randomised study inclusion and exclusion criteria were applied to minimize bias. Additionally, data analysis with stratifications based on age, BMI, tobacco smoking and alcohol consuming lifestyles, health status and stratifications based on genetic factors were employed. Further adjustments of results were made for age, BMI, tobacco smoking and alcohol intake lifestyle factors as necessary.

9. Blinding-

Blinding was not required as all participants received the same dose of supplements covering the six month period.

10. Unit of Analysis-

Unit of analysis varied depending on the available outcome tested.

Number of available data points

	Baseline	Post-supplementation
Serum Se	571	481
Glutathione peroxidase activity-in hemolysates	565	480
Thioredoxin reductase activity-in hemolysates	565	479
Leukocyte DNA damage	562	473
Leukocyte DNA damage with a peroxide challenge	561	470
Prostate-specific antigen in serum	498	420
Caspase-cleaved K18 levels in plasma	19	19
Diet and activity diaries	380 (submitted), 343	
SNP genotyping	520-570	

Data analysis included adjustments for age, BMI, tobacco smoking and alcohol consumption lifestyles when required.

Statistical methods-

11. Statistical methods used for previously published results-

Analysis on the relationships between serum Se and biomarkers with and without stratification for genotypes were examined using Pearson's correlation statistics for simple correlations. Multiple regression analyses were carried out using generalised linear models (GLM), using the GLM procedure in SAS. Whether the relationship between the outcome and the predictor of serum Se concentration was linear was tested by fitting a quadratic polynomial. The GPx1 rs1050450 C/C and GPx4 rs713041 T/T genotypes show significant nonlinearity with DNA damage, so a 'broken stick' regression was fitted, with different linear regression models in two different regions constrained so that the fitted curve was continuous at the break point concentration. The optimal break point was chosen by taking the concentration which gave rise to the lowest RSS (Residual Sum of Squares). Variations between baseline and post-supplementation data for variables were compared using the Wilcoxon signed rank test with pooled ranks from both time points with and without adjustments for age, BMI, tobacco smoking and alcohol consumption lifestyle.

Statistical methods on the current manuscript

Continuous variables of age, BMI, serum selenium and PSA levels between all participants at baseline and those completing supplementation protocol were tested using the Mann-Whitney Rank Sum Test, as the data were not normally distributed. Categorical variables of tobacco smoking and alcohol consumption lifestyles between the baseline and protocol completing groups were tested with the F-statistics. Health disorders and the food and activity record submission details of these two groups were tested with the Chi Square statistics. The distribution of genotypes in the panel of SNPs were tested for biallelic distribution using the Hardy Weinberg equilibrium calculator [(30)]. Variation of baseline and post supplemented levels of serum selenium and serum PSA between genotype groups of each genetic polymorphism were compared using the Analysis of Variance (ANOVA) on Ranks as the variables were not normally distributed. All correlations were tested using the Spearman Rank Order Test. The correlation between changes in serum selenium levels and changes in serum PSA levels subsequent to selenium supplementation was assessed. These were further stratified by the homozygous dominant, heterozygous and homozygous recessive genotypes of the selected genotypes. Correlations in changes in serum selenium and changes in serum PSA levels subsequent to supplementation was also analysed with stratifications based on the dietary intakes below or above the recommended dietary intakes(RDI)[(31)]of selenium, zinc, Vitamin B12, and folate and below or above the median of the % energy derived from dietary proteins. Dietary intake cut-offs for selenium, Zn, Vitamin B12 and dietary folate equivalents were considered as 70µg/d, 14mg/d, 2.4µg/d and 400µg/d respectively as given by the joint report from the National Health and Medical Research Council, Australia and New Zealand Ministry of Health[(31)]. The cut-off for the % energy intake from protein was considered as the median value of 16.0% recorded in the current cohort. The Mann-Whitney Rank Sum Test, ANOVA on Ranks, Chi Square test, F statistics and the Spearman Rank Order Correlation test were performed using SigmaPlot version 14.0 (Systat Software Inc.). A significance level of $P<0.05$ was set out for all analyses.

12. Participant flow

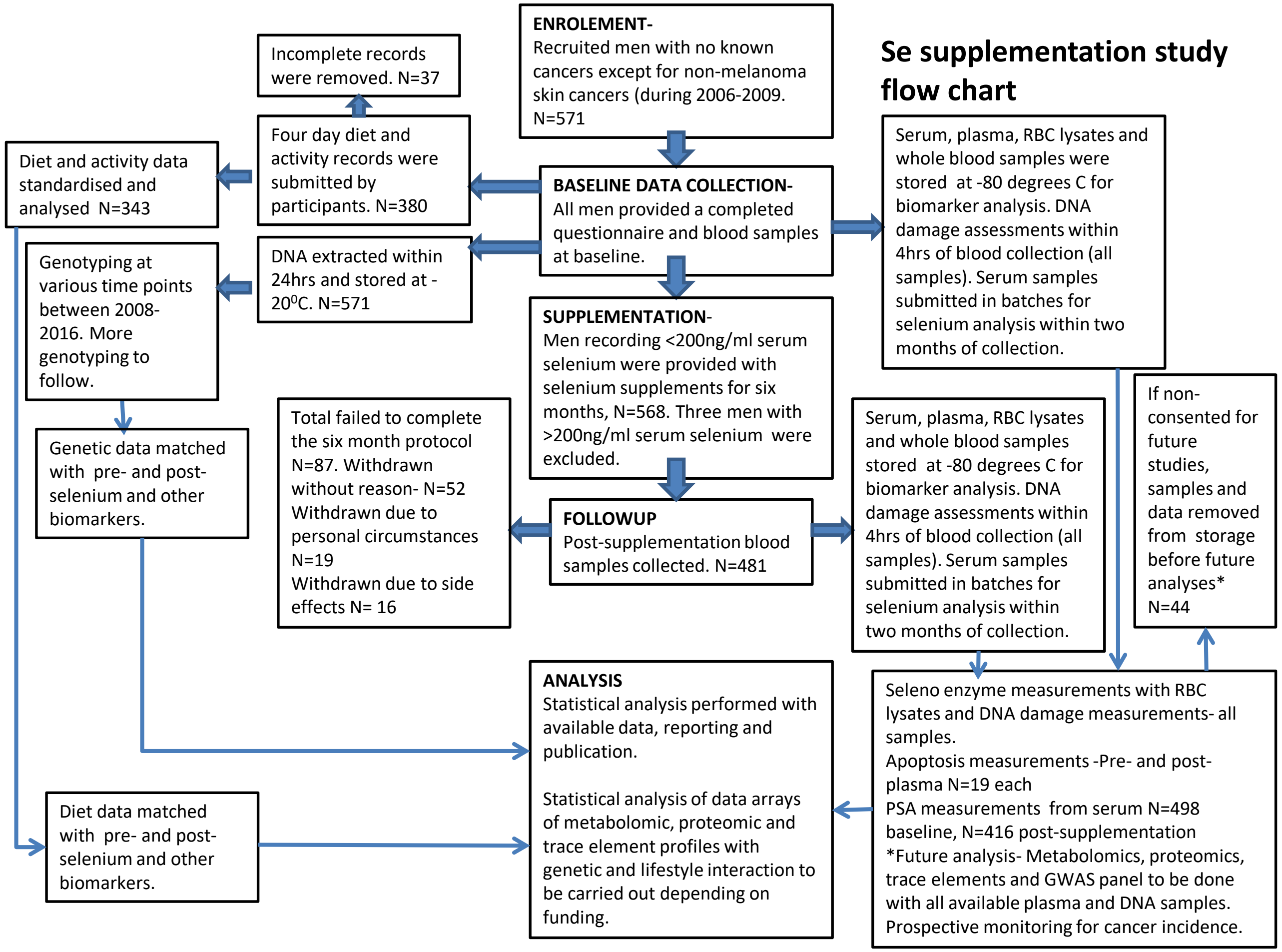

Enrolment-

All eligible participants were enrolled with informed consent.

Assignment: the numbers of participants assigned to a study condition
All eligible participants were assigned to study condition if their inherent or baseline serum Se level was <200ng/ml.

Allocation and intervention exposure: the number of participants assigned to each study condition and the number of participants who received each intervention.
A total of 571 men were checked for baseline serum Se level and a total of 568 men were eligible to receive supplements.

Follow-up: the number of participants who completed the follow- up or did not complete the follow-up (i.e., lost to follow-up), by study condition.
A total of 481 completed the six month supplementation protocol while 87 failed to complete the protocol for various reasons. A total of 16 were requested to withdraw due to adverse conditions, 19 withdrew due to personal circumstances, while 52 have withdrawn without providing a reason.

Analysis: the number of participants included in or excluded from the main analysis, by study condition
As above.

Description of protocol deviations from study as planned, along with reasons.
A baseline serum Se level above 200ng/ml was not anticipated from a New Zealand cohort. When three men recorded levels above this limit, we declined giving them supplements for safety reasons.

13. Recruitment- Dates defining the periods of recruitment and follow-up

Recruitment of men to this study started on 16 October 2006 and the last participant was recruited on 22 December 2008. The last participant concluded the post-supplementation study visit on 13 August 2009.

14. Baseline data-

Baseline demographic and clinical characteristics of participants in each study condition.

Table 1. Participant summary characteristics at baseline and those completed supplementation protocol.

Character		Baseline	Protocol completed	P value
		Median (25th and 75th percentile) [number]	Median (25th and 75th percentile) [number]	
Age (years)		54 (44.0, 63.8) [572]	55 (46.0,65.0) [481]	0.11
BMI (kg/m^2)		26 (24, 29) [548]	26 (24, 29) [463]	0.93
Baseline serum Selenium level		110.5 (94.7, 126.3) [571]	110.5 (98.7, 126.3) [481]	0.62

ng/ml				
Baseline serum PSA level ng/ml		0.9 (0.6, 1.9) [498]	1 (0.6, 2.0) [416]	0.70
Tobacco smoking (number & %)	Ever	190 (33.2)	166 (34.5)	1.00
	Never	382 (66.8)	315 (65.5)	
Alcohol consumption (number & %)	Yes	492 (86.0)	416 (86.5)	1.00
	No	80 (14.0)	65 (13.5)	
Health disorders	None	348	286	0.23
	Cardiovascular	119	104	
	Diabetes	6	6	
	Depression/Anxiety	18	15	
	Inflammatory	26	20	
	Urology	30	29	
	Other	25	21	
Food & activity diary submission (number & %)	Accepted submissions	343 (60)	320 (66.5)	0.20
	Rejected submissions	37 (6.5)	28 (5.8)	
	Not submitted	192 (33.6)	133 (27.7)	

Baseline characteristics for each study condition relevant to specific disease prevention research

This study consisted of one study condition where all eligible men received the supplement at least for three months except those withdrawn from the study due to various reasons.

Baseline comparisons of those lost to follow-up and those retained, overall and by study condition

Table 2. Comparison of baseline biomarker levels of those completing the study and those who dropped out

	N	Mean (SE)				
		Serum Se ng/ml*	GPx activity mU/mg Hb	TR activity mU/mg Hb	Basal DNA damage	Peroxide-induced DNA damage
Baseline marker levels						
All participants	503	111.5 (1.01)	14.4 (0.32)	1.06 (0.04)	6.02 (0.06)	7.54 (0.08)
Those who completed the study	425 (84.5)	111.9 (1.01)	14.7 (0.36)	1.05 (0.04)	6.03 (0.07)	7.57 (0.09)
Those who did not complete the study	78 (15.5)	109.1 (1.03)	12.8 (0.65)†	1.07 (0.09)	5.95 (0.16)	7.40 (0.19)

* To convert serum Se to µmol/L, multiply by 0.0127

†-Significantly lower p=0.034

Comparison between study population at baseline and target population of interest

The current study cohort recorded a daily dietary intake average of Se equivalent to 74+/-43µg (or a median of 64.8µg/d), whereas the average recorded for New Zealand males is 67µg/d (or a median of 65µg/d) [32]. This indicates that the study population and the target population has similar dietary intake levels of Se.

15. Baseline equivalence

Data on study group equivalence at baseline and statistical methods used to control for baseline differences

Baseline differences by way age, BMI, tobacco smoking lifestyle and alcohol consumption were adjusted where necessary.

16. Numbers analysed-

Number of participants (denominator) included in each analysis for each study condition, particularly when the denominators change for different outcomes; statement of the results in absolute numbers when feasible

Total recruited- 571

Total submitted baseline blood samples and health and completed lifestyle questionnaires- 571

Total submitted four day diet and activity diaries- 343

Total provided with Se supplements- 568

Total completed the six month study protocol- 481

Table 3. A breakdown of different data points available for analysis.

	Baseline	Post-supplementation
Serum Se	571	481
Glutathione peroxidase activity-in hemolysates	565	480
Thioredoxin reductase activity-in hemolysates	565	479
Leukocyte DNA damage	562	473
Leukocyte DNA damage with a peroxide challenge	561	470
Prostate-specific antigen in serum	498	420
Caspase-cleaved K18 levels in plasma	19	19
Genotyping		
Diet and activity diaries	343	

SNP genotyping	520-570	

Indication of whether the analysis strategy was "intention to treat" or, if not, description of how non-compliers were treated in the analyses.

This analysis strategy was 'intention to minimize prostate cancer risk'.

17. Outcomes and estimations-

For each primary and secondary outcome, a summary of results for each estimation study condition, and the estimated effect size and a confidence interval to indicate the precision

Table 4. The supplementation effects on serum Se level and Biomarkers after adjustment for demographic, lifestyle and health factors

	Treatment	Estimate (SE)	p
Serum Se	after	40.2 (2.20)	**< 1E-29**
	before	0	
GPx activity	after	-0.84 (0.43)	**0.0478**
	before	0	
TR activity	after	0.35 (0.06)	**5.42E-10**
	before	0	
Basal DNA damage	after	0.99 (1.02)	0.6234
	before	1.00	
Peroxide-induced DNA damage	after	1.07 (1.02)	**7.55E-04**
	before	1.00	

Table 5. The interaction of Se supplementation (before and after) with lifestyle and health factors and the subsequent marker variability

		Serum Se		GPx activity		TR activity		Basal DNA damage		Peroxide-induced DNA damage	
		Estimate (SE)	p	Estimate (SE)	p	Estimate (SE)	p	Estimate (SE)	p	Estimate (SE)	p
Smoking status * after treatment	Ever	-0.46 (4.51)	0.919	1.91 (0.89)	**0.031**	0.06 (0.12)	0.622	0.997 (1.03)	0.915	1.03 (1.04)	0.473
	None	0		0		0		1.00		1.00	
Current Alcohol intake * after treatment	Yes	-10.92 (6.37)	0.087	-0.27 (1.12)	0.807	-0.19 (0.15)	0.216	0.96 (1.05)	0.353	1.01 (1.06)	0.828
	No	0		0		0		1.00		1.00	
Health condition * after treatment	Cardiovascular	-3.74 (5.83)	0.521	-1.51 (1.06)	0.153	-0.09 (0.14)	0.521	1.01 (1.04)	0.806	0.91 (1.04)	**0.027**
	Mental illness	-9.88 (14.51)	0.496	-1.02 (1.28)	0.425	0.45 (0.26)	0.079	0.98 (1.09)	0.821	1.02 (1.15)	0.868
	Diabetes	-0.68 (13.63)	0.960	3.30 (4.25)	0.438	-0.22 (0.39)	0.579	1.24 (1.17)	0.182	1.02 (1.20)	0.916
	Urology	14.13 (7.65)	0.065	2.49 (1.68)	0.140	-0.09 (0.27)	0.754	1.05 (1.06)	0.369	0.98 (1.09)	0.828
	Other	5.16 (7.79)	0.508	0.22 (1.56)	0.890	-0.1 (0.18)	0.573	1.01 (1.06)	0.825	1.04 (1.09)	0.673
	Healthy control	0		0		0		1.00		1.00	

Table 6. Gene-biomarkers association after adjustment for demographic, lifestyle and health factors

Gene	SNP	Tested allele	Treatment	Serum Se		GPx activity		TR activity		Basal DNA damage		Peroxide-induced DNA damage	
				Estimate (SE)	p	Estimate (SE)	p	Estimate (SE)	p	Estimate (SE)	p	Estimate (SE)	p
GPX1	*rs1050450*	*C*	Allele effect	-0.746 (2.358)	0.752	1.339 (0.468)	**0.0043**	0.091 (0.060)	0.134	1.008 (1.017)	0.611	1.006 (1.020)	0.756
			after	2.887 (3.424)	0.399	-0.618 (0.687)	0.369	-0.107 (0.089)	0.229	0.980 (1.025)	0.409	1.015 (1.029)	0.597
			before	0		0		0		1.00		1.00	
GPX4	*rs713041*	*C*	SNP effect	-0.152 (2.053)	0.941	-0.055 (0.417)	0.896	0.038 (0.053)	0.479	1.003 (1.015)	0.845	0.988 (1.017)	0.468
			after	3.610 (2.987)	0.227	-0.387 (0.609)	0.525	-0.086 (0.078)	0.269	1.007 (1.022)	0.749	1.026 (1.026)	0.319
			before	0		0		0		1.00		1.00	
SELS	*rs28665122*	*A*	SNP effect	-2.305 (2.784)	0.408	-0.461 (0.731)	0.529	-0.065 (0.089)	0.469	1.00 (1.026)	0.996	1.038 (1.031)	0.228
			after	2.525 (4.213)	0.549	-0.074 (1.104)	0.947	0.059 (0.135)	0.663	0.988 (1.04)	0.765	0.950 (1.049)	0.291
			before	0		0		0		1.00		1.00	
SELS	*rs4965373*	*A*	SNP effect	4.543 (2.201)	**0.039**	-0.024 (0.440)	0.957	0.004 (0.056)	0.948	1.004 (1.016)	0.796	1.003 (1.018)	0.872
			after	-2.801 (3.200)	0.382	-0.766 (0.647)	0.236	-0.128 (0.083)	0.124	0.967 (1.023)	0.137	1.003 (1.027)	0.901
			before	0		0		0		1.00		1.00	
SEP15	*rs5845*	*T*	SNP effect	0.587 (2.708)	0.829	0.445 (0.544)	0.414	0.050 (0.070)	0.478	1.001 (1.019)	0.954	1.001 (1.023)	0.981
			after	1.733 (3.910)	0.658	-1.445 (0.788)	0.067	-0.145 (0.101)	0.154	1.005 (1.028)	0.857	0.982 (1.034)	0.588
			before	0		0		0		1.00		1.00	
SEPP1	*rs3877899*	*A*	SNP effect	-4.215 (2.538)	0.097	-0.090 (0.507)	0.86	0.055 (0.065)	0.394	0.996 (1.018)	0.810	0.966 (1.021)	0.095
			after	-0.121 (3.662)	0.974	0.319 (0.744)	0.668	-0.229 (0.096)	**0.017**	1.028 (1.027)	0.302	1.044 (1.032)	0.175
			before	0		0		0		1.00		1.00	

Table 7. Dietary intake recorded for subgroups A (with the highest increase in H_2O_2-induced DNA damage) and B highest decrease in H_2O_2-induced DNA damage subsequent to Se supplementation.

	Group A	Group B	95% CI	*p* Value
Macro nutrients: Mean (SD) or Median and percentiles [1]				
kj-from-protein_%	15.69 (2.29)	15.54 (2.70)	−1.80 to 2.09	0.878
kj-from-carbohydrates_%	43.15 (6.60)	44.49 (8.96)	−7.45 to 4.77	0.656
kj-from-total fats_%	34.92 (30.20,42.33) [1]	31.69 (30.64, 37.11) [1]		0.241
fat-as-saturated_%	41.90 (8.74)	45.84 (5.51)	−9.62 to 1.74	0.26
fat-as-poly_%	20.78 (7.71)	16.54 (5.13)	−0.86 to 9.34	0.099
fat-as-mono %	37.32 (4.17)	37.62 (3.26)	−3.20 to 2.61	0.834
Micro-nutrients: Mean (SD) or Median and percentiles [1]				
Selenium µg/day	60.15 (27.88)	59.38 (22.50)	−9.80 to 28.74	0.322
Vitamin B12 µg/day	4.18 (3.54,7.54) [1]	5.28 (3.28,7.82) [1]		0.629
Vitamin B6 mg/day	1.70 (1.54, 2.23) [1]	1.81 (1.56, 2.14) [1]		0.629
Zinc mg/day	12.39 (11.57,14.60) [1]	15.03 (12.32, 16.14) [1]		0.103
Total folate µg/day	504.79 (370.1,616.30) [1]	348.49 (298.98, 461.74) [1]		0.046
Methionine g/day	0.75 (0.37)	1.09 (0.44)	−0.65 to −0.02	0.039

[1] = Median and 25th and 75th percentiles; kj—kilo joules.
Group A (N=19) - Men who showed the highest increase in H_2O_2-induced DNA damage after supplementation
Group B (N=19) - Men who showed the highest decrease in H_2O_2-induced DNA damage after Se supplementation

Table 8. Caspase-cleaved K18 levels in plasma [units per litre mean (SD)] between subgroups A (with the highest increase in H_2O_2-induced DNA damage) and B highest decrease in H_2O_2-induced DNA damage and between baseline and post-supplementation time points.

	Group A	Group B	95% CI	*p* Value
Pre-	182.38 (67.5)	222.24 (70.2)	−87.215 to 7.496	0.0962
Post-	229.78 (82.5)	255.69 (79.8)	−81.790 to 29.966	0.352
	***p* = 0.0428**	***p* = 0.0218**		

Pre- = Baseline; Post- = Post-supplementation; *p* values between pre- and post-supplementation comparisons are underlined.

Group A (N=19) -A subgroup of nineteen men that showed the highest increase in H_2O_2-induced DNA damage after supplementation

Group B (N=19) - A subgroup of nineteen men that showed the highest decrease in H_2O_2-induced DNA damage after Se supplementation

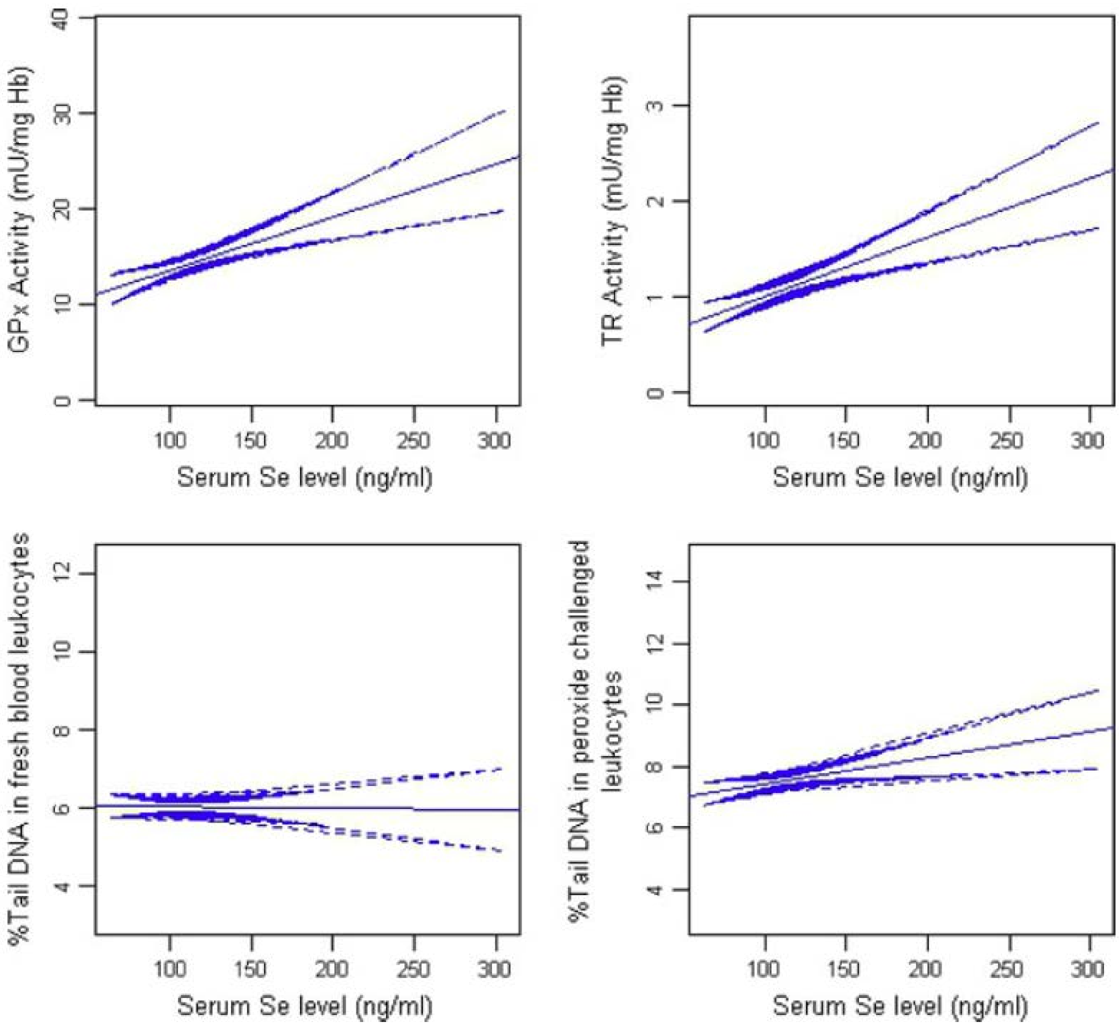

Figure 1- Variation of biomarker levels with serum Se level among all subjects at baseline (line fitted with 95% CI)

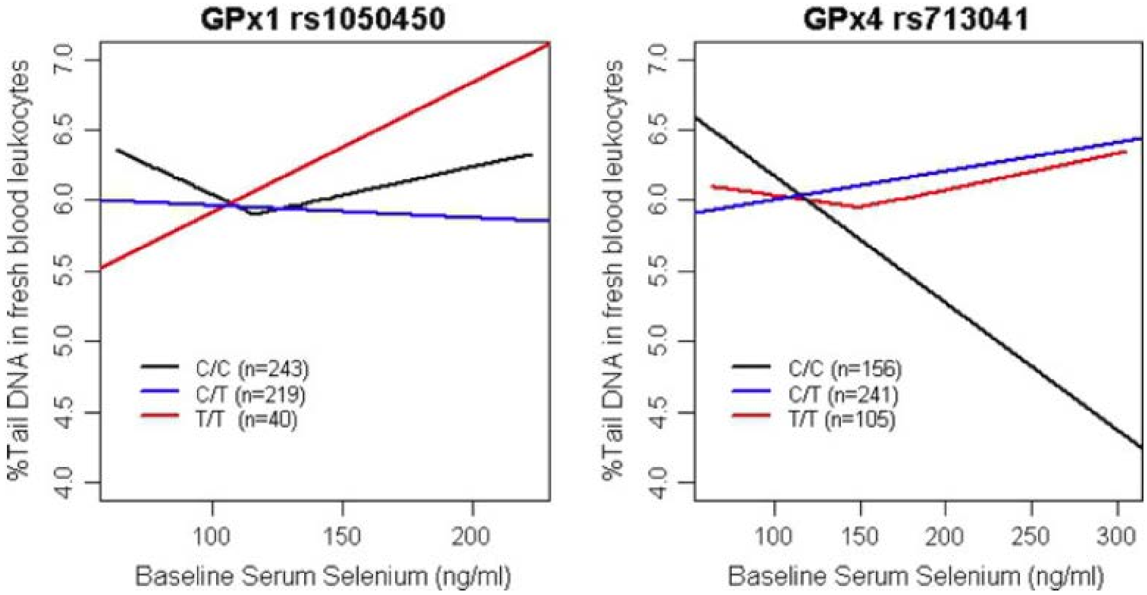

Figure 2 - Variation of biomarker levels with serum Se level among all subjects, at baseline according to genotype (line fitted with 95% CI), for those data sets best fitted by a broken stick regression. GPx1 rs1050450 C/C and GPx4 rs713041 T/T genotypes were better fitted using the broken stick regression with the lowest RSS at 116.07 and 149.23 ng/ml serum Se respectively (*P* = 0.044 and *P* = 0.042, respectively)

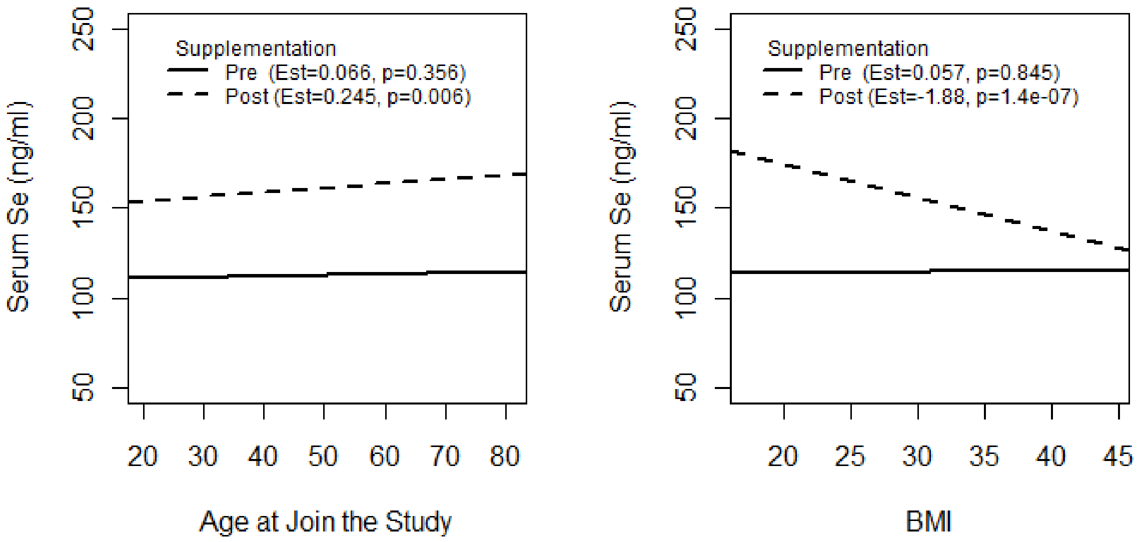

Figure 3. Serum Selenium association with age at joining the study and BMI pre- and post-supplementation

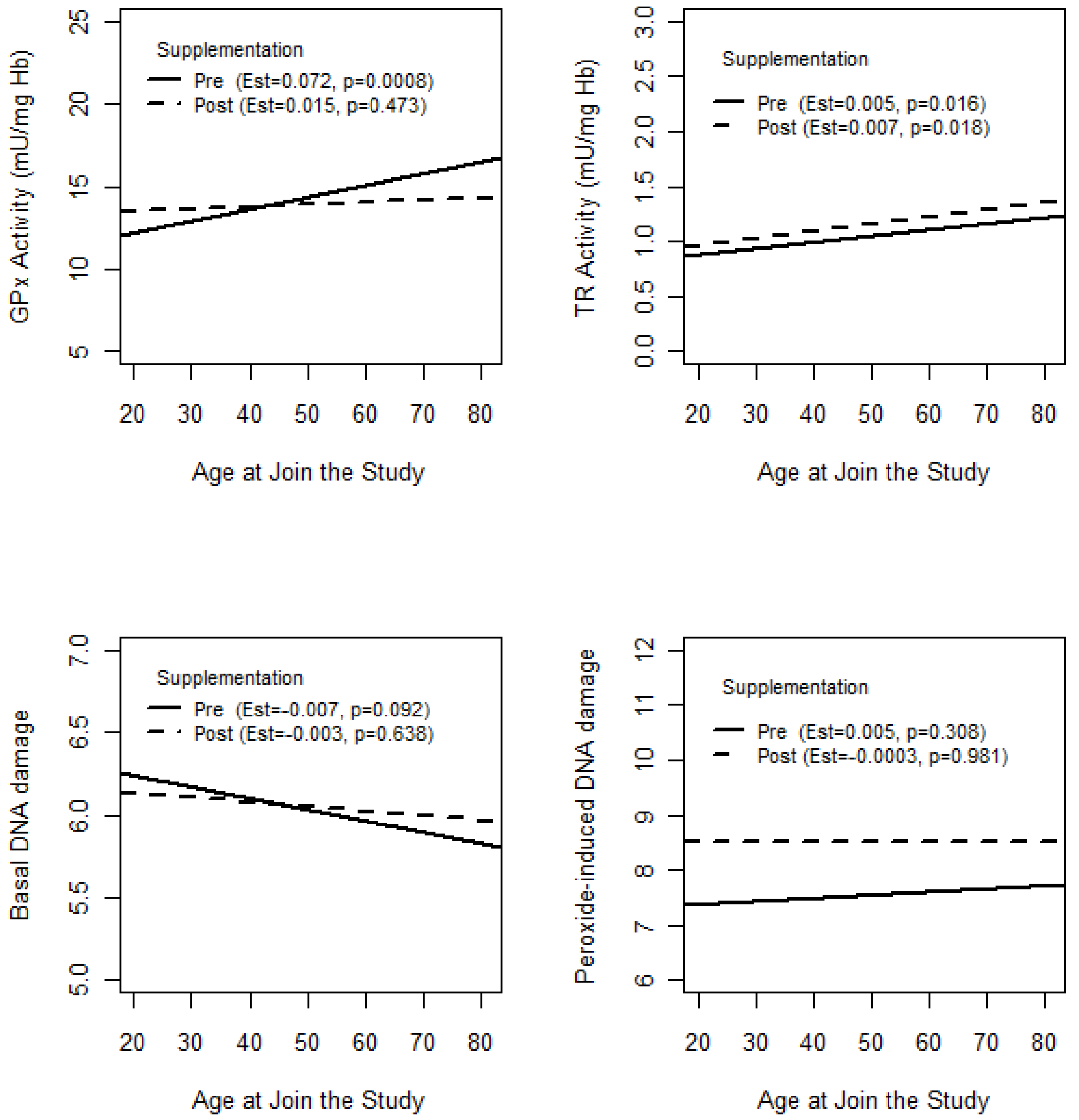

Figure 4. Association among biomarkers and age at joining the study pre- and post-supplementation

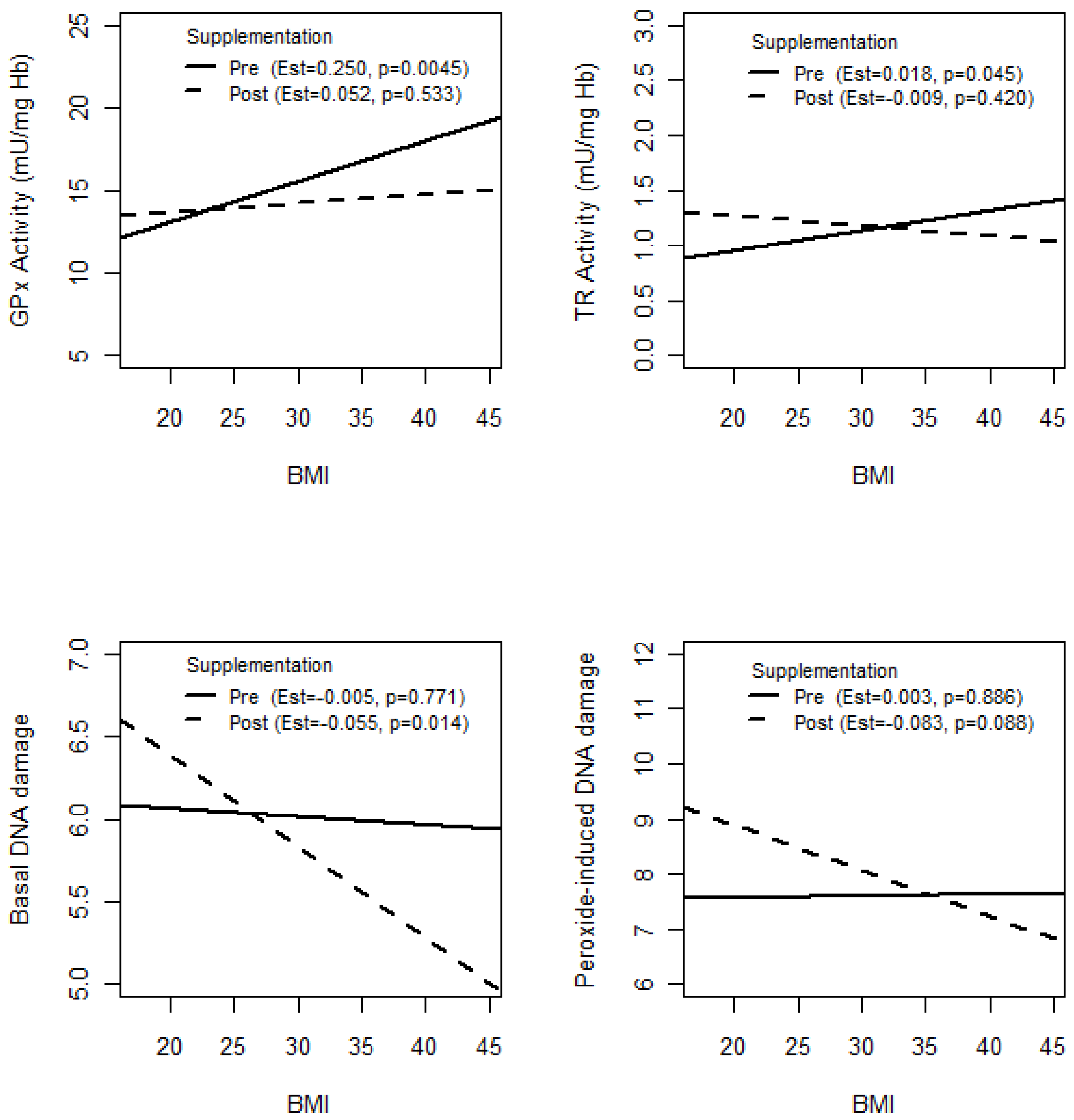

Figure 5. Association among biomarkers and BMI, pre- and post-supplementation

An overview of additional results not yet published-

Correlation between changes in serum Se due to supplementation and corresponding changes taking place in serum PSA levels. The correlation statistics are available both with and without stratification based on participant demographics, lifestyle, and a panel of genotypes as well as dietary intake of a panel of nutrients.

Inclusion of null and negative findings

None

Inclusion of results from testing pre-specified causal pathways through which the intervention was intended to operate, if any

Although Se is at the activity centre in the GPx protein, supplementation did not increase the GPx activity levels overall, indicating GPx activity is saturated associated with a lower serum Se levels compared to TR activity.

18 Ancillary analyses

Summary of other analyses performed, including subgroup or restricted analyses, indicating which are pre-specified or exploratory

Se supplementation dependent plasma Caspase-cleaved K18 levels and serum PSA levels were measured as ancillary analysis. The caspase-cleaved K18 was analysed due to its apoptotic potential indication while PSA levels were assesses as a surrogate marker for prostate gland microarchitecture stability. Additional ancillary analysis based on proteomic, metabolomic and trace mineral profiles, pre- and post-supplementation are also planned.

19 Adverse events

Summary of all important adverse events or unintended effects in each study condition (including summary measures, effect size estimates, and confidence intervals)

Positive or Adverse events/harms-

Table 9. Self-Reported conditions of 533 participants since starting Se supplement among the participants. A total of 66 indicated no difference while others not having noted changes produced an empty assessment sheet. (A unit count is equivalent to any mentioning of a condition by a participant.)

No difference reported	66
Blood pressure dropped	2
Firm stool	1
Faster wound healing	1
Better liver functions	1
Recovered liver functions	1
Ability to swim faster	1
Facial eczema reduced	1
Able to discontinue long term use of antibiotics	1
Large ganglion on hand disappeared	1
Less pain from hip arthritis	1
Able to go without antidepressants 2-3 month of Se intake	1
Better PSA level	1
Positive mood	1
Good urine flow	1
Increased agility/mobility	2
Increased growth of head hair	2
Heightened libido	2
Better skin	2
Increased finger and toe nail growth	3
Felt good and positive	7
	1
Resistant to cold and flu and minor infections	2
Sticky stool	1
Higher liver ALT	1
Reaction to insect bites	1
More thirsty	1
Passed more urine	1
Arterial fibrillation	1
Tightening of chest	1
Fluid retention	1
Funny feeling in genitals	1
Brittle nail	1
Blurred vision	1
Bad mood	1
Heart attack	1
Mouth ulcers	1
Throat inflammation	1
Increased blood pressure	2
Bad breath	1
Sulphurous body odour	1
Disturbed sleep	2
Facial acne	2
Weight increase	2
Nausea	2

Alopecia	2
Lack of concentration/unusual feeling/confusion	3
Tingling numbness or cold feeling in toes and fingers	3
Muscular cramps/aches/tightening	4
Unusual feeling	3
Itchy skin rash	3
Felt sick first 2-3days	3
Headache	4
Fatigue/tired	10
Abdominal discomfort/constipation/diarrhoea/wind/indigestion/reflux	14
Bad taste and smell	20

Table 10. Comparison of % adverse or positive events of Se supplementation between the current study and SELECT trial.

	Auckland trial (n=503)	SELECT Se only group (n=8752)
Alopecia	0.40	3.03
Faster growth of hair	0.40	
Dermatitis	0.60	7.07
Halitosis	0.20	5.75
Faster growth of nails	0.60	
Discoloration ridging or pitting*of nails	0.20	12.42
Fatigue*	1.79	7.61
Nausea*	0.40	2.89
Abdominal discomfort/constipation/diarrhoea/wind/indigestion/reflux	2.78	

*= Terminology used by National Cancer Institute- Common toxicity criteria
Auckland trial - Self reported changes within six month of supplementation
SELECT trial- Pre-specified adverse events known to be associated with Se Supplements

Discussion

20. Interpretation

Interpretation of the results, taking into account study hypotheses, sources of potential bias, imprecision of measures, multiplicative analyses, and other limitations or weaknesses of the study

Average baseline serum Se level recorded by NZ men with no known diagnosis of cancers was 112±24ng/ml [(19)] which is lower than the levels recorded by similar healthy men from US (European Americans (EA) and African Americans (AA) healthy men record 140±28 and 134±21 ng/ml, respectively) [(33)]. Our studies also record that serum Se levels among healthy men who were current smokers was

significantly lower (102±24ng/ml) compared to never (111.5±24) or past smokers (113±24ng/ml) [10]. According to our assessments, PC patients from NZ record lower serum Se levels (101 ± 22ng/ml) at diagnosis [19] compared to those from the US (135±21ng/ml and 132±25ng/ml for EA and AA respectively [33]) as well as compared to NZ men with no known diagnosis of cancers (112±24ng/ml) [19]. Our estimates indicate that the optimal Se level for better health based on DNA damage modulation between genotypes is between 120-150ng/ml of serum [10]. This estimate is comparable to the PC protective range suggested by Fairweather-Tait et al (>120 and <160ng/ml in serum or plasma) [1] and levels for decreasing mortality (135ng/ml) as suggested by Rayman et al [3]. However, among our healthy men 64% recorded baseline serum Se levels below 120ng/ml (reassessment of data from previous study [18]). Se supplementation increased the serum Se level, TR activity and H_2O_2 induced DNA damage, while decreasing GPx activity level. This indicates that GPx activity is saturated associated with a lower serum Se level but not the TR activity. Supplementation-related serum selenium level showed an increase with increasing age and a decrease with increasing BMI. With increasing age, GPx and TR activities increased at baseline, but upon supplementation, this increase was limited to the TR activity. With increasing BMI, both GPx and TR activities increased at baseline, but upon supplementation, this increase was lost. Increasing age and BMI can be associated with increased oxidative stress, which requires upregulation of antioxidant enzymes. However, upon Se supplementation, the overall oxidative stress is reduced, bringing about a reduction in these antioxidant enzymes. In our analysis, we see a reduction in DNA damage in fresh blood leukocytes as the BMI increases. This implies that the DNA reduction benefits get more prominent as the BMI increase. We also observe a genetic variation in DNA damage reduction with the GPx1 rs1050450 CC genotype showing the least DNA damage when serum Se reaches 116ng/ml while for the GPx4 rs713041 TT genotype this happens at 149ng/ml.

Sub-cohort analysis with the secondary outcome, the caspase-cleaved K18 showed that Se supplementation benefits for the leukocyte DNA integrity is accomplished only by those with lower dietary folate and higher dietary methionine intakes. For those with sufficient levels of folate intake and lower levels of methionine intake, Se supplementation was not favourable for the leukocyte DNA integrity. Se supplementation significantly increased the caspase-cleaved K18 levels overall, indicating increased homeostatic apoptotic potential of this supplement.

The most recent analysis of secondary data based on serum PSA level showed that reduction benefits varied based on the demographic and lifestyle, genetic and dietary factors (manuscript submitted for publication review).

Overall, this study managed to recruit the expected number of participants, but only 84% of participants managed to complete the supplementation protocol for various reasons. Compared to the Selenium and Vitamin E Cancer Prevention Trial (SELECT) where the supplementation mode was selenomethionine given to men with baseline serum Se levels around 135ng/ml, this New Zealand study that used selenized yeast in a cohort of men carrying relatively lower levels of baseline serum Se, recorded lower side effects. The most striking finding was, PSA reduction benefits were achieved by men carrying risk genotypes for prostate cancer. Given the fact that the serum PSA is a surrogate marker for prostate health, these findings call for attention to re-evaluate the cohorts taken part in the Nutrition Prevention of Cancer (NPC) Study, and SELECT study for genetic and nutrient based stratified analyses towards prostate cancer incidence outcomes.

21. Generalizability

Generalizability (external validity) of the trial findings, taking into account the study population, the characteristics of the intervention, length of follow-up, incentives, compliance rates, specific sites/settings involved in the study, and other contextual issues

The dietary Se intake recorded for men from this study cohort and the levels recorded from the New Zealand dietary survey (2008/2009) [(32)] are similar. It is possible that New Zealand men in general carry similar serum Se levels to the current study cohort. However, plasma Se levels recorded for a cohort of men and women (ages 19-59y) from Otago, New Zealand in early 1990s was between 62-68ng/ml [(34)]. Serum Se levels recorded for a cohort of 42 men from Auckland, New Zealand, with a high-risk for prostate cancer but were with negative biopsies for prostate cancer ranged from 59–128 ng/ml with a mean of 97.8 6 ng/ml [(28)]. Our comparisons of serum Se levels between New Zealand men with prostate cancer or benign Urology disease compared to healthy men from the current Se supplementation study with comparable ages were 101.2ng/ml, 100.7ng/ml and 112.9ng/ml respectively [(19)]. It is a possibility that the adjustments of serum Se levels to reach the range between 116-149ng/ml serum could be beneficial at least for New Zealand Caucasian men carrying the prostate cancer risk genotypes. As the current Se supplementation study duration was limited to six months, it is not easy to generalize that long term Se supplementation can have similar impacts. As this Caucasian study cohort is from New Zealand, which is a Se deficient region, it is questionable, whether the current findings can be applied to other Caucasian men from different geographical regions of the world.

22. Overall Evidence

General interpretation of the results in the context of current evidence and current theory

The well-known NPC and the SELECT studies have previously shown contradictory results of Se supplementation benefits on prostate cancer prevention. As the PSA reduction benefits (indicating prostate glandular architecture stability) in particular were realised by men carrying prostate cancer risk genotypes in the current study, it is worth checking such impacts in larger cohorts including the cohorts from the NPC and the SELECT studies that supplemented 200µg/d Se for longer durations than six months.

References

1. Fairweather-Tait SJ, Bao Y, Broadley MR *et al.* (2011) Selenium in human health and disease. *Antioxid Redox Signal* **14**, 1337-1383.
2. Ferguson LR, Karunasinghe N, Zhu S *et al.* (2012) Selenium and its' role in the maintenance of genomic stability. *Mutat Res* **733**, 100-110.
3. Rayman MP (2012) Selenium and human health. *Lancet* **379(9822)**, 1256-1268.
4. Clark LC, Combs GF, Jr., Turnbull BW *et al.* (1996) Effects of selenium supplementation for cancer prevention in patients with carcinoma of the skin. A randomized controlled trial. Nutritional Prevention of Cancer Study Group. *JAMA* **276**, 1957-1963.
5. Lippman SM, Klein EA, Goodman PJ *et al.* (2009) Effect of selenium and vitamin E on risk of prostate cancer and other cancers: the Selenium and Vitamin E Cancer Prevention Trial (SELECT). *JAMA* **301**, 39-51.
6. Vinceti M, Filippini T, Rothman KJ (2018) Selenium exposure and the risk of type 2 diabetes: a systematic review and meta-analysis. *Eur J Epidemiol* **33**, 789-810.

7. Vinceti M, Filippini T, Del Giovane C *et al.* (2018) Selenium for preventing cancer. *Cochrane Database Syst Rev* **1**, CD005195.
8. Speckmann B, Schulz S, Hiller F *et al.* (2017) Selenium increases hepatic DNA methylation and modulates one-carbon metabolism in the liver of mice. *J Nutr Biochem* **48**, 112-119.
9. Ferguson LR, Karunasinghe N, Zhu S *et al.* (2012) Understanding Heterogeneity in Supplementation Effects of Selenium in Men: A Study of Stratification Variables and Human Genetics in a Prospective Sample from New Zealand. *Current Pharmacogenomics and Personalized Medicine* **10**, 204-216.
10. Karunasinghe N, Han DY, Zhu S *et al.* (2012) Serum selenium and single-nucleotide polymorphisms in genes for selenoproteins: relationship to markers of oxidative stress in men from Auckland, New Zealand. *Genes nutr* **7**, 179-190.
11. Speckmann B, Grune T (2015) Epigenetic effects of selenium and their implications for health. *Epigenetics* **10**, 179-190.
12. Ferguson LR, Karunasinghe N, Philpott M (2004) Epigenetic events and protection from colon cancer in New Zealand. *Environ Mol Mutagen* **44**, 36-43.
13. Narayan V, Ravindra KC, Liao C *et al.* (2015) Epigenetic regulation of inflammatory gene expression in macrophages by selenium. *J Nutr Biochem* **26**, 138-145.
14. Sun W, Wang Q, Guo Y *et al.* (2017) Selenium suppresses inflammation by inducing microRNA-146a in Staphylococcus aureus-infected mouse mastitis model. *Oncotarget* **8**, 110949-110964.
15. Kohler LN, Foote J, Kelley CP *et al.* (2018) Selenium and Type 2 Diabetes: Systematic Review. *Nutrients* **10**.
16. Sinha I, Karagoz K, Fogle RL *et al.* (2016) "Omics" of Selenium Biology: A Prospective Study of Plasma Proteome Network Before and After Selenized-Yeast Supplementation in Healthy Men. *OMICS* **20**, 202-213.
17. Hawkes WC, Richter BD, Alkan Z *et al.* (2008) Response of selenium status indicators to supplementation of healthy North American men with high-selenium yeast. *Biol Trace Elem Res* **122**, 107-121.
18. Karunasinghe N, Han DY, Zhu S *et al.* (2013) Effects of supplementation with selenium, as selenized yeast, in a healthy male population from New Zealand. *Nutr Cancer* **65**, 355-366.
19. Karunasinghe N, Han DY, Goudie M *et al.* (2013) Prostate Disease Risk Factors among a New Zealand Cohort. *J Nutrigenet Nutrigenomics* **5**, 339-351.
20. Vaidyanathan V, Naidu V, Kao CH-J *et al.* (2017) Environmental factors and risk of aggressive prostate cancer among a population of New Zealand men - a genotypic approach. *Molecular BioSystems 2017*.
21. Prcic A, Begic E, Hiros M (2016) Usefulness of Total PSA Value in Prostate Diseases Diagnosis. *Acta Inform Med* **24**, 156-161.
22. Ministry-of-Health-New-Zealand (2018) Mortality: Historical summary 1948–2015. https://www.health.govt.nz/publication/mortality-historical-summary-1948-2015
23. Ferguson LR, Han DY, Fraser AG *et al.* (2010) Genetic factors in chronic inflammation: single nucleotide polymorphisms in the STAT-JAK pathway, susceptibility to DNA damage and Crohn's disease in a New Zealand population. *Mutat Res* **690**, 108-115.
24. Karunasinghe N, Ryan J, Tuckey J *et al.* (2004) DNA stability and serum selenium levels in a high-risk group for prostate cancer. *Can Epid Biom & Prev* **13**, 391-397.
25. Watkinson JH (1979) Semi-Automated Fluorimetric Determination of Nanogram Quantities of Selenium in Biological Materials. *Anal Chim Acta* **105**, 319-325.
26. Watkinson JH, Brown MW (1979) New Phase Separating Device and Other Improvements in the Semi-Automated Fluorimetric Determination of Selenium. *Anal Chim Acta* **105**, 451-454.
27. Rongpu Y, Jiachen H, Gongkan F *et al.* (1986) Fluorometric determination of micro-amounts of selenium in human blood, using 2,3-diaminonaphthalene. *Med Lab Sci* **43**, 331-334.
28. Karunasinghe N, Ferguson LR, Tuckey J *et al.* (2006) Hemolysate thioredoxin reductase and glutathione peroxidase activities correlate with serum selenium in a group of New Zealand men at high prostate cancer risk. *J Nutr* **136**, 2232-2235.
29. Gill CI, Haldar S, Porter S *et al.* (2004) The effect of cruciferous and leguminous sprouts on genotoxicity, in

vitro and in vivo. *Cancer Epidemiol Biomarkers Prev* **13**, 1199-1205.
30. Rodriguez S, Gaunt TR and Day IN. (2009) Hardy-Weinberg Equilibrium Testing of Biological Ascertainment for Mendelian Randomization Studies.
http://www.oege.org/software/hwe-mr-calc.shtml
31. National-Health-and-Medical-Research-Council-(Australia)-and-Ministry-of-Health-New-Zealand (2019) *Nutrient Reference Values for Australia and New Zealand*.
32. University-of-Otago-and-Ministry-of-Health (2011) A Focus on Nutrition: Key Findings of the 2008/09 New Zealand Adult Nutrition Survey. https://www.health.govt.nz/system/files/documents/publications/a-focus-on-nutrition-ch4_0.pdf
33. Vogt TM, Ziegler RG, Graubard BI *et al.* (2003) Serum selenium and risk of prostate cancer in U.S. blacks and whites. *Int J Cancer* **103**, 664-670.
34. Duffield AJ, Thomson CD, Hill KE *et al.* (1999) An estimation of selenium requirements for New Zealanders. *Am J Clin Nutr* **70**, 896-903.
