# Supplementary material for: Selenium Supplementation and Prostate Health in a New Zealand Cohort
Source: Nutrients. 2019 Dec 18;12(1):2. doi: 10.3390/nu12010002 (PMC7019779; doi:10.3390/nu12010002)
Supplement: Supplementary file 1 [file nutrients-12-00002-s001.zip › nutrients-609717-supplementary document 2.pdf]

Supplementary document 2-

GENERAL RULES FOR FOODWORKS DATA ENTRY

Many items listed in the diaries will lack detail in terms of the **food item itself** and the **quantity**. Use the table compiled to help you determine details that you are uncertain of.

- How To Use The Table:
  - Simply use Ctrl + F or search manually (the table is alphabetised) to find the item in question (e.g. “chips”) and a default Foodworks Food Item and Foodworks Quantity will be listed.
- The “Once Mentioned” Rule:
  - If, during the first day of the diary, they mention a certain food item but they thereafter do not mention this item in the remaining days assume that this item has been taken
  - Example 1 – They state “milk” with muesli on day 1 but then on days 2-5 only state muesli, assume milk has been taken on days 2-5.
  - Example 2 – They state “vogels bread” for a sandwich on day 1 but then on days 2-5 only state “bread”, assume “vogels” has been consumed on days 2-5.
  - Example 3 – They state “butter” for frying an egg on day 1 but then on a subsequent meal do not mention what “Steak 200g” was fried in, assume butter (1 Tb) was used.
- Constructing a “sandwich”
  - Search for bread that best correlates and use 2 slices
  - Search for individual ingredients (e.g. “Onion”) in database. Select the “Onion (In Sandwich)” listing and quantity provided
  - Assume 10g of margarine/butter used unless otherwise stated
- Fat used for cooking meat:
  - If butter has been used at another point in diary, assume they have also cooked meat in “Butter, salted”
  - If butter has not been specified anywhere in diary, assume they have cooked meat in “Vegetable oil”
