# Supplementary material for: Selenium Supplementation and Prostate Health in a New Zealand Cohort
Source: Nutrients. 2019 Dec 18;12(1):2. doi: 10.3390/nu12010002 (PMC7019779; doi:10.3390/nu12010002)
Supplement: Supplementary file 1 [file nutrients-12-00002-s001.zip › nutrients-649717-supplementary tabel S1 and S2.pdf]

# SUPPLEMENTARY TABLE 1- FOR UNSPECIFIED FOOD ITEMS AND QUANTITIES

FOR FOODWORKS 2009 PROFESSIONAL EDITION

| FOOD ITEM | DEFAULT INPUT | DEFAULT QUANTITIES |
|---|---|---|
| Bacon | "Pork, bacon, lean&fat, uncooked" + "Butter, salted"/"Vegetable Oil" | 3 Rashers + 1 Tb Butter/Oil |
| Baked Beans | "Beans, baked, in tomato sauce, canned" | 100g w toast; 200g w/o |
| Beef (In Sandwich) | "beef, bolar roast, lean&fat, trm, roasted" | 1 slice |
| Beer | "Beer, standard, draught&lager" | Can - 330ml; Glass - 300ml; Pint 500ml; Jug 1000ml |
| Biscuit (Chocolate) (unspecified) | "Biscuit, chocolate coated" | 2 biscuits |
| Biscuit (unspecified) | "Biscuit, basic, NZ recipe" | 2 biscuits |
| Bourbon | "Whisky" | 50ml/50g |
| Bread (Bun/Roll) (Unspecified) | "Bread roll, white, pre-packaged" | 50g |
| Bread (Rye - Toasted) | "Bread, rye, heavy, toasted" | 2 Slices |
| Bread (Vogels) | "Vogels Sunflower and Barley" | 60g (2 Slices) |
| Bread (Vogels - Toasted) | "Vogels Toast Soy and Linseed Toast" | 60g (2 Slices) |
| Bread (Wheatmeal) | "Bread, wheatmeal, sliced, prepacked" | 2 Slices |
| Bread (Wholemeal) | "Bread, wholemeal, sliced&unsliced" | 2 Medium Slices |
| Butter (In Sandwich) | "Butter, semi-soft" | 10g per Sandwich |
| Cake (unspecified) | "Cake, fancy, iced" | 1 slice |
| Carrot (In Sandwich) | "Carrot, flesh, raw" | 0.25 Cup, Sliced |
| Cassava | "Cassava, boiled" | 150g |
| Cheese | "Cheese, cheddar, mild" | 40g |
| Cheese (In Sandwich) | "Cheese, cheddar, mild" | 40g; 80g for Toasted Sandwich |
| Chicken (unspecified) | "Chicken, breast, lean&fat, roasted" + "Butter, salted"/"Vegetable Oil" | 1 single breast (chicken) + 10g Butter/Oil |
| Chips (i.e. "packet" chips) | "Potato crisps, plain" | 1 small packet |
| Chips (i.e. Fish and Chips) | "Potato, fries, independent shops, plain cut" | 1 "average serve" |
| Chocolate (unspecified) | "Chocolate bar, milk" | 1 small bar |
| Coffee (Unspecified) | "Coffee, instant, powder" + "Water, municipal" + "Milk, fluid, standard" | 1 Cup - 1 tsp Coffee Powder + 250ml Water + 20ml Standard Milk |
| Cornflakes | "Corn flakes" | 50g |
| Croissant | "Croissant" | Small (7 to 12 cm long) |
| Crumble (Apple/Fruit) | "Pudding, apple crumble, baked" | 100g |
| Curry | "Curry, beef/chicken/fish/lamb/vegetarian with gravy, stewed" + "Rice, white, polished, boiled" | 200g curry + 1 cup rice |

| | | |
|---|---|---|
| Eggs (scrambled) | "Eggs, scrambled, w/milk in butter" | 2 eggs |
| Energy Drink | "Fruit Drink, V Drink" | 1 Can (250ml) |
| Fish (Tuna - flavoured) | "Tuna, in savoury sauce, canned" | 100g |
| Fish (Tuna - Lite) | "Tuna, canned in brine, drained" | 100g |
| Fish (Tuna - unspecified) | "Tuna, canned in oil" | 100g |
| Fruit Juice | "Juice, orange&apple, unsw, Just Juice" | 250ml |
| Fruit Salad | "Fruit salad, fruit&syrup, canned" | 120g |
| Ham (In Sandwich) | "Ham, sliced, sandwich" | 1 slice |
| Ham (Steak) | "Pork, ham steak, lean&fat, grilled" | 1 Steak |
| HP Sauce | "Sauce, barbecue" | 20ml |
| Ice Cream | "Ice cream, vanilla, standard" | 140g |
| Jam (unspecified) | "Jam, artificially sweetened" | 10g |
| Lettuce (In Sandwich) | "Lettuce, inner&outer leaves, raw" | 1 medium leaf |
| Margarine (In Sandwich) | "Margarine, spread, Miracle" | 10g |
| Marmalade (unspecified) | "Marmalade, artificially sweetened" | 10g |
| Milk | "Milk, Standard" | 250ml |
| Milkshake | "Milkshake, assorted flavour, McDonalds" | 450ml |
| Mince (unspecified) | "Beef, mince, lean&fat(12%), stewed" | 195g |
| Mixed Vegetables | "Vegetables, 3 mixed, frozen, boiled, drained" | 60g |
| Muesli | "Muesli, natural, sanitarium" + "Milk, fluid, standard" | 55g Muesli + 0.5 Cup Milk |
| Muesli Bar (Chocolate-Coated) | "Nice&Natural Nut Bar - Chocolate" | 30g |
| Muffin | "Muffin, assorted flavours, toasted" | 1 muffin |
| Onion (cooked) | "Onion, flesh, fried in dripping" | 100g |
| Onion (In Sandwich) | "Onion, flesh, raw" | 0.25 Cup, Chopped |
| Onion (raw) | "Onion, flesh, raw" | 1 "onion" |
| Pasta | "Pasta, plain, boiled" | 150g |
| Peanut Butter (unspecified) | "Peanut Butter, smth&crh, sugar&salt added" | 10g |
| Pepperoni (In Sandwich) | "Salami, uncooked" | 4 slices |
| Peppers (In Sandwich) | "Pepper, sweet, red, raw" | 0.25 Cup |

| | | |
|---|---|---|
| Pizza Bread | "Pizza, cheese&tomato" | 1 individual lunch-time pan pizza |
| Porridge (cooked) | "Porridge, prepared with milk" | 130g |
| Potato (unspecified) | "Potato, rua, boiled, salt added" | 1 potato |
| Pumpkin (unspecified) | "Pumpkin, flesh&skin, roasted" | 135g |
| Rice (unspecified) | "Rice, white, polished, boiled" | 150g |
| Salad (unspecified) | "Salad, lettuce without cheese" + "Dressing, Salad, Kraft" | 2 Cups Salad + 1 Tb Dressing |
| Salami (In Sandwich) | "Salami, uncooked" | 4 Slices |
| Sandwich - Club | "Sandwich, white bread, ham/bacon&veg" | 1 sandwich |
| Sausage (unspecified) | "Sausage, preckd, dry fried, asst meats&flvr" | 2 sausage |
| Soup | "Soup, bone&vegetable broth" | Standard - 250ml; Large - 500ml |
| Spaghetti (Canned) | "Spaghetti in tomato sauce, canned" | 100g w toast; 200g w/o |
| Steak (unspecified) | "Beef, rump steak, lean(85%)&fat,raw" + "Butter, salted"/"Vegetable Oil" | Small - 1x Steak, Medium - 1.5 Steak, Large - 2 Steak + 1 Tb Butter/Oil |
| Stewed Fruit | "(Fruit), cooking, with sugar, stewed " | 135g |
| Stir-fry (with meat) | "Beef/Chicken/Lamb stir-fried with vegetables" | 200g |
| Tapioca | "Tapioca, raw" | 150g |
| Tea | "Tea, black, infused, weak" | 1 Cup - 250ml Tea + 20ml Standard Milk |
| Tomato | "Tomato, flesh, skin&seeds, raw" | 1 tomato |
| Tomato (In Sandwich) | "Tomato, flesh, skin&seeds, raw" | 0.5 Tomato |
| Whisky | "Whisky" | Nip - 50ml/50g; 1 Shot - 25ml/25g |
| Wine (Brut) | "Wine, white, dry" | Glass - 150ml (NB - Foodworks "Glass" is equivalent to a small glass) |
| Wine (Chardonnay) | "Wine, white, medium" | Glass - 150ml (NB - Foodworks "Glass" is equivalent to a small glass) |
| Wine (Gewutz) | "Wine, white, sweet" | Glass - 150ml (NB - Foodworks "Glass" is equivalent to a small glass) |
| Wine (Pinot Gris) | "Wine, white, medium" | Glass - 150ml (NB - Foodworks "Glass" is equivalent to a small glass) |
| Wine (Reisling) | "Wine, white, sweet" | Glass - 150ml (NB - Foodworks "Glass" is equivalent to a small glass) |
| Wine (Sauvignon Blanc) | "Wine, white, medium" | Glass - 150ml (NB - Foodworks "Glass" is equivalent to a small glass) |
| Wine (Unspecified) | "Wine, white, medium" | Glass - 150ml (NB - Foodworks "Glass" is equivalent to a small glass) |
| Yoghurt (homemade) | "Yoghurt, plain, unsweetened" | 150g |
| Yoghurt (unspecified) | "Yoghurt, asst fruit&flavours, sweetened" | 150g |

**Supplementary Table 2**

| Join age | BMI | Ever smok | Achohol c | health disorder | tal-folate_ | methionine_g | zinc_mg | b6-(by-analy | itamin-b12_µ | elenium_µ | -as-mono_ | t-as-poly_ | s-saturate | om-protein | -carbohyd | Selenium | Selenium | PSA V1 | PSA V2 | s1763254 | 50450 G | AKR1C3 | 380 MnS | 845 Sep-15 |
|---|---|---|---|---|---|---|---|---|---|---|---|---|---|---|---|---|---|---|---|---|---|---|---|---|
| 50 | | FALSE | FALSE | None | | | | | | | | | | | | 110.544 | | 1.4 | | T/T | CT | CC | TC | CT |
| 24 | 30 | TRUE | TRUE | inflammatory | 707.179 | 0.5064735 | 13.9679 | 1.364967 | 11.96485 | 71.0824 | 34.0608 | 18.1307 | 47.8085 | 12.5483 | 42.3574 | 94.752 | 157.92 | 0.1 | 0.20 | T/T | TT | CG | TT | CC |
| 28 | 22 | FALSE | TRUE | None | 593.022 | | 15.0419 | | 3.395564 | 59.0734 | | | | 18.728 | | 94.752 | | 0.4 | | T/T | CT | CC | TC | CT |
| 41 | 22 | TRUE | TRUE | None | 714.096 | 0.9745941 | 13.6985 | 1.705585 | 3.822663 | 94.5123 | 45.8187 | 18.9699 | 35.2114 | 14.13 | 46.5864 | 126.336 | 157.92 | 0.2 | 0.30 | T/T | CC | CC | CC | CT |
| 30 | 21 | FALSE | TRUE | None | 174.675 | | 7.35938 | | 7.230097 | 97.0981 | | | | 15.4338 | | 126.336 | | 0.8 | | T/T | CT | CC | CC | CT |
| 46 | 29 | FALSE | TRUE | Cardiovascular | 471.4 | 1.078197 | 9.43776 | 1.676395 | 3.921701 | 77.5575 | 42.299 | 15.0069 | 42.694 | 18.3239 | 45.6373 | 157.92 | 142.128 | 0.2 | 0.10 | T/T | CT | GG | TC | CC |
| 52 | 35 | FALSE | TRUE | None | 289.416 | 0.7218666 | 14.8136 | 2.981186 | 3.387705 | 41.7692 | 38.8298 | 18.8217 | 42.3485 | 16.8274 | 46.1798 | 118.44 | 157.92 | 0.7 | 1.10 | T/T | CT | CC | TC | CT |
| 50 | 26 | FALSE | TRUE | Cardiovascular | 346.524 | 0.7738495 | 12.1932 | 1.176987 | 42.57822 | 43.5156 | 42.9549 | 22.9293 | 34.1158 | 16.1383 | 43.6472 | 110.544 | 181.608 | 2.7 | 2.30 | T/T | CC | CC | TT | CC |
| 55 | 29 | FALSE | TRUE | None | 247.946 | | 11.7299 | | 41.21092 | 81.6848 | | | | 17.2222 | | 118.44 | | 0.3 | | T/T | CT | CG | TC | CC |
| 30 | 24 | FALSE | TRUE | None | | | | | | | | | | | | 118.44 | | 0.3 | | T/T | CT | CG | CC | TT |
| 46 | 21 | FALSE | FALSE | None | 775.594 | 0.6102524 | 12.7619 | 3.661098 | 6.426373 | 118.01 | 41.7851 | 21.8689 | 36.3461 | 20.8221 | 50.1869 | 118.44 | 181.608 | 1.3 | 2.20 | T/T | CC | CC | TC | CC |
| 35 | 28 | TRUE | TRUE | None | 554.506 | 0.3529816 | 15.9087 | 1.485298 | 8.036102 | 79.6377 | 39.8411 | 23.4397 | 36.7192 | 15.6805 | 30.3528 | 150.024 | 173.712 | 1 | 1.10 | T/T | CC | CG | TC | CC |
| 69 | 28 | FALSE | TRUE | None | 276.764 | 0.7889023 | 11.1107 | 2.934185 | 40.60554 | 44.1832 | 38.2057 | 15.7954 | 45.999 | 14.5175 | 44.4011 | 126.336 | 157.92 | 1.4 | 0.80 | C/T | CT | GG | TC | CC |
| 51 | 26 | TRUE | FALSE | None | 227.922 | | 10.8892 | | 3.371837 | 45.9274 | | | | 15.8311 | | 94.752 | 150.024 | 0.8 | 0.80 | T/T | CC | CG | TC | CC |
| 67 | 25 | FALSE | FALSE | None | 614.814 | 0.7788239 | 12.1726 | 1.341946 | 3.401674 | 28.374 | 42.5083 | 15.988 | 41.5037 | 12.6325 | 46.9822 | 110.544 | 173.712 | 3.6 | 3.60 | T/T | CC | GG | CC | CC |
| 56 | 27 | FALSE | TRUE | Cardiovascular | | | | | | | | | | | | 126.336 | | 0.5 | | T/T | CT | CC | CC | CC |
| 59 | 27 | TRUE | TRUE | None | | | | | | | | | | | | 142.128 | 197.4 | 0.6 | 0.70 | T/T | CT | CG | CC | CT |
| 62 | 27 | FALSE | TRUE | None | 419.839 | 0.3980751 | 17.3248 | 1.207527 | 4.45488 | 54.3188 | 37.7434 | 19.967 | 42.2896 | 15.3294 | 41.5767 | 118.44 | 189.504 | 1.9 | 2.30 | T/T | CT | CG | TC | CT |
| 46 | 21 | TRUE | TRUE | None | 458.239 | 1.197166 | 14.9432 | 2.181595 | 11.98589 | 72.3698 | 45.0205 | 20.6097 | 34.3698 | 14.3541 | 43.6778 | 118.44 | 173.712 | 0.6 | 0.40 | T/T | CC | CC | TT | CC |
| 61 | 24 | TRUE | TRUE | None | 1056.64 | 0.9511986 | 27.6511 | 2.700237 | 32.43908 | 124.714 | 35.4598 | 19.5831 | 44.9571 | 17.018 | 50.8865 | 134.232 | 157.92 | 1.4 | 1.40 | T/T | CC | CC | TC | CC |
| 39 | 26 | FALSE | TRUE | None | 311.449 | | 13.629 | | 4.058841 | 39.9164 | | | | 19.4113 | | 110.544 | | 1.1 | | T/T | CT | CG | TC | CC |
| 25 | 26 | FALSE | TRUE | None | 536.555 | | 13.5376 | | 6.204854 | 190.574 | | | | 27.6568 | | 134.232 | | 0.5 | | T/T | CT | GG | TT | CC |
| 28 | 26 | FALSE | TRUE | None | 187.227 | | 10.147 | | 41.35888 | 61.5068 | | | | 19.9011 | | 142.128 | | 0.6 | | T/T | CC | CG | CC | CT |
| 63 | 30 | FALSE | TRUE | Cardiovascular | 134.46 | 0.9336243 | 6.8544 | 1.104828 | 4.74638 | 122.316 | 38.2373 | 10.7396 | 51.0231 | 17.3182 | 27.596 | 110.544 | 134.232 | 0.5 | 0.40 | T/T | CT | CC | TC | CC |
| 24 | 24 | FALSE | FALSE | None | 448.347 | 1.566055 | 21.6871 | 3.453556 | 11.11286 | 60.9241 | 37.5364 | 14.2971 | 48.1665 | 16.3218 | 37.0819 | 102.648 | 126.336 | 0.6 | 0.80 | T/T | CC | GG | TC | CC |
| 57 | 32 | FALSE | TRUE | None | | | | | | | | | | | | 102.648 | 142.128 | | 0.80 | T/T | TT | CC | TC | CC |
| 44 | 25 | FALSE | TRUE | None | | | | | | | | | | | | 94.752 | 142.128 | 0.5 | 0.50 | C/T | CT | CG | TT | CC |
| 43 | 24 | FALSE | TRUE | inflammatory | | | | | | | | | | | | 110.544 | 181.608 | 0.5 | 0.60 | T/T | TT | CC | TC | CC |
| 24 | 21 | FALSE | TRUE | None | | | | | | | | | | | | 110.544 | 165.816 | 0.4 | 0.50 | T/T | CT | CG | TT | CC |
| 32 | 23 | TRUE | TRUE | None | 316.377 | 0.4321671 | 14.6817 | 5.467346 | 19.41564 | 67.6243 | 42.7922 | 23.3932 | 33.8146 | 14.9719 | 34.0243 | 102.648 | 177.66 | 0.3 | 0.30 | T/T | CT | CC | CC | CC |
| 64 | 30 | FALSE | TRUE | Cardiovascular | 227.026 | | 12.8286 | | 2.331576 | 50.557 | | | | 19.0869 | | 102.648 | | 3.2 | | T/T | CC | CC | TC | CT |
| 54 | 26 | TRUE | FALSE | None | | | | | | | | | | | | 102.648 | | 3.4 | | T/T | CC | CC | TT | CC |
| 31 | 29 | FALSE | TRUE | None | 597.827 | 1.053478 | 14.9144 | 2.073185 | 5.367338 | 108.635 | 35.4731 | 20.6053 | 43.9217 | 22.4957 | 46.9716 | 102.648 | 150.024 | 0.6 | 0.40 | T/T | CC | CG | TC | CC |
| 56 | 28 | FALSE | TRUE | other | | | | | | | | | | | | | | | | C/T | CT | CG | TT | CT |
| 27 | 23 | FALSE | TRUE | None | 131.607 | 1.521923 | 10.9174 | 1.166252 | 3.569063 | 37.021 | 42.1046 | 10.668 | 47.2274 | 19.1769 | 42.4392 | 126.336 | 146.076 | 0.6 | 0.60 | T/T | CT | GG | TC | CC |
| 22 | 0 | TRUE | TRUE | None | | | | | | | | | | | | 126.336 | 161.868 | 0.9 | 0.80 | T/T | CT | CG | TC | CC |
| 34 | 23 | FALSE | TRUE | None | 345.397 | | 13.9352 | | 6.469574 | 92.7779 | | | | 14.4388 | | 110.544 | 150.024 | 0.6 | 0.60 | T/T | CT | CC | TT | CT |
| 27 | 23 | FALSE | TRUE | None | | | | | | | | | | | | 126.336 | | 0.6 | | T/T | CC | CG | CC | CC |
| 56 | 26 | TRUE | TRUE | None | | | | | | | | | | | | 126.336 | | 0.8 | | T/T | CC | GG | TT | CT |
| 42 | 26 | FALSE | TRUE | None | 586.756 | 0.8983192 | 16.1709 | 1.418648 | 5.361638 | 44.5973 | 38.0532 | 14.1448 | 47.802 | 15.4296 | 47.3089 | 126.336 | 150.024 | 0.9 | 0.90 | T/T | CT | GG | TC | CC |
| 55 | 30 | FALSE | TRUE | Cardiovascular | | | | | | | | | | | | 110.544 | 173.712 | | 0.40 | | CC | CC | TC | CC |
| 49 | 27 | FALSE | TRUE | Cardiovascular | | | | | | | | | | | | 110.544 | 165.816 | 1.6 | 1.40 | T/T | CC | CC | CC | CC |
| 39 | 23 | FALSE | TRUE | None | | | | | | | | | | | | 110.544 | 236.88 | | 0.20 | T/T | CC | CC | CC | CT |
| 45 | 27 | FALSE | TRUE | None | 271.192 | | 14.069 | | 5.267303 | 27.9575 | | | | 14.2941 | | 110.544 | 110.544 | | 1.10 | T/T | CT | CC | CC | CT |
| 28 | 23 | FALSE | TRUE | inflammatory | | | | | | | | | | | | 134.232 | | 0.9 | | T/T | CT | CC | TC | CC |
| 45 | 26 | FALSE | TRUE | None | 225.951 | 0.8865128 | 10.0775 | 1.317734 | 4.267712 | 101.328 | 37.5702 | 9.00125 | 53.4285 | 15.7506 | 39.5386 | 102.648 | 150.024 | 0.7 | 0.70 | T/T | CC | CC | TC | CC |
| 48 | 21 | FALSE | TRUE | None | 273.423 | 0.8391609 | 12.5228 | 0.808308 | 4.788295 | 104.974 | 39.8793 | 13.4881 | 46.6326 | 16.2292 | 41.3919 | 102.648 | 165.816 | 0.7 | 0.60 | T/T | CC | CC | TC | CT |
| 51 | 24 | TRUE | TRUE | None | 363.543 | 0.8192139 | 21.8376 | 1.837952 | 9.592262 | 130.781 | 40.9014 | 17.8883 | 41.2104 | 20.8753 | 32.9591 | 134.232 | 157.92 | 0.3 | 0.40 | T/T | CT | CC | TT | CT |
| 28 | 31 | FALSE | FALSE | None | 209.051 | 0.7357521 | 10.1031 | 0.98444 | 4.509002 | 72.9854 | 37.3651 | 24.179 | 38.4559 | 32.6437 | 30.5812 | 126.336 | 150.024 | 0.3 | 0.20 | C/T | CT | CG | TT | CT |
| 65 | 29 | TRUE | TRUE | None | 457.483 | 0.7421188 | 11.3657 | 0.821419 | 23.45918 | 147.452 | 37.3918 | 16.209 | 46.3992 | 19.049 | 37.1263 | 134.232 | 181.608 | 0.5 | 0.50 | C/T | CC | CG | CC | CT |
| 43 | 24 | TRUE | TRUE | None | | | | | | | | | | | | 126.336 | 189.504 | 1.1 | 1.10 | T/T | CT | CG | TC | CT |
| 55 | 26 | FALSE | TRUE | None | | | | | | | | | | | | 126.336 | 138.18 | 0.7 | 0.70 | T/T | CC | CC | TT | CT |
| 42 | 23 | TRUE | TRUE | None | 227.293 | 0.9475021 | 15.6026 | 1.495575 | 8.226512 | 64.4661 | 40.3718 | 13.04 | 46.5883 | 15.8136 | 52.4251 | 134.232 | 181.608 | 0.5 | 0.40 | T/T | CT | CC | TC | CT |
| 45 | 37 | FALSE | TRUE | None | 256.017 | 0.5183411 | 7.01845 | 0.835751 | 3.505766 | 131.088 | 40.6815 | 28.4893 | 30.8292 | 20.6311 | 37.5931 | 110.544 | 173.712 | 0.4 | 0.40 | T/T | TT | CC | TC | CT |
| 46 | 22 | FALSE | TRUE | None | 769.268 | 1.056255 | 37.109 | 3.976418 | 3.407006 | 62.9136 | 44.9363 | 20.837 | 34.2267 | 13.5586 | 37.1189 | 118.44 | 138.18 | 0.5 | 0.50 | T/T | CT | GG | CC | CC |
| 53 | 19 | FALSE | FALSE | None | | | | | | | | | | | | 78.96 | | 0.9 | | T/T | CT | CC | TT | CC |
| 51 | 27 | TRUE | TRUE | None | 343.856 | 0.9030266 | 11.3924 | 0.949081 | 2.701235 | 44.2382 | 40.065 | 21.6994 | 38.2356 | 14.6371 | 35.844 | 118.44 | 157.92 | 1 | 1.20 | T/T | CC | CG | CC | CC |
| 43 | 27 | FALSE | TRUE | None | 1002.63 | 0.8472366 | 49.2366 | 1.377586 | 54.89246 | 225.825 | 38.3707 | 12.0615 | 49.5679 | 21.8247 | 49.5469 | 150.024 | 185.556 | 1 | 0.80 | C/T | CC | CC | TC | CC |
| 61 | 32 | TRUE | TRUE | other | 817.258 | 1.211563 | 15.7607 | 1.13205 | 4.464501 | 77.9563 | 49.1393 | 18.7942 | 32.0666 | 15.7386 | 33.17 | 94.752 | 150.024 | 0.7 | 0.70 | T/T | CT | CC | TT | CT |
| 39 | 25 | TRUE | TRUE | None | 504.637 | 0.5035667 | 10.6378 | 1.199356 | 7.801831 | 71.3073 | 40.3619 | 20.8048 | 38.8333 | 13.1996 | 38.7135 | 134.232 | 221.088 | 0.3 | 0.20 | T/T | CC | CG | TC | CC |
| 51 | 28 | TRUE | TRUE | Cardiovascular | 394.205 | 1.411102 | 17.8228 | 2.373234 | 5.523082 | 44.855 | 35.2661 | 12.5809 | 52.1531 | 17.5483 | 41.8772 | 118.44 | 173.712 | 0.5 | 0.60 | T/T | CT | CG | TC | CC |
| 51 | 25 | TRUE | TRUE | inflammatory | 423.833 | 0.7487869 | 13.0112 | 1.154076 | 5.408451 | 62.7379 | 41.5437 | 24.9588 | 33.4975 | 15.0186 | 36.4937 | 157.92 | 189.504 | 0.8 | 1.40 | T/T | CT | GG | CC | CC |
| 54 | 28 | FALSE | TRUE | None | 387.577 | 0.6651726 | 18.3562 | 2.237404 | 6.793123 | 77.6317 | 34.2404 | 19.0009 | 46.7587 | 13.9695 | 34.5743 | 110.544 | 189.504 | 0.2 | 0.30 | T/T | CC | CC | TT | CT |
| 43 | 26 | FALSE | TRUE | None | | | | | | | | | | | | 134.232 | 189.504 | 1.1 | 1.00 | T/T | CT | CG | TC | CC |
| 59 | 23 | TRUE | TRUE | inflammatory | | | | | | | | | | | | 142.128 | | 1.5 | | T/T | CT | CC | TC | CC |
| 24 | 24 | FALSE | TRUE | None | 199.383 | 0.9323311 | 11.4524 | 1.438869 | 2.489563 | 78.8968 | 37.5128 | 20.8196 | 41.6676 | 14.433 | 40.6413 | 102.648 | 165.816 | 0.3 | 0.20 | C/T | CC | CC | TC | CC |
| 60 | 24 | FALSE | FALSE | None | 609.343 | 0.896492 | 14.2065 | 0.9664 | 3.422075 | 45.0154 | 35.4812 | 12.1697 | 52.3491 | 13.7422 | 51.1134 | 110.544 | 189.504 | 2.3 | 2.00 | T/T | CC | CG | TT | CT |
| 57 | 35 | FALSE | TRUE | None | 358.803 | 1.433311 | 9.07577 | 1.678829 | 3.127651 | 46.3438 | 46.2077 | 12.1859 | 41.6064 | 18.631 | 34.6618 | 134.232 | 165.816 | 0.6 | 1.30 | T/T | CT | GG | TT | CT |
| 51 | 24 | FALSE | FALSE | None | 241.617 | 1.160015 | 14.0337 | 2.60743 | 3.160535 | 42.9737 | 38.7554 | 14.3896 | 46.855 | 14.6938 | 43.4519 | 110.544 | 181.608 | 0.3 | 0.20 | T/T | CT | GG | CC | CC |
| 66 | 27 | FALSE | FALSE | inflammatory | | | | | | | | | | | | 118.44 | 173.712 | 6.5 | 6.90 | T/T | CC | CG | TT | CC |
| 52 | 24 | FALSE | TRUE | None | 294.146 | 1.171051 | 16.4681 | 2.606606 | 7.9788 | 76.2602 | 37.0466 | 15.6463 | 47.3071 | 15.4361 | 38.592 | 126.336 | 142.128 | 1.2 | 1.20 | C/T | CT | CC | TC | CC |
| 53 | 23 | TRUE | TRUE | None | 357.212 | 0.6535454 | 17.1648 | 5.226448 | 4.833122 | 58.5077 | 33.9131 | 19.6869 | 46.4 | 13.5684 | 43.058 | 102.648 | 189.504 | 0.8 | 0.80 | T/T | CC | CG | TT | CC |
| 46 | | FALSE | FALSE | None | | | | | | | | | | | | 102.648 | | | | | CC | | TT | TT |
| 56 | 33 | TRUE | TRUE | Cardiovascular | 734.832 | 0.6424637 | 11.4217 | 1.402451 | 3.24302 | 97.335 | 35.4001 | 20.8784 | 43.7216 | 18.219 | 39.4607 | 94.752 | 142.128 | 0.8 | 0.70 | T/T | CT | CC | CC | CC |
| 68 | 30 | TRUE | TRUE | Cardiovascular | 365.891 | 0.7519455 | 19.1403 | 2.068043 | 6.639256 | 66.3568 | 43.154 | 16.4622 | 40.3838 | 16.0809 | 37.9542 | 118.44 | 150.024 | 6.8 | 7.90 | T/T | CT | GG | TT | CT |
| 47 | 21 | TRUE | TRUE | inflammatory | | | | | | | | | | | | 102.648 | | 0.4 | | C/C | CT | CG | TT | CT |
| 34 | 31 | FALSE | TRUE | inflammatory | 855.904 | 0.9021034 | 13.0813 | 2.280903 | 6.042346 | 47.7903 | 32.0554 | 9.24365 | 58.7009 | 17.1659 | 41.5732 | 110.544 | 165.816 | 1.2 | 1.10 | T/T | CC | CC | TC | CC |
| 31 | 22 | FALSE | TRUE | None | | | | | | | | | | | | 102.648 | | 0.4 | | T/T | CC | CC | TT | CT |
| 62 | | FALSE | TRUE | Cardiovascular | 302.59 | | 16.6238 | | 5.629712 | 56.572 | | | | 20.1741 | | 118.44 | | 2 | | T/T | CC | CG | TC | CT |
| 54 | 27 | TRUE | TRUE | Cardiovascular | 655.028 | 0.8383408 | 15.7734 | 1.753487 | 5.496296 | 75.1259 | 37.0608 | 27.8067 | 35.1326 | 20.5381 | 42.5678 | 118.44 | 161.868 | 1.2 | 0.80 | T/T | TT | CC | TC | CC |
| 39 | 25 | FALSE | TRUE | other | 275.786 | | 10.8296 | | 3.325283 | 40.4014 | | | | 15.5057 | | 94.752 | 161.868 | 0.8 | 0.40 | T/T | CT | CC | CC | CC |
| 67 | 27 | FALSE | TRUE | inflammatory | 278.473 | 0.9302025 | 10.9444 | 1.801064 | 3.320979 | 38.6231 | 34.3987 | 19.0645 | 46.5368 | 14.3731 | 44.7531 | 86.856 | 189.504 | 0.7 | 0.90 | T/T | CT | CG | TC | CT |
| 54 | 29 | FALSE | TRUE | None | 108.183 | 0.2303162 | 21.9171 | 0.440826 | 1.060008 | 140.155 | 36.7309 | 12.5518 | 50.7173 | 11.3773 | 49.0903 | 118.44 | 165.816 | 0.5 | 0.50 | | CC | CC | TC | CC |
| 50 | 28 | FALSE | TRUE | Cardiovascular | 178.783 | 0.6187248 | 14.0091 | 0.847916 | 5.558443 | 74.1551 | 40.643 | 10.0066 | 49.3505 | 24.3959 | 28.8222 | 76.5912 | 185.556 | 1.3 | 1.50 | T/T | TT | CC | TC | CT |
| 59 | 29 | FALSE | FALSE | Cardiovascular | 257.67 | 0.3704376 | 10.0805 | 0.589191 | 2.996817 | 15.8966 | 39.0225 | 22.3232 | 38.6543 | 11.5054 | 54.858 | 86.856 | 157.92 | 1 | 1.90 | T/T | CC | CC | TC | CC |
| 28 | | FALSE | FALSE | None | 299.554 | 0.952137 | 13.9718 | 1.218857 | 6.639979 | 69.9808 | 42.1551 | 13.9047 | 43.9401 | 13.6946 | 48.7598 | 110.544 | 157.92 | 0.9 | 0.90 | T/T | CC | CC | CC | CC |
| 76 | 23 | FALSE | TRUE | Cardiovascular | 792.766 | 0.8058739 | 23.8105 | 3.667912 | 126.0231 | 144.927 | 40.3834 | 30.777 | 28.8396 | 18.3975 | 44.8753 | 110.544 | 189.504 | 1.2 | 1.00 | T/T | CC | CC | CC | CC |
| 43 | 24 | TRUE | TRUE | Depression Anixiety | | | | | | | | | | | | 94.752 | 150.024 | | 0.80 | T/T | CC | CC | CC | CC |
| 41 | 26 | FALSE | TRUE | None | 256.215 | 0.6879959 | 16.7986 | 2.011124 | 3.899142 | 61.6543 | 39.5272 | 22.9583 | 37.5145 | 18.3466 | 31.5387 | 102.648 | 157.92 | 1 | 0.90 | C/T | CC | CG | TC | CC |
| 58 | 26 | TRUE | TRUE | Cardiovascular | 198.155 | | 10.9835 | | 2.587812 | 75.0176 | | | | 14.285 | | 102.648 | 148.445 | 0.9 | | T/T | CC | GG | CC | CC |
| 75 | 26 | FALSE | FALSE | Cardiovascular | 451.657 | 1.135025 | 13.8808 | 1.230183 | 4.912794 | 24.733 | 38.136 | 13.7607 | 48.1033 | 14.61 | 38.1409 | 102.648 | 169.764 | 3.7 | 3.10 | | CT | CC | CC | CT |
| 47 | | FALSE | TRUE | None | 287.163 | 0.6915817 | 14.528 | 0.962395 | 4.816513 | 229.956 | 40.2255 | 17.1175 | 42.657 | 13.7213 | 43.5479 | 86.856 | 142.128 | 0.3 | 0.30 | T/T | CC | CG | TC | CT |
| 55 | 24 | TRUE | TRUE | None | 919.199 | 0.4495354 | 38.32 | 1.680752 | 13.13164 | 45.6853 | 46.5786 | 13.5317 | 39.8898 | 10.8553 | 38.7891 | 110.544 | 138.18 | 2.8 | 2.10 | T/T | CC | GG | TT | CT |
| 62 | 24 | TRUE | TRUE | None | 165 | 1.461426 | 11.7 | 1.956108 | 3.06 | 35.4 | 40.8072 | 28.2593 | 30.9335 | 14.8181 | 31.4057 | 110.544 | 177.66 | 5.6 | 5.00 | T/T | CC | CC | CC | CT |
| 22 | | TRUE | TRUE | None | 763.205 | | 17.2357 | | 7.539842 | 201.777 | | | | 19.1036 | | 102.648 | 150.024 | 1.3 | | T/T | CC | CG | CC | CT |
| 22 | | FALSE | TRUE | None | 184.4 | 0.6815643 | 11.5824 | 1.33741 | 3.627429 | 33.7299 | 44.3466 | 18.294 | 37.3594 | 14.8677 | 36.5535 | 86.856 | 114.492 | 0.6 | 0.40 | C/T | CC | CG | TC | CC |
| 48 | 22 | FALSE | TRUE | None | 365.98 | 0.8875656 | 9.32046 | 1.67979 | 5.152365 | 99.3588 | 49.6898 | 22.9255 | 27.3847 | 22.9137 | 31.4232 | 86.856 | 157.92 | 0.7 | 0.60 | T/T | CT | CG | TC | CC |
| 61 | 25 | TRUE | TRUE | Cardiovascular | 312.172 | 0.8387451 | 8.34357 | 1.166836 | 2.22742 | 35.5048 | 33.7231 | 13.6389 | 52.6379 | 14.8444 | 39.3184 | 94.752 | 150.024 | 2.4 | 2.20 | T/T | CT | | TC | CC |
| 54 | 25 | FALSE | TRUE | other | 553.005 | 0.531517 | 21.5154 | 1.600712 | 1.635787 | 71.2827 | 40.921 | 19.1114 | 39.9676 | 10.332 | 60.9813 | 157.92 | 197.4 | 0.8 | 0.60 | | CC | GG | TC | CC |
| 43 | 28 | FALSE | TRUE | None | 230.376 | 0.5821075 | 12.1077 | 1.912735 | 5.51187 | 32.8972 | 36.55 | 17.1261 | 46.3239 | 19.6744 | 33.9191 | 142.128 | 189.504 | 0.5 | 0.30 | T/T | CT | CG | TC | CC |
| 59 | 25 | TRUE | TRUE | None | 303.273 | 0.504158 | 11.0927 | 0.604118 | 2.729119 | 62.5756 | 41.3272 | 16.5167 | 42.1561 | 13.03 | 47.8137 | 86.856 | 169.764 | 1.2 | 0.60 | T/T | CC | CG | CC | CC |
| 48 | 25 | TRUE | TRUE | None | 455.266 | 1.652348 | 19.4967 | 1.654572 | 45.93184 | 68.5741 | 44.084 | 19.4247 | 36.4913 | 23.9744 | 23.0306 | 102.648 | 146.076 | 0.7 | 0.40 | T/T | CC | GG | CC | CT |
| 73 | 25 | TRUE | TRUE | Cardiovascular | 399.849 | 0.9265862 | 12.5673 | 1.396194 | 2.732475 | 34.1843 | 44.0823 | 13.5111 | 42.4066 | 11.2251 | 32.2855 | 102.648 | 221.088 | 11.6 | 11.30 | T/T | CC | CG | TC | CC |
| 45 | 24 | FALSE | FALSE | None | 288.239 | 0.8879166 | 6.63889 | 1.139446 | 2.517586 | 70.5022 | 34.7154 | 21.0346 | 44.2499 | 12.2344 | 47.4652 | 86.856 | 213.192 | 0.6 | 0.50 | T/T | CT | | TC | CC |
| 65 | 24 | TRUE | TRUE | Cardiovascular | 502.88 | 1.390751 | 12.7152 | 0.877449 | 4.5348 | 68.8249 | 36.0627 | 7.82201 | 56.1153 | 15.3267 | 43.7351 | 86.856 | 161.868 | 1.2 | 2.30 | T/T | CT | CC | CC | CC |
| 33 | 33 | TRUE | TRUE | Cardiovascular | | | | | | | | | | | | 78.96 | 157.92 | 0.6 | 0.40 | T/T | CC | CC | CC | CC |
| 33 | 24 | TRUE | TRUE | None | 217.764 | 0.5507126 | 13.9004 | 2.404865 | 17.53726 | 58.6861 | 37.2934 | 21.5509 | 41.1557 | 16.2303 | 35.5383 | 102.648 | 173.712 | 0.4 | 0.40 | T/T | CC | CC | TC | CC |
| 58 | 24 | FALSE | FALSE | None | 548.772 | 0.6768646 | 12.3154 | 1.006088 | 6.757489 | 166.792 | 37.3919 | 5.84232 | 56.7658 | 19.2142 | 38.6356 | 102.648 | 185.556 | 0.5 | 0.60 | C/T | CT | CG | TC | CC |
| 36 | 24 | FALSE | TRUE | inflammatory | 357.474 | 1.1376 | 19.5746 | 3.869652 | 13.31103 | 165.268 | 40.127 | 13.0578 | 46.8152 | 27.7665 | 34.7275 | 102.648 | 173.712 | 0.6 | 0.70 | C/T | CT | CG | CC | CC |
| 50 | 32 | FALSE | TRUE | other | 558.79 | 0.4787788 | 20.5154 | 1.845013 | 6.97888 | 100.795 | 42.5747 | 16.6783 | 40.747 | 15.3747 | 55.4122 | 94.752 | 165.816 | 0.6 | 0.50 | T/T | CC | CC | TT | CC |
| 57 | 25 | TRUE | TRUE | Cardiovascular | 333.962 | | 8.84639 | | 3.772991 | 83.3627 | | | | 13.6034 | | 94.752 | 138.97 | 1.4 | | C/T | TT | CG | TC | CT |
| 72 | 26 | FALSE | FALSE | Depression Anixiety | | | | | | | | | | | | 110.544 | 181.608 | 2.5 | 1.90 | T/T | CC | CC | CC | CT |
| 59 | 22 | FALSE | TRUE | None | 879.305 | 1.622952 | 32.0684 | 3.5261 | 33.0716 | 86.4378 | 36.8267 | 33.5134 | 29.66 | 22.0323 | 36.0723 | 126.336 | 165.816 | 0.5 | 0.50 | T/T | TT | CC | TC | CC |
| 52 | 26 | TRUE | TRUE | cardiovascular | 450.694 | 1.249798 | 19.6417 | 1.993858 | 5.747706 | 66.8757 | 41.6607 | 10.694 | 47.6453 | 19.0759 | 37.1946 | 86.856 | 142.128 | 0.6 | 0.60 | T/T | CC | CC | TC | CT |
| 62 | | FALSE | TRUE | Urology | | | | | | | | | | | | 94.752 | 189.504 | 5.5 | 5.80 | | CC | | CC | TT |
| 70 | 28 | FALSE | FALSE | Cardiovascular | 385.554 | 1.148022 | 24.663 | 1.359673 | 30.90445 | 123.429 | 35.5006 | 15.6029 | 48.8965 | 17.6878 | 42.3978 | 86.856 | 161.868 | 2.7 | 2.30 | T/T | CT | CG | TC | CC |
| 61 | 25 | TRUE | TRUE | None | 489.631 | 1.910973 | 26.4699 | 1.998676 | 24.91776 | 56.8993 | 38.9714 | 11.8346 | 49.194 | 16.0554 | 35.9264 | 94.752 | 181.608 | 3.8 | 2.80 | C/T | CC | CC | TC | CC |
| 36 | 26 | FALSE | TRUE | None | 737.772 | 2.125282 | 24.9952 | 2.516357 | 18.72695 | 113.422 | 44.9637 | 14.7482 | 40.2882 | 24.9985 | 23.8608 | 118.44 | 181.608 | 1.5 | 1.40 | T/T | TT | GG | TC | CT |
| 54 | | FALSE | TRUE | None | 639.205 | 0.9814873 | 11.6194 | 1.608196 | 2.55846 | 41.7025 | 35.0706 | 23.9603 | 40.9691 | 13.1612 | 44.3131 | 94.752 | 146.076 | 0.7 | 0.30 | T/T | CT | CG | TT | CC |
| 61 | 31 | TRUE | TRUE | Cardiovascular | | | | | | | | | | | | 102.648 | 189.504 | 2.6 | 2.20 | T/T | TT | CC | TC | CC |
| 74 | 22 | TRUE | TRUE | Cardiovascular | 499.423 | 0.7368126 | 9.2523 | 1.625282 | 4.662221 | 37.2058 | 50.0343 | 19.4273 | 30.5383 | 15.0315 | 33.6121 | 94.752 | 126.336 | 1.1 | 0.90 | T/T | CC | CG | CC | CC |
| 61 | 31 | TRUE | TRUE | None | 204.709 | 0.3819084 | 11.2708 | 0.677605 | 3.749856 | 156.14 | 41.5576 | 16.0827 | 42.3597 | 17.5156 | 34.7074 | 150.024 | 169.764 | 7.6 | 9.00 | | CC | CG | CC | CC |
| 76 | 25 | TRUE | FALSE | None | | | | | | | | | | | | 102.648 | 213.192 | 10.6 | 0.30 | T/T | CT | CG | CC | TT |
| 57 | 26 | FALSE | TRUE | inflammatory | 364.122 | 0.7522926 | 21.4537 | 1.44751 | 15.04991 | 70.1255 | 38.8022 | 19.4894 | 41.7084 | 16.6672 | 44.671 | 110.544 | 217.14 | 0.2 | 0.10 | T/T | CC | GG | TC | CC |
| 54 | 22 | FALSE | TRUE | None | 485.978 | 1.190674 | 15.6798 | 2.403938 | 4.988901 | 75.3936 | 38.144 | 17.7815 | 44.0745 | 16.0758 | 40.7461 | 110.544 | 153.972 | 0.6 | 0.60 | C/T | CT | CG | TC | CC |
| 57 | 32 | FALSE | FALSE | None | | | | | | | | | | | | 94.752 | 173.712 | 0.4 | 0.30 | T/T | CT | CC | TT | CC |
| 67 | | FALSE | FALSE | None | | | | | | | | | | | | 86.856 | 185.556 | 9.5 | 10.60 | T/T | CC | GG | TC | CT |
| 64 | 24 | FALSE | TRUE | Cardiovascular | 402.615 | 0.5050697 | 28.615 | 1.628304 | 20.09092 | 86.2729 | 39.7196 | 20.2784 | 40.002 | 15.2868 | 34.8343 | 94.752 | 177.66 | 4.7 | 3.70 | T/T | CT | CC | CC | CT |
| 46 | 25 | TRUE | FALSE | None | | | | | | | | | | | | 110.544 | | 0.8 | 0.70 | T/T | TT | CC | TT | CC |
| 78 | 21 | TRUE | TRUE | Cardiovascular | 340.966 | 0.4068184 | 6.89302 | 3.795464 | 7.530603 | 82.7657 | 34.6662 | 33.2082 | 32.1256 | 15.0957 | 39.1447 | 110.544 | 169.764 | 0.2 | 8.30 | T/T | CC | CC | CC | CC |
| 64 | 24 | FALSE | TRUE | Urology | 432.428 | | 14.1291 | | 13.38246 | 102.081 | | | | 21.1736 | | 94.752 | 157.92 | 7.3 | 5.30 | | CT | | TC | CC |
| 60 | 25 | FALSE | TRUE | None | 316.805 | 0.7787704 | 12.2587 | 2.253216 | 4.027308 | 197.361 | 35.344 | 36.5931 | 28.063 | 20.5356 | 45.6147 | 102.648 | 228.984 | 4.5 | 5.20 | T/T | CC | CG | TC | CT |
| 33 | 24 | FALSE | TRUE | None | 190.213 | 0.4504852 | 8.45927 | 0.855301 | 3.024704 | 63.956 | 43.003 | 13.8447 | 43.1523 | 21.1864 | 34.1066 | 110.544 | 217.14 | 0.4 | 0.60 | C/T | CT | CC | TC | CC |
| 36 | 24 | TRUE | TRUE | None | 248.876 | 0.6186066 | 9.52649 | 1.880501 | 4.773431 | 54.7988 | 39.5441 | 13.9858 | 46.4702 | 16.0503 | 36.6571 | 150.024 | 138.18 | 0.5 | 0.40 | T/T | CC | GG | CC | CT |
| 57 | 22 | TRUE | TRUE | None | 360.143 | 1.16431 | 12.9254 | 2.051369 | 5.481421 | 103.39 | 47.0655 | 13.8596 | 39.0749 | 15.9718 | 31.5803 | 110.544 | 157.92 | 1.2 | 1.20 | T/T | CC | CG | CC | CC |
| 79 | 30 | FALSE | TRUE | Diabetes | 382.669 | 0.6874466 | 14.4888 | 0.527344 | 4.379531 | 39.7494 | 35.7793 | 13.962 | 50.2588 | 17.6276 | 44.8727 | 94.752 | 181.608 | 0.8 | 0.90 | T/T | CT | CG | TT | CT |
| 46 | 19 | FALSE | TRUE | None | 570.505 | 0.6453285 | 14.0068 | 2.271301 | 3.942412 | 37.7947 | 42.0883 | 21.2483 | 36.6634 | 11.4336 | 41.7461 | 86.856 | 205.296 | 1 | 0.90 | T/T | CT | | CC | CC |
| 72 | | TRUE | TRUE | None | | | | | | | | | | | | 86.856 | 157.92 | 5 | 7.50 | T/T | CT | CG | TC | CC |
| 68 | | TRUE | TRUE | Cardiovascular | 299.027 | 0.419796 | 13.377 | 1.031124 | 4.580171 | 43.5247 | 37.761 | 18.5279 | 43.7111 | 16.114 | 47.7629 | 86.856 | 177.66 | 2.1 | 1.40 | T/T | CT | CC | CC | CC |
| 65 | 31 | TRUE | TRUE | Depression Anix | 998.029 | 1.025169 | 17.605 | 1.782471 | 31.67291 | 154.817 | 40.0255 | 19.7873 | 40.1873 | 15.7941 | 53.257 | 94.752 | 193.452 | 0.2 | 0.20 | C/T | CC | | TC | CC |
| 37 | 27 | FALSE | TRUE | None | 381.047 | 0.4913673 | 10.8351 | 1.354359 | 2.955103 | 37.8967 | 31.567 | 19.9038 | 48.5291 | 12.2693 | 38.7081 | 86.856 | 142.128 | 0.5 | 0.30 | | CT | CG | TC | CC |
| 51 | 23 | FALSE | TRUE | None | 275.898 | 1.033188 | 13.5315 | 1.871441 | 8.924434 | 171.291 | 41.3659 | 14.9834 | 43.6507 | 21.1672 | 34.3012 | 118.44 | 173.712 | 0.6 | 0.50 | | CC | CG | TC | CT |
| 63 | 24 | FALSE | TRUE | None | | | | | | | | | | | | 86.856 | 165.816 | 3.1 | 5.20 | T/T | CT | CC | TT | CT |
| 50 | 28 | FALSE | TRUE | None | 364.498 | 1.55085 | 16.8875 | 1.718525 | 34.65599 | 144.259 | 36.821 | 17.0881 | 46.091 | 17.1548 | 43.2661 | 189.504 | 236.88 | 0.4 | 0.50 | T/T | CC | CG | TT | CC |
| 68 | 24 | FALSE | TRUE | Diabetes | 814.071 | 1.619766 | 31.7693 | 1.623886 | 31.39098 | 80.6349 | 38.1966 | 22.7569 | 39.0466 | 17.5423 | 31.6985 | 118.44 | 138.18 | 3.5 | 4.30 | T/T | CT | GG | TC | CT |
| 31 | 25 | TRUE | TRUE | None | 289.248 | 1.354424 | 13.0749 | 2.38623 | 6.824061 | 127.48 | 42.1825 | 18.9765 | 38.841 | 26.6697 | 31.978 | 110.544 | 130.284 | 0.3 | 0.40 | C/C | TT | CC | TT | CC |
| 63 | 28 | FALSE | TRUE | None | 286.76 | 1.030258 | 11.3084 | 1.631271 | 3.195651 | 43.4016 | 31.4552 | 27.8874 | 40.6574 | 21.0996 | 39.7836 | 110.544 | 157.92 | 1.1 | 0.70 | T/T | CT | CC | CC | CC |
| 38 | 20 | FALSE | TRUE | None | 212.557 | | 8.53469 | | 2.530628 | 69.8766 | | | | 19.8794 | | 94.752 | 165.816 | | 0.60 | T/T | CT | CG | TT | CT |
| 24 | 26 | FALSE | TRUE | None | 495.885 | 1.749409 | 12.9979 | 1.734474 | 5.417746 | 74.2438 | 39.6501 | 20.3798 | 39.9701 | 18.7191 | 32.8655 | 86.856 | 142.128 | 0.9 | 1.10 | T/T | TT | CG | TT | CC |
| 44 | 27 | FALSE | TRUE | other | | | | | | | | | | | | 102.648 | | 0.5 | | T/T | CC | CG | TC | CC |
| 26 | 29 | FALSE | TRUE | None | 130.338 | | 5.99849 | | 2.620138 | 33.3862 | | | | 11.1706 | | 94.752 | | 0.6 | | C/T | CT | GG | CC | CT |
| 21 | 24 | FALSE | TRUE | None | 552.466 | 1.441635 | 15.0664 | 2.898083 | 6.25644 | 42.3275 | 33.0805 | 15.5697 | 51.3498 | 14.8229 | 45.868 | 86.856 | 126.336 | 1 | 1.10 | T/T | CC | GG | TC | CC |
| 35 | 28 | FALSE | FALSE | other | 333.293 | 1.511524 | 19.9724 | 2.057388 | 98.24394 | 87.7463 | 35.143 | 30.7736 | 34.0835 | 17.3402 | 51.3817 | 102.648 | 181.608 | 0.5 | 0.60 | C/T | CT | CG | CC | CT |
| 36 | 27 | FALSE | FALSE | None | 342.234 | 1.224606 | 16.204 | 1.637817 | 4.539591 | 26.1128 | 33.229 | 17.2853 | 49.4857 | 16.3224 | 46.229 | 94.752 | 146.076 | 0.3 | 0.30 | | CC | CC | TT | CC |

| | | | | | | | | | | | | | | | | | | | | | | | | |
|---|---|---|---|---|---|---|---|---|---|---|---|---|---|---|---|---|---|---|---|---|---|---|---|---|
| 38 | 27 | FALSE | TRUE | None | 513.008 | 1.339706 | 15.1094 | 1.67038 | 5.276666 | 40.733 | 41.9768 | 18.2693 | 39.7539 | 18.7342 | 40.3594 | 110.544 | 213.192 | 0.6 | 0.50 | T/T | CT | CC | TC | CC |
| 24 | 23 | TRUE | TRUE | None | 138.006 | 0.908638 | 8.52285 | 0.627884 | 39.66022 | 30.1179 | 36.7461 | 27.7075 | 35.5465 | 13.6671 | 47.812 | 78.96 | 169.764 | 0.8 | 0.60 | T/T | CC | CC | TC | CC |
| 24 | 29 | FALSE | TRUE | None | | | | | | | | | | | | 94.752 | 138.18 | 0.4 | 0.50 | T/T | CC | CG | TT | CC |
| 39 | 20 | FALSE | TRUE | None | | | | | | | | | | | | 110.544 | 126.336 | | 0.70 | T/T | CC | CC | TT | CT |
| 38 | 28 | TRUE | TRUE | None | 193.116 | 0.6870155 | 10.4062 | 1.119751 | 7.696188 | 101.432 | 37.8797 | 16.5157 | 45.6046 | 15.4922 | 36.6676 | 118.44 | 169.764 | 0.1 | 1.30 | T/T | CC | | TT | CT |
| 30 | 23 | FALSE | TRUE | None | | | | | | | | | | | | 110.544 | | 0.6 | | T/T | CT | CC | TC | CC |
| 60 | | FALSE | TRUE | Cardiovascular | 284.194 | 0.8227615 | 12.2185 | 1.034081 | 3.140116 | 67.4949 | 33.2424 | 18.3767 | 48.3809 | 16.5518 | 47.5432 | 134.232 | 142.128 | 2.1 | 1.60 | T/T | CC | GG | CC | CC |
| 52 | 33 | FALSE | TRUE | None | | | | | | | | | | | | 94.752 | | 0.7 | | T/T | CC | GG | TC | CC |
| 45 | 26 | FALSE | TRUE | None | 307.604 | 0.947403 | 15.0369 | 1.027016 | 9.500215 | 185.85 | 38.8836 | 17.1753 | 43.9411 | 22.1262 | 31.0185 | 102.648 | 153.972 | 3.1 | 2.60 | T/T | CC | CG | TC | CC |
| 70 | 25 | FALSE | TRUE | None | 210.992 | 1.163521 | 8.62972 | 1.569473 | 2.069835 | 44.1331 | 43.7972 | 16.2551 | 39.9477 | 15.268 | 38.468 | 102.648 | 209.244 | 4.6 | 3.20 | T/T | CC | | TT | CT |
| 60 | 25 | TRUE | TRUE | inflammatory | 358.119 | 1.077343 | 11.5229 | 1.510456 | 14.50167 | 66.4568 | 39.5135 | 20.7474 | 39.7391 | 12.9796 | 41.6267 | 94.752 | 150.024 | 0.9 | 0.50 | T/T | CT | CC | TT | CC |
| 50 | 24 | FALSE | TRUE | None | 521.252 | 1.140572 | 30.1372 | 3.25499 | 4.543124 | 78.9383 | 43.5839 | 19.7128 | 36.7033 | 15.888 | 41.947 | 102.648 | 181.608 | 0.8 | 0.80 | C/T | CC | CC | TC | CC |
| 48 | 26 | TRUE | TRUE | None | 294.455 | 1.161736 | 10.9226 | 0.936127 | 4.341521 | 94.8795 | 37.4104 | 15.8933 | 46.6963 | 14.6834 | 44.7285 | 102.648 | 150.024 | 1.7 | 1.40 | T/T | CT | CG | TC | CC |
| 63 | 24 | FALSE | TRUE | None | 420.725 | 0.8103142 | 10.0738 | 1.242855 | 6.489676 | 57.0422 | 38.7247 | 12.6961 | 48.5792 | 15.9252 | 33.4192 | 118.44 | 213.192 | 1 | 1.00 | T/T | CT | CC | CC | CT |
| 63 | 25 | FALSE | TRUE | Cardiovascular | 820.451 | 1.765831 | 22.0271 | 2.871296 | 8.614553 | 234 | 40.2149 | 13.2942 | 46.491 | 16.4768 | 45.6182 | 86.856 | 153.972 | 4.4 | 3.30 | C/T | CT | | TT | CC |
| 67 | 30 | FALSE | TRUE | Urology | 452.665 | | 11.766 | | 11.40694 | 113.286 | | | | 19.5646 | | 94.752 | | 4.5 | | | CC | | TC | CT |
| 70 | 26 | FALSE | TRUE | Urology | 491.55 | | 10.172 | | 4.928463 | 65.7536 | | | | 14.378 | | 69.4848 | 173.712 | 13.6 | 11.20 | | CC | | CC | CT |
| 50 | 30 | TRUE | TRUE | None | 411.642 | 2.667107 | 13.6097 | 4.421665 | 10.87827 | 65.6093 | 42.9845 | 18.0196 | 38.9959 | 22.5283 | 44.9849 | 110.544 | 157.92 | 4.7 | 4.60 | T/T | CC | GG | TC | CC |
| 56 | 24 | TRUE | TRUE | None | 236.251 | 1.281704 | 12.7372 | 1.623581 | 6.262113 | 74.7194 | 50.5767 | 24.1699 | 25.2534 | 17.9551 | 39.5614 | 118.44 | 181.608 | 1.6 | 1.10 | T/T | CC | CG | CC | CT |
| 80 | 24 | TRUE | TRUE | inflammatory | 349.039 | 1.298065 | 17.8849 | 1.259167 | 5.798305 | 171.466 | 37.2572 | 12.8692 | 49.8737 | 19.767 | 41.4331 | 102.648 | 130.284 | 0.55 | 0.60 | T/T | CT | CC | TC | CC |
| 36 | 23 | FALSE | TRUE | None | 818.735 | 1.118965 | 16.1649 | 1.114716 | 5.344617 | 70.4746 | 33.6242 | 15.1694 | 51.2064 | 20.1072 | 36.5688 | 94.752 | 142.128 | 1.5 | 3.00 | T/T | TT | GG | TC | CC |
| 52 | 30 | TRUE | TRUE | Depression Anix | 249.335 | 2.412071 | 25.8549 | 1.146408 | 8.26597 | 63.0443 | 41.3792 | 11.4305 | 47.1903 | 18.975 | 28.5112 | 126.336 | 142.128 | 0.95 | 3.00 | C/T | CT | | CC | CC |
| 63 | 26 | FALSE | TRUE | Cardiovascular | 285.545 | 0.6420288 | 8.05977 | 1.076702 | 2.670753 | 25.3958 | 35.4399 | 14.3289 | 50.2312 | 16.1979 | 52.7685 | 94.752 | 177.66 | 2.5 | 2.10 | C/T | CC | CC | CC | CT |
| 65 | 23 | TRUE | TRUE | Cardiovascular | 319.822 | 0.6510773 | 13.9384 | 1.082653 | 7.894978 | 73.6047 | 37.2979 | 17.281 | 45.4211 | 15.5818 | 39.1908 | 110.544 | 181.608 | 2.6 | 2.70 | T/T | CC | CC | CC | CC |
| 53 | 35 | FALSE | TRUE | Depression Anix | 690.958 | 0.8088112 | 16.7187 | 1.510376 | 7.295883 | 125.632 | 38.6227 | 13.1295 | 48.2478 | 16.0795 | 46.5801 | 94.752 | 157.92 | 1.8 | 1.50 | T/T | CC | CC | TC | CC |
| 67 | 30 | FALSE | TRUE | inflammatory | 405.801 | 0.4962692 | 9.23564 | 1.118343 | 3.417464 | 33.0745 | 39.0788 | 14.4349 | 46.4864 | 13.2061 | 45.2917 | 94.752 | 153.972 | 0.6 | 0.60 | T/T | CT | CC | TT | CT |
| 63 | 27 | TRUE | TRUE | None | 419.778 | 0.9416504 | 14.2885 | 2.503765 | 2.999235 | 43.926 | 34.6143 | 10.482 | 54.9037 | 16.883 | 40.7083 | 102.648 | 153.972 | 5.9 | 5.00 | T/T | CC | CG | TC | CC |
| 62 | 21 | FALSE | TRUE | Urology | | | | | | | | | | | | 94.752 | 181.608 | 1.7 | 2.10 | | CT | | TT | CT |
| 46 | 28 | TRUE | TRUE | None | 441.036 | 2.001453 | 17.7294 | 1.547817 | 50.73587 | 40.4454 | 39.8467 | 11.1585 | 48.9947 | 15.3174 | 31.9712 | 110.544 | 161.868 | 0.4 | 0.40 | C/T | CT | CC | TC | CT |
| 76 | 21 | FALSE | TRUE | Cardiovascular | | | | | | | | | | | | 134.232 | 240.828 | 6.3 | 5.50 | T/T | CT | CG | CC | CC |
| 68 | 25 | FALSE | FALSE | Urology | 281.733 | | 13.8555 | | 3.859946 | 58.4836 | | | | 16.1065 | | 118.44 | 157.92 | 4.1 | | T/T | CC | CG | TC | CC |
| 71 | 21 | TRUE | TRUE | None | 418.78 | 1.451462 | 18.659 | 1.993702 | 5.485305 | 51.3956 | 38.7387 | 18.5192 | 42.742 | 13.9043 | 37.7386 | 102.648 | 173.712 | 2.3 | 1.80 | T/T | CC | CG | TC | CC |
| 43 | 26 | FALSE | TRUE | None | 347.284 | 1.055023 | 14.7509 | 1.466171 | 2.80954 | 67.7229 | 47.2382 | 25.6452 | 27.1167 | 19.2848 | 46.7513 | 110.544 | 169.764 | 0.4 | 0.30 | T/T | CT | CG | TC | CC |
| 71 | 26 | TRUE | TRUE | Urology | | | | | | | | | | | | 118.44 | 189.504 | 7.2 | | T/T | TT | CC | TC | CC |
| 66 | 23 | FALSE | TRUE | Cardiovascular | 439.654 | 0.6700859 | 10.5478 | 2.268272 | 2.44099 | 50.3426 | 38.3281 | 16.3807 | 45.2912 | 14.4617 | 34.1856 | 110.544 | 201.348 | 2 | 1.50 | T/T | CT | CC | TC | TT |
| 47 | 24 | TRUE | TRUE | None | 244.429 | | 13.889 | | 9.238579 | 54.4973 | | | | 18.9954 | | 110.544 | 157.92 | 0.5 | | T/T | CT | | CC | CT |
| 42 | 32 | TRUE | TRUE | None | 339.748 | 0.4515915 | 11.1725 | 1.103966 | 3.11615 | 25.5665 | 47.7445 | 17.403 | 34.8526 | 12.8096 | 37.7619 | 94.752 | 142.128 | 0.4 | 0.40 | T/T | CC | CC | TC | CC |
| 70 | 30 | TRUE | FALSE | Urology | 1112.99 | 0.6406631 | 17.3666 | 1.051876 | 4.210443 | 43.979 | 37.9259 | 14.955 | 47.119 | 20.6222 | 46.4996 | 102.648 | 157.92 | 4.3 | 3.70 | T/T | CC | CC | TT | CC |
| 66 | 31 | FALSE | TRUE | None | 362.867 | 0.8970451 | 13.5261 | 1.185646 | 5.360114 | 36.8487 | 39.8047 | 10.5111 | 49.6842 | 13.9418 | 34.6315 | 110.544 | 134.232 | 1.1 | 0.70 | T/T | CC | CC | TT | CC |
| 63 | 24 | FALSE | TRUE | Urology | 556.35 | | 18.4819 | | 5.064359 | 78.5541 | | | | 14.439 | | 86.856 | 169.764 | 8.9 | 8.50 | | CT | | TC | CT |
| 37 | 27 | TRUE | TRUE | None | 792.945 | 0.8918877 | 15.8454 | 3.70903 | 8.038447 | 125.24 | 34.8584 | 11.8955 | 53.2462 | 15.6674 | 40.6043 | 110.544 | 146.076 | 0.6 | 0.80 | T/T | CT | CG | CC | CC |
| 68 | | TRUE | FALSE | Cardiovascular | 237.534 | | 11.8125 | | 4.830601 | 28.6656 | | | | 16.6293 | | 78.96 | 177.66 | 1.1 | 1.20 | | CT | | CC | CT |
| 62 | 27 | FALSE | TRUE | Urology | | | | | | | | | | | | 94.752 | 197.4 | 2.2 | 2.60 | | CT | | TC | CC |
| 45 | 27 | FALSE | TRUE | Cardiovascular | 896.641 | 1.626484 | 16.1903 | 1.78875 | 4.177706 | 79.15 | 37.2898 | 18.8638 | 43.8464 | 21.3443 | 43.0517 | 102.648 | 138.18 | 0.3 | 0.30 | T/T | CT | CG | TT | CC |
| 57 | 31 | TRUE | TRUE | Cardiovascular | | | | | | | | | | | | 94.752 | | 0.6 | | T/T | CC | CG | TT | CT |
| 54 | 22 | FALSE | TRUE | None | 775.331 | 0.7043152 | 15.4226 | 2.228176 | 5.549117 | 45.8373 | 39.025 | 12.3112 | 48.6638 | 14.0651 | 47.2755 | 102.648 | 146.076 | 0.4 | 0.30 | T/T | CT | CG | TT | TT |
| 72 | 24 | TRUE | TRUE | Urology | 354.286 | 0.725975 | 10.2776 | 1.468071 | 14.27497 | 234.793 | 39.0746 | 16.0519 | 44.8736 | 21.8112 | 44.733 | 86.856 | 142.128 | 2.8 | 2.90 | T/T | CC | CC | TC | CC |
| 58 | 29 | TRUE | TRUE | Cardiovascular | 435.252 | 1.253891 | 12.9926 | 0.977474 | 4.266591 | 96.2756 | 39.1438 | 19.6799 | 41.1763 | 18.8512 | 48.9923 | 86.856 | 173.712 | 1.2 | 1.00 | T/T | CT | CC | TC | CT |
| 62 | 25 | TRUE | TRUE | None | 320.184 | 0.3617706 | 10.137 | 1.716473 | 3.528962 | 146.412 | 33.9994 | 15.5317 | 50.4689 | 12.5329 | 52.1322 | 157.92 | 134.232 | 1.7 | 1.20 | T/T | CT | CC | TC | CC |
| 67 | 24 | TRUE | TRUE | Cardiovascular | 499.493 | 0.51297 | 14.2556 | 1.316387 | 2.325028 | 55.3073 | 32.43 | 20.8169 | 46.7531 | 19.2146 | 51.512 | 94.752 | 134.232 | 9 | 9.40 | T/T | CT | CC | CC | CC |
| 64 | 30 | FALSE | TRUE | Cardiovascular | 284.649 | 0.5291786 | 9.93441 | 1.170372 | 3.553295 | 73.3865 | 38.6972 | 25.8351 | 35.4677 | 12.7502 | 50.771 | 78.96 | 134.232 | 0.5 | 0.50 | T/T | CT | CC | TC | CC |
| 46 | 27 | FALSE | TRUE | None | 278.026 | 1.575951 | 13.8165 | 1.519562 | 6.945668 | 86.7568 | 48.3251 | 13.6583 | 38.0167 | 28.5147 | 6.44219 | 86.856 | 165.816 | 0.9 | 1.10 | T/T | CC | CC | CC | CT |
| 48 | 22 | FALSE | FALSE | None | | | | | | | | | | | | 126.336 | | 1.6 | | T/T | TT | CC | CC | CC |
| 60 | 24 | TRUE | TRUE | Cardiovascular | | | | | | | | | | | | 94.752 | 157.92 | 1.9 | 1.60 | T/T | CT | CG | TT | CT |
| 48 | 23 | FALSE | TRUE | None | 262.542 | 0.5128784 | 8.10715 | 0.974773 | 2.654872 | 74.8207 | 31.6215 | 14.4839 | 53.8947 | 13.4737 | 41.8758 | 78.96 | 157.92 | 0.7 | 0.50 | T/T | CT | CG | TT | CC |
| 55 | 26 | TRUE | TRUE | None | 307.208 | 1.369812 | 14.0996 | 1.911068 | 7.253755 | 96.5396 | 38.5706 | 11.2014 | 50.228 | 14.9659 | 35.9198 | 78.1704 | 165.816 | 1.7 | 1.40 | T/T | CT | CC | TC | CC |
| 67 | 27 | FALSE | TRUE | None | 210.161 | 0.7765923 | 10.3377 | 1.967628 | 4.082077 | 42.929 | 45.0553 | 13.9451 | 40.9996 | 20.7751 | 40.2054 | 72.6432 | 126.336 | 2.2 | 2.50 | T/T | CC | CG | TC | CT |
| 46 | 30 | FALSE | TRUE | None | | | | | | | | | | | | 78.96 | 126.336 | 0.7 | 0.80 | T/T | CC | CG | TC | CT |
| 28 | 24 | FALSE | TRUE | None | | | | | | | | | | | | 102.648 | | 1.2 | | T/T | CC | CG | TT | CC |
| 68 | 27 | FALSE | FALSE | Cardiovascular | 586.95 | 1.355656 | 14.3185 | 1.642864 | 4.17144 | 55.705 | 34.6242 | 8.89952 | 56.4763 | 17.2851 | 40.9795 | 86.856 | 157.92 | 0.8 | 0.70 | T/T | CC | CG | TC | CT |
| 56 | 24 | FALSE | TRUE | None | 423.081 | 2.335606 | 29.1703 | 3.380337 | 11.51597 | 239.825 | 44.6158 | 25.4303 | 29.9539 | 23.5709 | 37.5891 | 126.336 | 157.92 | 0.6 | 0.80 | T/T | TT | CC | CC | CT |
| 33 | 24 | FALSE | TRUE | None | 651.423 | 0.7499237 | 16.7637 | 2.321323 | 36.76116 | 38.4353 | 39.9072 | 14.9923 | 45.1006 | 13.5812 | 46.9484 | 126.336 | 142.128 | 0.5 | 0.50 | C/T | CC | CG | TC | CC |
| 34 | 32 | FALSE | FALSE | None | 221.324 | 1.270832 | 14.2602 | 1.486774 | 62.06495 | 52.6074 | 41.2801 | 15.0726 | 43.6473 | 15.4313 | 32.8021 | 118.44 | 165.816 | 0.8 | 0.70 | T/T | TT | CC | TT | CC |
| 46 | 28 | FALSE | TRUE | None | 212.332 | 1.043983 | 11.2689 | 3.319088 | 8.153678 | 92.0576 | 37.8496 | 18.0678 | 44.0827 | 15.4794 | 49.157 | 102.648 | 197.4 | 4.2 | 2.50 | T/T | CC | CG | TC | CT |
| 52 | 25 | TRUE | TRUE | None | 87.2771 | 0.0506783 | 20.239 | 1.765202 | 3.301325 | 27.7045 | 36.0285 | 23.2894 | 40.6821 | 21.7512 | 47.126 | 142.128 | 165.816 | 1 | 1.00 | T/T | CT | CG | CC | CC |
| 32 | 24 | FALSE | TRUE | None | 256.858 | 0.5975494 | 12.6663 | 1.41161 | 4.025204 | 47.3624 | 36.3356 | 15.1482 | 48.5162 | 14.1299 | 37.5251 | 102.648 | 173.712 | 0.5 | 0.60 | T/T | CC | CC | TC | CT |
| 70 | 22 | FALSE | TRUE | None | 363.578 | 0.3402061 | 6.81142 | 0.483124 | 1.975748 | 16.437 | 34.7945 | 8.98963 | 56.2159 | 13.9227 | 47.5667 | 86.856 | 126.336 | 1.4 | 1.60 | T/T | CC | GG | CC | CC |
| 52 | 32 | FALSE | TRUE | None | 550.941 | 1.644539 | 16.6842 | 1.468857 | 4.81629 | 46.6299 | 37.7431 | 14.3147 | 47.9422 | 19.6361 | 47.1424 | 86.856 | 126.336 | 0.8 | 0.80 | T/T | CT | CC | TC | CT |
| 49 | 29 | FALSE | TRUE | None | | | | | | | | | | | | 86.856 | 140.549 | 0.7 | | T/T | CC | CG | TC | CT |
| 42 | 29 | TRUE | TRUE | None | | | | | | | | | | | | 65.5368 | | 1.4 | | T/T | CT | CG | TT | CC |
| 47 | 27 | FALSE | FALSE | Depression Anixiety | | | | | | | | | | | | 78.96 | 175.291 | 0.7 | | T/T | CC | CG | CC | CC |
| 36 | 31 | FALSE | FALSE | None | | | | | | | | | | | | 86.856 | | 0.7 | | T/T | CC | CG | TT | CC |
| 53 | 26 | FALSE | TRUE | None | | | | | | | | | | | | 75.8016 | | 0.6 | | C/T | TT | CC | TT | CC |
| 64 | 29 | TRUE | FALSE | None | | | | | | | | | | | | 65.5368 | 142.128 | 4.4 | 4.00 | T/T | CT | GG | TT | CC |
| 55 | 27 | TRUE | FALSE | Cardiovascular | | | | | | | | | | | | 102.648 | | 0.6 | | T/T | CT | CG | CC | CC |
| 41 | 23 | FALSE | TRUE | None | | | | | | | | | | | | 86.856 | 150.024 | 0.8 | | T/T | CT | CC | TC | CT |
| 28 | 29 | FALSE | TRUE | None | | | | | | | | | | | | 72.6432 | 126.336 | 0.5 | | C/T | CT | GG | TC | CC |
| 35 | | FALSE | TRUE | None | | | | | | | | | | | | 86.856 | | 0.5 | | T/T | CT | CG | TT | CC |
| 45 | | FALSE | TRUE | None | | | | | | | | | | | | 86.856 | 134.232 | 0.6 | 0.70 | T/T | TT | CC | TC | CT |
| 30 | 29 | FALSE | TRUE | None | | | | | | | | | | | | 94.752 | 134.232 | 0.3 | 0.30 | T/T | CT | CG | CC | CT |
| 30 | 24 | FALSE | TRUE | None | | | | | | | | | | | | 110.544 | 118.44 | 3.7 | 2.30 | T/T | CT | CG | TC | CT |
| 68 | 27 | TRUE | TRUE | None | 467.976 | | 11.3217 | | 3.131447 | 34.6529 | | | | 11.8507 | | 94.752 | 213.192 | 0.3 | | T/T | CT | GG | TC | CC |
| 30 | 20 | TRUE | TRUE | None | | | | | | | | | | | | 94.752 | | 1.4 | | | CT | CC | TC | CC |
| 58 | 0 | FALSE | TRUE | None | | | | | | | | | | | | 86.856 | | 0.9 | | T/T | CT | CC | TC | CT |
| 29 | 18 | FALSE | FALSE | None | | | | | | | | | | | | 292.152 | | | | C/T | CT | CC | TC | CT |
| 46 | 45 | FALSE | TRUE | None | | | | | | | | | | | | 94.752 | | 0.8 | | T/T | TT | GG | TC | CT |
| 58 | 28 | FALSE | TRUE | Cardiovascular | | | | | | | | | | | | 110.544 | | 0.7 | | T/T | CC | CC | TC | CT |
| 23 | 32 | TRUE | TRUE | None | | | | | | | | | | | | 94.752 | | 0.8 | | T/T | CC | CC | CC | CT |
| 40 | 29 | FALSE | TRUE | None | | | | | | | | | | | | 110.544 | 162.658 | 0.5 | | T/T | CC | CG | TC | CC |
| 58 | 28 | TRUE | FALSE | Urology | 225.752 | | 9.22781 | | 3.87733 | 66.7149 | | | | 20.799 | | 102.648 | 161.868 | 12.3 | 8.90 | | CT | | CC | CT |
| 52 | 28 | FALSE | TRUE | None | 479.492 | 0.376358 | 11.8234 | 3.548546 | 109.9142 | 68.1343 | 34.3883 | 30.4201 | 35.1916 | 16.9458 | 47.4566 | 150.024 | 150.024 | 1.3 | 1.50 | T/T | CC | CG | TC | CC |
| 42 | | TRUE | TRUE | None | | | | | | | | | | | | 102.648 | | 0.8 | | T/T | CC | CC | TC | CT |
| 50 | | FALSE | TRUE | None | | | | | | | | | | | | 94.752 | 134.232 | 1.2 | 0.30 | | CC | CG | TC | CC |
| 50 | | FALSE | TRUE | None | | | | | | | | | | | | 102.648 | 157.13 | 0.8 | | T/T | CT | GG | TC | CC |
| 57 | | TRUE | TRUE | Cardiovascular | 438.285 | | 21.1305 | | 6.271662 | 69.2482 | | | | 23.1131 | | 94.752 | | 0.9 | | C/T | CT | CC | TC | CC |
| 52 | | FALSE | TRUE | None | 469.306 | 1.561714 | 18.7183 | 2.043007 | 61.96082 | 33.6382 | 39.9209 | 13.1693 | 46.9098 | 16.5687 | 38.0209 | 94.752 | 157.92 | 0.3 | 0.30 | T/T | TT | CC | CC | CT |
| 59 | 24 | FALSE | TRUE | None | 613.544 | 1.87291 | 23.7673 | 2.2071 | 8.047801 | 60.5466 | 32.6454 | 8.18319 | 59.1714 | 18.5469 | 45.9029 | 86.856 | 157.92 | 4.7 | 3.60 | | TT | CC | TC | CT |
| 54 | 26 | TRUE | TRUE | Cardiovascular | 236.632 | 0.9863968 | 13.7974 | 0.97205 | 3.559095 | 56.2857 | 42.506 | 22.2665 | 35.2276 | 20.0431 | 37.753 | 110.544 | 213.192 | 1.8 | 2.00 | T/T | CC | CC | TC | CT |
| 60 | 24 | TRUE | TRUE | None | 450.978 | 1.268631 | 11.8947 | 1.055622 | 2.978332 | 53.7519 | 42.9856 | 15.4357 | 41.5787 | 17.172 | 38.6634 | 94.752 | 142.128 | 1.3 | 2.00 | T/T | CT | CC | CC | CC |
| 25 | 22 | FALSE | FALSE | None | | | | | | | | | | | | 86.856 | | 0.7 | | C/T | CC | CG | TT | CC |
| 62 | 28 | TRUE | TRUE | Urology | 443.266 | 0.8444481 | 12.5039 | 2.540508 | 3.598052 | 26.7234 | 47.2579 | 19.3895 | 33.3526 | 13.9058 | 30.6607 | 86.856 | 165.816 | 0.6 | 0.50 | T/T | CC | CG | TC | CC |
| 72 | 25 | TRUE | TRUE | None | 227.844 | | 7.96679 | | 4.620223 | 48.8053 | | | | 15.7175 | | 102.648 | 157.92 | 0.6 | | T/T | CC | CG | CC | CC |
| 44 | 26 | FALSE | TRUE | None | 251.331 | 0.9305687 | 12.6759 | 1.089588 | 3.593163 | 79.6036 | 36.0867 | 15.9263 | 47.987 | 15.0942 | 32.057 | 126.336 | 157.92 | 0.5 | 0.50 | T/T | CT | CG | TT | CC |
| 54 | 27 | FALSE | TRUE | None | | | | | | | | | | | | 86.856 | 165.816 | 3.5 | 0.50 | T/T | CT | CG | CC | CT |
| 62 | 26 | TRUE | TRUE | None | 246.66 | 0.8519859 | 9.23384 | 0.69735 | 18.34918 | 39.0314 | 35.9818 | 7.71484 | 56.3033 | 15.2377 | 39.8246 | 102.648 | 165.816 | 1.4 | 1.10 | T/T | CT | CG | TC | CC |
| 29 | 26 | FALSE | FALSE | other | 346.395 | | 12.9807 | | 3.92333 | 75.9408 | | | | 11.3601 | | 110.544 | 165.816 | 0.4 | 0.50 | T/T | CT | GG | CC | CT |
| 51 | 32 | FALSE | FALSE | None | | | | | | | | | | | | 110.544 | 181.608 | 0.7 | 0.60 | T/T | CT | CG | TC | CC |
| 48 | 24 | FALSE | TRUE | None | | | | | | | | | | | | 142.128 | | 0.5 | | T/T | CC | GG | TC | CC |
| 61 | 29 | TRUE | TRUE | Cardiovascular | 265.584 | 0.6662064 | 13.3135 | 2.07058 | 3.624894 | 33.9986 | 42.0857 | 15.8011 | 42.1132 | 15.5739 | 38.5721 | 110.544 | 165.816 | 1.6 | 1.50 | | CT | CG | TC | CT |
| 46 | 26 | TRUE | FALSE | None | 242.944 | 0.7278633 | 14.5412 | 5.267075 | 53.16935 | 41.7316 | 35.7309 | 18.7185 | 45.5507 | 22.1615 | 43.64 | 110.544 | 189.504 | 0.2 | 0.20 | T/T | CT | GG | TT | CC |
| 57 | 21 | FALSE | TRUE | None | 207.973 | 0.4109383 | 7.54794 | 1.375618 | 2.757874 | 34.0089 | 35.6915 | 20.2156 | 44.0929 | 20.4111 | 55.3707 | 102.648 | 150.024 | 0.2 | 0.20 | T/T | CC | CC | TC | CT |
| 73 | | FALSE | FALSE | Urology | 713.967 | | 17.6654 | | 8.863234 | 50.4474 | | | | 18.1413 | | 110.544 | 142.128 | 9.6 | 12.00 | | CT | | CC | CC |
| 34 | 26 | FALSE | TRUE | None | 812.163 | 0.7340508 | 28.8379 | 2.696232 | 6.099884 | 54.1125 | 35.3353 | 13.8491 | 50.8156 | 21.1779 | 41.2452 | 94.752 | 134.232 | 0.65 | 0.70 | T/T | TT | | TC | CC |
| 71 | 32 | FALSE | TRUE | Urology | 290.595 | | 8.41998 | | 12.8788 | 142.655 | | | | 16.8704 | | 134.232 | 205.296 | 0.1 | 0.20 | | CT | | TC | CC |
| 63 | 27 | TRUE | TRUE | Cardiovascular | 563.072 | 0.3206673 | 26.7494 | 0.75457 | 29.73463 | 207.22 | 35.6669 | 5.6098 | 58.7233 | 15.322 | 28.4832 | 118.44 | 134.232 | 2.4 | 2.60 | T/T | CC | CG | TC | CC |
| 63 | 36 | FALSE | TRUE | Urology | 412.064 | | 9.45985 | | 9.014795 | 71.555 | | | | 19.812 | | 94.752 | 205.296 | 1.6 | 2.60 | | CC | | | CC |
| 45 | 25 | FALSE | TRUE | other | 316.233 | 1.256622 | 13.1277 | 1.118927 | 4.442219 | 73.779 | 34.6806 | 12.9455 | 52.3739 | 17.5265 | 37.1632 | 106.596 | 181.608 | 0.6 | 0.80 | T/T | CC | CG | TC | CC |
| 73 | 24 | FALSE | TRUE | Cardiovascular | 205.944 | 0.9183006 | 13.966 | 1.702679 | 4.771697 | 61.8375 | 33.2802 | 13.7021 | 53.0177 | 13.2614 | 36.8065 | 90.804 | 157.92 | 2.1 | 4.30 | T/T | CC | CC | TT | CC |
| 75 | 21 | FALSE | TRUE | Cardiovascular | 315.475 | 0.4758682 | 13.2511 | 1.644081 | 5.751276 | 49.1111 | 39.3698 | 13.324 | 47.3062 | 19.7245 | 39.4356 | 75.8016 | 173.712 | 1.8 | 1.70 | T/T | CC | CC | TT | TT |
| 77 | 26 | FALSE | FALSE | None | 163.118 | 0.6969833 | 13.7647 | 1.17432 | 5.1404 | 111.75 | 40.8532 | 7.93026 | 51.2166 | 18.5166 | 47.1978 | 90.804 | 181.608 | 1.2 | 1.40 | T/T | CT | GG | TT | CC |
| 71 | 27 | FALSE | FALSE | Cardiovascular | 1335.12 | 0.8617668 | 15.4237 | 2.244633 | 5.201451 | 44.1466 | 31.237 | 11.1615 | 57.6015 | 11.9937 | 47.899 | 118.44 | 221.088 | 1.6 | 2.50 | T/T | CT | CC | TC | CT |
| 49 | 23 | FALSE | TRUE | None | 926.021 | 0.3658218 | 12.2032 | 1.67469 | 4.32273 | 39.9771 | 29.9319 | 34.5028 | 35.5653 | 15.5311 | 58.1749 | 98.7 | 221.088 | 0.8 | 0.90 | T/T | CC | GG | TC | CC |
| 51 | 25 | FALSE | TRUE | None | 492.058 | 0.9376526 | 15.9205 | 3.335217 | 4.574255 | 90.0829 | 41.996 | 20.7857 | 37.2183 | 18.852 | 40.7873 | 98.7 | 205.296 | 1.6 | 1.50 | T/T | TT | GG | TC | CC |
| 68 | 22 | FALSE | TRUE | None | 296.719 | 0.3213463 | 10.7639 | 2.472328 | 6.711939 | 57.9171 | 39.4872 | 25.4463 | 35.0665 | 14.2342 | 46.4758 | 106.596 | 173.712 | 0.9 | 1.10 | T/T | CC | CG | TT | CC |
| 62 | 30 | FALSE | TRUE | None | 182.44 | 0.7460709 | 9.71748 | 1.078484 | 2.762567 | 265.308 | 37.8774 | 30.6669 | 31.4556 | 21.7964 | 35.7143 | 153.972 | 165.816 | 7.8 | 5.20 | T/T | CT | CG | TT | CC |
| 67 | 22 | FALSE | TRUE | Cardiovascular | 521.713 | 1.295341 | 13.2101 | 2.604481 | 3.634142 | 55.0079 | 35.2221 | 31.635 | 33.1428 | 15.3788 | 36.4602 | 98.7 | 189.504 | 6.5 | 7.20 | T/T | CC | CG | TT | CT |
| 44 | 27 | TRUE | TRUE | None | 541.994 | 1.601505 | 16.8245 | 1.960938 | 6.17415 | 51.6316 | 36.4842 | 11.8018 | 51.714 | 17.609 | 33.8144 | 86.856 | 157.92 | 0.4 | 0.50 | T/T | CT | CC | CC | CC |
| 40 | 21 | FALSE | TRUE | None | 2098.51 | 0.6650162 | 15.6506 | 3.346367 | 4.51637 | 84.0559 | 44.6776 | 27.2941 | 28.0283 | 15.561 | 38.1905 | 134.232 | 213.192 | 0.6 | 0.60 | T/T | CT | CC | CC | CT |
| 68 | 29 | FALSE | TRUE | None | 261.16 | 1.140251 | 11.4328 | 0.979256 | 4.231188 | 40.9943 | 31.0351 | 10.7169 | 58.248 | 13.021 | 50.2476 | 90.804 | 189.504 | 5.5 | 5.30 | T/T | CT | CG | TC | CT |
| 66 | 32 | FALSE | FALSE | Urology | | | | | | | | | | | | 110.544 | 205.296 | 2.6 | 2.30 | | CC | | TT | CT |
| 78 | 26 | FALSE | TRUE | Cardiovascular | 376.863 | 0.6813507 | 11.3356 | 1.850559 | 9.023574 | 62.6606 | 36.603 | 12.0958 | 51.3012 | 12.7562 | 41.4283 | 110.544 | 197.4 | 5.2 | 5.80 | T/T | CC | CG | TT | CC |
| 74 | 24 | TRUE | TRUE | Cardiovascular | 313.897 | 1.007816 | 11.647 | 0.599201 | 7.049828 | 93.9302 | 40.2147 | 10.3349 | 49.4504 | 16.498 | 38.199 | 86.856 | 165.816 | 4.6 | 4.90 | T/T | CT | CG | CC | CC |
| 68 | 24 | FALSE | TRUE | Urology | 340.547 | | 17.6186 | | 5.149005 | 76.0243 | | | | 17.5073 | | 114.492 | 173.712 | 7 | 9.60 | | CC | | CC | CC |
| 45 | 26 | FALSE | TRUE | None | 618.069 | | 14.1318 | | 4.115648 | 173.596 | | | | 16.5928 | | 106.596 | | 0.7 | | T/T | CC | GG | TT | CT |
| 27 | 28 | FALSE | FALSE | other | | | | | | | | | | | | 106.596 | | 2.2 | | T/T | CT | CC | CC | CT |
| 45 | 25 | FALSE | TRUE | inflammatory | 229.051 | 0.277874 | 13.6724 | 0.796047 | 3.400284 | 56.967 | 33.4034 | 12.4268 | 54.1699 | 18.4024 | 42.7446 | 90.804 | 181.608 | 1.8 | 1.70 | T/T | CC | CG | TT | CC |
| 61 | 22 | TRUE | TRUE | Depression Anix | 783.459 | | 15.6321 | | 8.173537 | 34.7732 | | | | 20.3619 | | 106.596 | | 1.5 | | T/T | CT | GG | CC | CC |
| 52 | 22 | FALSE | TRUE | Cardiovascular | 502.468 | 1.499561 | 16.6635 | 3.446186 | 16.98425 | 98.3905 | 36.834 | 13.1686 | 49.9974 | 19.4028 | 52.6346 | 122.388 | 181.608 | 1.1 | 1.20 | T/T | CT | CC | TC | CC |
| 35 | 20 | FALSE | TRUE | None | | | | | | | | | | | | 142.128 | 152.393 | 0.3 | | T/T | CC | CG | CC | CC |
| 62 | 27 | FALSE | TRUE | None | | | | | | | | | | | | 114.492 | 213.192 | 2.3 | 2.00 | T/T | CC | CG | TC | CT |
| 42 | 26 | FALSE | TRUE | None | 644.362 | 0.9756966 | 15.6982 | 1.554684 | 12.12293 | 91.5304 | 42.62 | 20.1087 | 37.2713 | 17.1248 | 44.6508 | 114.492 | 173.712 | 0.8 | 0.90 | T/T | CC | CG | TC | CC |
| 65 | 23 | FALSE | FALSE | Diabetes | | | | | | | | | | | | 126.336 | 189.504 | 2 | 1.30 | T/T | CC | CC | CC | CC |
| 53 | 25 | FALSE | TRUE | None | 607.016 | 0.3633652 | 12.3534 | 1.542816 | 4.029985 | 80.1611 | 34.07 | 19.786 | 46.144 | 17.3432 | 44.7978 | 102.648 | 181.608 | 0.4 | 0.50 | T/T | CT | CC | TT | CC |
| 60 | 24 | TRUE | TRUE | None | | | | | | | | | | | | 94.752 | | 1.1 | | T/T | CC | CG | TT | TT |
| 26 | 26 | FALSE | TRUE | inflammatory | | | | | | | | | | | | 86.856 | | 0.4 | | T/T | CC | CG | CC | CT |
| 25 | 22 | TRUE | TRUE | None | | | | | | | | | | | | 110.544 | | 0.3 | | C/C | CC | GG | TC | CC |
| 46 | 21 | TRUE | TRUE | None | | | | | | | | | | | | 75.8016 | 126.336 | 1 | 1.60 | T/T | CC | | TC | CC |
| 50 | 32 | TRUE | TRUE | Cardiovascular | | | | | | | | | | | | 86.856 | 110.544 | 1.1 | 1.00 | T/T | CC | CG | TT | CC |
| 40 | 24 | FALSE | TRUE | None | | | | | | | | | | | | 102.648 | | 0.9 | | T/T | CC | | TT | CC |
| 70 | 31 | FALSE | TRUE | None | | | | | | | | | | | | 86.856 | | 0.7 | | T/T | CT | CG | TC | CT |
| 47 | 24 | TRUE | TRUE | None | | | | | | | | | | | | 86.856 | 157.92 | 0.9 | 0.10 | T/T | CC | CC | CC | CC |
| 47 | 24 | TRUE | TRUE | None | | | | | | | | | | | | 102.648 | 161.868 | 1 | | T/T | CC | CC | TT | CC |
| 25 | 28 | TRUE | TRUE | None | | | | | | | | | | | | 78.96 | | 0.6 | | C/T | CC | CG | TT | CC |
| 27 | 30 | TRUE | TRUE | None | | | | | | | | | | | | 94.752 | | 0.3 | | C/T | CC | GG | TC | CC |
| 59 | 35 | TRUE | TRUE | other | | | | | | | | | | | | 118.44 | 142.128 | 1.5 | 0.90 | T/T | CC | CC | TC | CC |
| 32 | 30 | FALSE | TRUE | None | | | | | | | | | | | | 94.752 | 102.648 | 0.5 | 0.60 | T/T | CC | CG | TC | CT |

| | | | | | | | | | | | | | | | | | | | | | | | | |
|---|---|---|---|---|---|---|---|---|---|---|---|---|---|---|---|---|---|---|---|---|---|---|---|---|
| 42 | 30 | FALSE | FALSE | None | | | | | | | | | | | | 86.856 | | 0.5 | | T/T | CT | | CC | CC |
| 50 | 27 | TRUE | TRUE | None | | | | | | | | | | | | 94.752 | 157.92 | 0.7 | 0.70 | T/T | CT | | TC | CC |
| 48 | 20 | FALSE | TRUE | None | | | | | | | | | | | | 102.648 | | 0.9 | | T/T | TT | CC | CC | CT |
| 46 | 25 | FALSE | TRUE | None | | | | | | | | | | | | 102.648 | 150.024 | 1.1 | 1.00 | T/T | CC | CC | TC | TT |
| 28 | 34 | FALSE | TRUE | None | | | | | | | | | | | | 94.752 | | 0.4 | | T/T | CT | CC | TC | CT |
| 53 | 29 | FALSE | TRUE | None | | | | | | | | | | | | 118.44 | 134.232 | 0.3 | 0.40 | C/T | CC | CG | TC | CC |
| 29 | 23 | TRUE | TRUE | None | | | | | | | | | | | | 102.648 | | 0.4 | | T/T | CT | CC | TT | CC |
| 20 | 21 | TRUE | TRUE | None | | | | | | | | | | | | 110.544 | | 0.3 | | T/T | CT | CG | CC | CC |
| 20 | | FALSE | TRUE | None | | | | | | | | | | | | 110.544 | 150.024 | 0.4 | 0.40 | T/T | CC | CC | TT | CC |
| 41 | 30 | FALSE | TRUE | None | | | | | | | | | | | | 102.648 | | 0.3 | | T/T | CT | CC | CC | CC |
| 51 | 29 | FALSE | TRUE | Depression Anixiety | | | | | | | | | | | | 134.232 | 189.504 | 0.9 | 0.90 | T/T | TT | CG | TC | CT |
| 40 | 22 | TRUE | FALSE | None | | | | | | | | | | | | 118.44 | | 1 | | T/T | CC | CG | TC | CC |
| 49 | 37 | TRUE | TRUE | None | | | | | | | | | | | | 102.648 | 157.92 | 0.9 | 0.90 | T/T | CC | CC | TC | CC |
| 36 | 27 | FALSE | TRUE | None | | | | | | | | | | | | 102.648 | 165.816 | 0.5 | 0.60 | T/T | CT | CC | TT | CT |
| 55 | 22 | FALSE | TRUE | None | | | | | | | | | | | | 110.544 | 197.4 | 1.7 | 1.90 | T/T | CC | CC | TC | CT |
| 35 | 22 | FALSE | TRUE | None | | | | | | | | | | | | 102.648 | 150.024 | 0.4 | 0.40 | T/T | CC | GG | TC | CC |
| 47 | 32 | FALSE | TRUE | other | 379.239 | | 15.4849 | | 3.006965 | 85.0161 | | | | 21.9418 | | 110.544 | | 0.8 | | T/T | CT | CG | TC | CC |
| 35 | 26 | FALSE | TRUE | None | | | | | | | | | | | | 98.7 | | 0.3 | | C/T | CC | CG | TC | CC |
| 73 | 35 | TRUE | TRUE | Diabetes | 503.801 | 1.439159 | 12.5787 | 1.3951 | 5.537738 | 54.8558 | 42.2353 | 22.9717 | 34.793 | 20.7902 | 49.2781 | 114.492 | 118.44 | 1.2 | 0.60 | T/T | CC | GG | TC | CC |
| 46 | 30 | FALSE | FALSE | other | 549.899 | 0.7394371 | 19.0531 | 2.528774 | 9.05467 | 104.732 | 36.5178 | 11.3677 | 52.1146 | 16.2296 | 43.4744 | 134.232 | 173.712 | 0.5 | 0.40 | T/T | TT | CG | TC | CC |
| 45 | 30 | TRUE | FALSE | None | | | | | | | | | | | | 122.388 | 229.774 | 4.4 | | T/T | TT | CG | TC | CT |
| 38 | 28 | TRUE | TRUE | Depression Anix | 594.283 | | 21.916 | | 5.558982 | 90.9532 | | | | 19.8648 | | 114.492 | 173.712 | | 0.40 | T/T | CT | GG | CC | CC |
| 42 | 26 | FALSE | TRUE | None | | | | | | | | | | | | 146.076 | 150.024 | 0.3 | 0.40 | T/T | CT | | TC | CT |
| 64 | 25 | FALSE | TRUE | Urology | 355.358 | | 16.9329 | | 10.84304 | 72.1673 | | | | 17.0493 | | 110.544 | 134.232 | 8.8 | 11.30 | | TT | | TC | CC |
| 39 | 24 | FALSE | TRUE | None | | | | | | | | | | | | 106.596 | | 0.9 | | T/T | CT | CG | TT | CC |
| 51 | 27 | TRUE | TRUE | None | 223.672 | | 11.4947 | | 33.20979 | 30.0572 | | | | 14.8912 | | 94.752 | | 0.3 | | | | | | CC |
| 43 | 26 | FALSE | TRUE | None | | | | | | | | | | | | 110.544 | | 0.9 | | T/T | CT | CG | TT | CC |
| 62 | 23 | FALSE | TRUE | Cardiovascular | | | | | | | | | | | | 102.648 | 146.076 | 5.6 | | T/T | CT | CC | TC | CC |
| 46 | 25 | FALSE | TRUE | None | 251.434 | | 14.7588 | | 42.44923 | 32.4884 | | | | 16.819 | | 98.7 | | 0.6 | | T/T | CT | CC | CC | CC |
| 48 | 25 | FALSE | TRUE | None | | | | | | | | | | | | 110.544 | 153.972 | | 0.70 | T/T | CC | CG | TC | CC |
| 58 | 31 | FALSE | TRUE | None | 226.313 | 0.4102707 | 10.5576 | 1.442383 | 2.423298 | 62.6201 | 39.8792 | 18.3322 | 41.7887 | 16.285 | 51.1743 | 118.44 | 150.024 | 0.8 | 1.00 | T/T | CT | CG | TC | CC |
| 61 | 27 | FALSE | TRUE | None | 245.514 | 1.112469 | 15.284 | 1.441891 | 6.979057 | 107.909 | 39.8488 | 13.9012 | 46.25 | 15.153 | 30.4743 | 94.752 | 205.296 | 1.1 | 0.90 | T/T | CC | GG | CC | CC |
| 71 | 32 | FALSE | TRUE | None | 257.667 | 0.6160583 | 10.6265 | 1.232109 | 3.004575 | 58.6104 | 35.1032 | 7.6944 | 57.2024 | 12.9169 | 43.7642 | 110.544 | 161.078 | 17 | 19.70 | T/T | CT | GG | TT | CT |
| 44 | 26 | FALSE | TRUE | Depression Anix | 231.105 | | 12.0804 | | 4.63054 | 45.3807 | | | | 13.7763 | | 118.44 | 223.45 | 1.2 | | T/T | CT | CC | CC | CT |
| 64 | 26 | FALSE | TRUE | other | 382.981 | 1.263737 | 13.4036 | 1.218212 | 5.968865 | 69.7352 | 36.1274 | 15.3062 | 48.5664 | 17.0989 | 35.923 | 110.544 | 195.031 | 2 | 1.90 | T/T | CT | GG | TT | CC |
| 59 | 22 | FALSE | TRUE | None | 185.43 | | 12.4577 | | 5.776084 | 100.041 | | | | 19.7544 | | 102.648 | 190.294 | 0.9 | | T/T | CC | CG | TC | CC |
| 46 | 31 | FALSE | TRUE | other | 240.005 | | 9.45571 | | 3.136942 | 58.2491 | | | | 13.9387 | | 94.752 | 178.45 | | 0.60 | T/T | CT | CC | TC | CC |
| 46 | 25 | FALSE | FALSE | None | 288.667 | | 12.6283 | | 4.007227 | 35.8424 | | | | 16.5683 | | 94.752 | 168.974 | | 0.30 | T/T | CT | CC | TT | TT |
| 55 | 26 | FALSE | TRUE | Cardiovascular | 298.243 | 0.32061 | 14.1422 | 1.483192 | 4.288051 | 38.879 | 31.4437 | 27.7505 | 40.8059 | 15.2449 | 42.2364 | 102.648 | 206.875 | 0.6 | 0.70 | T/T | CT | GG | CC | CC |
| 74 | 24 | FALSE | TRUE | None | 666.744 | | 11.4553 | | 2.634038 | 45.1623 | | | | 13.2548 | | 110.544 | 185.556 | | 1.90 | T/T | CC | CC | CC | TT |
| 64 | 21 | FALSE | TRUE | Urology | | | | | | | | | | | | 81.7236 | 168.185 | 0.7 | 0.70 | | | CC | | |
| 70 | 23 | FALSE | FALSE | Cardiovascular | | | | | | | | | | | | 78.96 | 180.029 | | 1.60 | T/T | CT | CG | TC | CC |
| 62 | 20 | FALSE | TRUE | None | | | | | | | | | | | | 157.92 | 191.083 | 0.9 | 1.20 | T/T | CT | CG | TC | CC |
| 51 | 36 | FALSE | TRUE | inflammatory | | | | | | | | | | | | 134.232 | 142.918 | 0.9 | 0.80 | T/T | CC | CG | CC | CT |
| 50 | 30 | FALSE | TRUE | None | 360.333 | 0.689743 | 8.84478 | 1.816452 | 2.580775 | 43.7901 | 38.1945 | 17.3536 | 44.452 | 14.3092 | 34.8395 | 150.024 | 122.388 | 0.3 | 0.80 | T/T | CT | CG | TC | CC |
| 61 | 30 | TRUE | TRUE | None | 644.167 | 0.2515564 | 11.892 | 1.652313 | 3.243545 | 37.9151 | 43.5857 | 30.448 | 25.9663 | 11.0118 | 39.9549 | 157.92 | 232.932 | 2.4 | 2.70 | T/T | CT | CG | CC | CT |
| 60 | 35 | FALSE | TRUE | Cardiovascular | 538.668 | | 8.20495 | | 2.074394 | 37.7169 | | | | 18.072 | | 150.024 | | 0.8 | | T/T | CC | CG | CC | CC |
| 71 | 21 | FALSE | TRUE | Cardiovascular | 535.948 | 1.635269 | 13.8008 | 1.565544 | 4.647317 | 32.4381 | 36.2929 | 19.3613 | 44.3458 | 19.9544 | 46.0569 | 142.128 | 173.712 | 6.5 | 8.10 | T/T | CC | CG | CC | CC |
| 72 | 29 | FALSE | TRUE | Cardiovascular | | | | | | | | | | | | 142.128 | 134.232 | 3.6 | 5.00 | T/T | CC | CG | TC | CC |
| 40 | 17 | FALSE | FALSE | None | 560.829 | 0.9843826 | 15.9179 | 1.847313 | 3.283863 | 36.6306 | 41.9735 | 19.2216 | 38.8048 | 14.7346 | 43.6272 | 157.92 | 210.034 | 1.2 | 1.50 | C/T | CC | CG | TC | CT |
| 58 | 29 | FALSE | TRUE | None | 748.476 | 1.33794 | 18.9281 | 2.335567 | 7.136841 | 89.4305 | 35.3072 | 16.359 | 48.3338 | 18.2833 | 38.9152 | 150.024 | 162.658 | 0.8 | 0.60 | T/T | CT | CG | TC | CC |
| 54 | 30 | FALSE | TRUE | Cardiovascular | | | | | | | | | | | | 126.336 | | 0.8 | 1.10 | T/T | CC | CC | TC | CT |
| 77 | 28 | TRUE | TRUE | Cardiovascular | | | | | | | | | | | | 126.336 | 250.303 | 1.4 | | T/T | CT | CG | TC | CT |
| 54 | 35 | FALSE | TRUE | None | 409.276 | 0.4372482 | 11.1345 | 2.105118 | 2.566432 | 49.7007 | 37.3256 | 14.7843 | 47.8901 | 16.224 | 39.8129 | 126.336 | 182.398 | 1.3 | 1.20 | T/T | CC | | TC | CC |
| 61 | 30 | TRUE | TRUE | Cardiovascular | 196.594 | 1.484783 | 14.4847 | 1.295944 | 7.66083 | 60.4723 | 40.2595 | 12.1805 | 47.5599 | 19.2234 | 23.7752 | 126.336 | 145.286 | 0.5 | 0.50 | T/T | CC | CG | TC | TT |
| 63 | 24 | FALSE | TRUE | None | | | | | | | | | | | | 110.544 | 157.92 | 1 | | T/T | CT | CG | TC | CC |
| 65 | 28 | TRUE | TRUE | Cardiovascular | 263.333 | 0.4762497 | 35.177 | 1.360344 | 5.851267 | 80.9044 | 33.1313 | 9.4957 | 57.373 | 15.924 | 37.3668 | 118.44 | 151.603 | 0.4 | 0.90 | T/T | CT | CC | TT | CC |
| 52 | 25 | FALSE | TRUE | None | 448.609 | 1.243973 | 13.231 | 1.325474 | 7.506447 | 287.804 | 36.3657 | 18.5339 | 45.1004 | 20.4702 | 35.5484 | 157.92 | 179.239 | 0.7 | 0.90 | T/T | CC | CG | TC | CT |
| 69 | 27 | TRUE | TRUE | Urology | | | | | | | | | | | | 102.648 | 155.551 | 61.7 | 4.90 | | CC | | TT | TT |
| 66 | 32 | FALSE | TRUE | None | 381.37 | 1.49474 | 16.1238 | 1.77578 | 9.499187 | 112.162 | 35.5583 | 20.4165 | 44.0252 | 17.5649 | 40.4514 | 110.544 | 184.766 | 0.8 | 0.40 | T/T | CT | CG | TT | CT |
| 54 | 24 | TRUE | TRUE | None | 539.535 | 0.8476334 | 10.5205 | 1.827499 | 5.500001 | 63.545 | 42.1729 | 13.3551 | 44.472 | 15.0257 | 40.2647 | 134.232 | 123.967 | 1.3 | 1.00 | T/T | CT | GG | TC | CT |
| 52 | 27 | FALSE | TRUE | Cardiovascular | 390.093 | 0.558609 | 16.4192 | 1.440029 | 4.192673 | 96.9667 | 38.5322 | 22.536 | 38.9317 | 14.0701 | 43.4255 | 150.024 | 194.242 | 0.5 | 0.40 | C/T | CC | GG | TC | CC |
| 53 | 28 | FALSE | FALSE | inflammatory | 425.428 | 1.116943 | 13.6864 | 2.271667 | 3.888225 | 56.6311 | 38.3965 | 16.9405 | 44.663 | 12.8463 | 46.4486 | 118.44 | 180.818 | 2.6 | 2.80 | T/T | CC | GG | CC | TT |
| 60 | 26 | TRUE | TRUE | None | 180.044 | | 7.7005 | | 1.629515 | 25.019 | | | | 11.4873 | | 110.544 | | 0.7 | | T/T | CT | CG | TC | CC |
| 65 | 30 | TRUE | TRUE | Cardiovascular | 289.634 | 0.5387077 | 13.8157 | 1.009171 | 2.122751 | 45.2378 | 33.8205 | 19.7787 | 46.4008 | 14.0151 | 49.7664 | 150.024 | 210.823 | 0.8 | 0.80 | T/T | TT | CG | CC | CC |
| 48 | 23 | FALSE | TRUE | Cardiovascular | 852.456 | 0.6418953 | 13.803 | 1.182159 | 6.783602 | 121.577 | 35.9654 | 35.8016 | 28.233 | 16.1448 | 33.9825 | 134.232 | 183.977 | 1.7 | 0.80 | T/T | CT | CC | TT | CT |
| 75 | 33 | FALSE | TRUE | Cardiovascular | 210.275 | | 7.76884 | | 6.615648 | 38.3592 | | | | 17.7873 | | 102.648 | 179.239 | 1.5 | 1.50 | T/T | CC | CC | TC | CT |
| 59 | 25 | TRUE | TRUE | None | 305.813 | | 14.4175 | | 5.044188 | 80.7084 | | | | 24.6297 | | 195.031 | | | | T/T | CT | CG | TT | CC |
| 45 | 26 | TRUE | TRUE | None | 378.679 | 0.7998734 | 13.3369 | 3.204948 | 4.953541 | 64.8383 | 36.5379 | 16.2311 | 47.231 | 13.8516 | 42.6434 | 118.44 | 172.922 | 2.2 | 1.40 | T/T | CC | CG | CC | CT |
| 73 | 34 | TRUE | TRUE | Diabetes | 250.819 | 0.615757 | 11.6114 | 1.547981 | 42.10306 | 24.5637 | 39.745 | 22.2102 | 38.0448 | 12.002 | 25.0113 | 134.232 | 197.4 | 2.2 | 2.70 | T/T | CC | CC | TT | CC |
| 40 | 25 | FALSE | TRUE | None | 399.851 | 0.5594788 | 16.9568 | 1.697845 | 16.82482 | 88.3604 | 41.4412 | 16.6976 | 41.8613 | 21.7094 | 30.4617 | 118.44 | 174.502 | 0.6 | 0.60 | T/T | CC | CG | TC | CC |
| 75 | 22 | FALSE | TRUE | None | 961.589 | 0.5877304 | 14.469 | 1.13615 | 2.70171 | 85.8391 | 36.2293 | 23.4701 | 40.3006 | 13.0831 | 46.8714 | 118.44 | 220.298 | 0.4 | 0.40 | T/T | CT | CG | TC | TT |
| 50 | 23 | TRUE | TRUE | None | 416.959 | 1.327042 | 12.6 | 1.231918 | 3.696673 | 39.1743 | 39.6891 | 18.517 | 41.794 | 14.569 | 36.7292 | 134.232 | 224.246 | 0.7 | 0.70 | T/T | CT | GG | TC | CC |
| 77 | 25 | TRUE | FALSE | other | 431.874 | 1.595219 | 11.1151 | 1.053337 | 8.563519 | 59.4163 | 31.3509 | 21.7093 | 46.9398 | 16.5776 | 38.3527 | 63.9576 | 161.078 | 4 | 0.80 | T/T | TT | | TC | CC |
| 41 | 28 | FALSE | TRUE | None | 188.275 | 0.7743835 | 15.124 | 1.086475 | 4.15272 | 48.5771 | 36.9761 | 9.93863 | 53.0853 | 16.1037 | 37.1327 | 102.648 | 166.606 | 0.3 | 0.40 | T/T | TT | CG | TT | CC |
| 41 | 31 | FALSE | TRUE | Cardiovascular | | | | | | | | | | | | 102.648 | 128.705 | 0.8 | | C/T | CT | CG | TC | CT |
| 47 | 37 | TRUE | FALSE | None | 808.421 | | 29.9891 | | 8.000285 | 97.0221 | | | | 18.7806 | | 134.232 | | 0.3 | | T/T | CC | CG | TC | CC |
| 53 | 29 | FALSE | TRUE | None | 715.42 | | 13.9934 | | 7.237896 | 107.523 | | | | 19.211 | | 102.648 | 152.393 | 4.7 | | T/T | CT | GG | TT | CT |
| 64 | 24 | FALSE | TRUE | None | 430.869 | | 8.35798 | | 2.347326 | 80.8282 | | | | 13.988 | | 86.856 | | 2.5 | | T/T | CT | CC | TT | CT |
| 30 | 26 | TRUE | TRUE | None | | | | | | | | | | | | 94.752 | 152.393 | 0.8 | 0.80 | T/T | CT | CC | TT | CC |
| 60 | 31 | FALSE | TRUE | Cardiovascular | 1260.68 | 0.8579445 | 18.3047 | 2.2612 | 12.99525 | 66.5228 | 35.8694 | 16.4849 | 47.6457 | 15.4342 | 39.848 | 86.856 | 159.499 | 4.9 | 4.60 | T/T | TT | CC | CC | CT |
| 49 | 23 | TRUE | TRUE | None | 437 | 0.3669434 | 12.4441 | 0.734638 | 3.27573 | 40.0631 | 35.5423 | 10.6262 | 53.8315 | 15.1734 | 37.9314 | 102.648 | 192.662 | 1 | 0.70 | T/T | CT | CC | TT | CC |
| 42 | 23 | FALSE | TRUE | None | 383.564 | | 14.7359 | | 3.169663 | 49.2978 | | | | 16.8966 | | 102.648 | 162.66 | 0.5 | | T/T | TT | CC | TC | CC |
| 33 | 25 | FALSE | TRUE | None | 347.095 | | 18.0869 | | 10.47233 | 78.3572 | | | | 18.9099 | | 139.759 | | | | T/T | CC | | TC | CC |
| 48 | 23 | TRUE | TRUE | None | 487.018 | | 9.98656 | | 5.209574 | 73.7392 | | | | 16.3334 | | 120.809 | 118.44 | | 0.80 | T/T | TT | CG | TC | CC |
| 28 | 28 | FALSE | TRUE | None | | | | | | | | | | | | 91.5936 | | | | T/T | CC | CG | TT | CT |
| 73 | 28 | FALSE | TRUE | Cardiovascular | | | | | | | | | | | | 112.123 | 159.499 | | | T/T | CC | GG | TT | CC |
| 34 | 28 | FALSE | TRUE | None | 216.495 | 0.19841 | 12.5141 | 2.582954 | 4.574492 | 26.5881 | 42.8923 | 13.9433 | 43.1644 | 14.8448 | 41.1011 | 116.071 | 112.123 | 0.9 | 1.20 | T/T | CT | CG | TT | CT |
| 34 | 29 | FALSE | TRUE | inflammatory | | | | | | | | | | | | 106.596 | | | | C/T | CT | CG | TC | CT |
| 30 | 26 | FALSE | TRUE | None | | | | | | | | | | | | 92.3832 | 140.549 | | 1.10 | T/T | CT | GG | CC | CC |
| 59 | 26 | FALSE | TRUE | Cardiovascular | | | | | | | | | | | | 110.544 | 172.133 | | | T/T | CC | CC | TT | CC |
| 71 | 29 | FALSE | FALSE | Cardiovascular | | | | | | | | | | | | 108.965 | 167.395 | | | T/T | CC | CC | CC | CT |
| 75 | 27 | FALSE | FALSE | Depression Anixiety | | | | | | | | | | | | 100.279 | 140.549 | | | T/T | CC | CG | TC | CC |
| 47 | 26 | FALSE | TRUE | None | | | | | | | | | | | | 145.286 | 161.868 | | | T/T | CC | CG | TC | CC |
| 68 | 28 | FALSE | TRUE | None | | | | | | | | | | | | 97.9104 | 154.762 | | | T/T | CT | CC | CC | CT |
| 77 | 22 | FALSE | TRUE | Cardiovascular | | | | | | | | | | | | 82.908 | 156.341 | | | T/T | CT | | CC | CC |
| 53 | 25 | TRUE | TRUE | Cardiovascular | | | | | | | | | | | | 112.123 | 164.237 | | 0.30 | T/T | CC | CC | TC | CC |
| 63 | 27 | TRUE | TRUE | Cardiovascular | | | | | | | | | | | | 123.178 | 167.395 | | | T/T | CT | CG | TC | CC |
| 49 | 26 | FALSE | TRUE | None | | | | | | | | | | | | 124.757 | 135.022 | 0.8 | 1.00 | T/T | CT | CC | TT | CC |
| 56 | 26 | FALSE | FALSE | None | 1785.58 | 1.256329 | 33.843 | 3.007385 | 22.42651 | 73.0096 | 34.8172 | 22.0903 | 43.0925 | 17.6098 | 54.0392 | 141.338 | 198.979 | 0.3 | 0.50 | T/T | CT | CC | CC | CC |
| 54 | 28 | FALSE | FALSE | other | 290.913 | | 10.0218 | | 3.0388 | 48.5563 | | | | 13.0869 | | 102.648 | 174.502 | | | T/T | CC | CC | TT | CC |
| 53 | 30 | FALSE | TRUE | None | 625.919 | 0.6133881 | 12.202 | 1.22633 | 3.861858 | 73.236 | 33.3423 | 19.8068 | 46.8509 | 12.222 | 43.258 | 101.069 | 177.66 | 3.2 | 4.20 | | CT | | TT | CT |
| 77 | 31 | TRUE | TRUE | Depression Anixiety | | | | | | | | | | | | 63.168 | | | | T/T | CT | GG | CC | CC |
| 74 | 31 | TRUE | TRUE | Depression Anixiety | | | | | | | | | | | | 114.492 | 112.913 | | | T/T | CC | CG | TC | CT |
| 71 | 27 | TRUE | TRUE | inflammatory | | | | | | | | | | | | 82.908 | 120.019 | | 8.00 | T/T | CC | CG | TC | CC |
| 72 | 24 | TRUE | TRUE | other | | | | | | | | | | | | 117.65 | 162.658 | | | T/T | CT | CC | TC | CT |
| 69 | 26 | TRUE | FALSE | Cardiovascular | 538.857 | 0.5720749 | 13.137 | 1.377895 | 18.9598 | 34.5439 | 35.2427 | 28.4578 | 36.2995 | 13.2273 | 44.0839 | 126.336 | 150.024 | 8.8 | 0.40 | T/T | CC | CG | CC | CC |
| 57 | 29 | FALSE | TRUE | Cardiovascular | | | | | | | | | | | | 147.655 | 177.66 | | 4.10 | T/T | CT | CC | TC | CT |
| 68 | 31 | TRUE | TRUE | Cardiovascular | | | | | | | | | | | | 104.227 | 152.393 | | 1.60 | | CT | CG | TC | CT |
| 56 | 24 | FALSE | TRUE | Cardiovascular | | | | | | | | | | | | 108.175 | 177.66 | | | | CC | CG | TC | CC |
| 51 | 27 | FALSE | TRUE | None | 904.674 | | 17.0991 | | 5.136149 | 62.5991 | | | | 17.8814 | | 93.1728 | 148.445 | | | | CC | CC | TC | CC |
| 57 | 27 | TRUE | TRUE | None | 391.134 | 0.5716286 | 11.0482 | 1.193169 | 21.16387 | 128.436 | 40.417 | 16.0022 | 43.5808 | 11.9289 | 41.2832 | 151.603 | 158.71 | 0.4 | 0.40 | T/T | CT | CC | TC | CT |
| 67 | 30 | TRUE | TRUE | Cardiovascular | | | | | | | | | | | | 103.438 | 183.187 | | | T/T | CT | CG | TC | CC |
| 67 | 30 | TRUE | FALSE | inflammatory | | | | | | | | | | | | 116.071 | 158.71 | | | T/T | CC | CC | TT | CC |
| 42 | 27 | TRUE | FALSE | other | | | | | | | | | | | | 102.648 | 168.974 | | 2.60 | T/T | CC | CC | CC | CC |
| 52 | 30 | FALSE | TRUE | None | 537.792 | 1.568031 | 20.9466 | 3.084976 | 8.179232 | 122.266 | 41.1095 | 17.695 | 41.1955 | 20.3993 | 37.0234 | 142.918 | 187.925 | 0.7 | 1.00 | T/T | CT | CG | TC | CC |
| 57 | 36 | TRUE | TRUE | None | | | | | | | | | | | | 107.386 | 102.648 | | 0.70 | T/T | CT | CC | CC | CC |
| 57 | 29 | TRUE | FALSE | Urology | | | | | | | | | | | | 105.017 | 132.653 | | | | CC | CG | CC | CC |
| 57 | 34 | FALSE | TRUE | Cardiovascular | 255.97 | 1.09502 | 13.1997 | 2.884846 | 3.387396 | 52.5021 | 38.9058 | 18.0254 | 43.0689 | 14.0818 | 42.2872 | 134.232 | 123.178 | 0.9 | 0.90 | T/T | CC | | TC | CC |
| 69 | 25 | FALSE | TRUE | None | 507.104 | 0.753212 | 17.7262 | 1.698067 | 32.66679 | 108.759 | 39.1653 | 21.3945 | 39.4403 | 16.3807 | 39.4885 | 170.554 | 159.499 | 1 | 1.20 | T/T | CT | GG | TC | CC |
| 72 | 30 | TRUE | TRUE | Cardiovascular | | | | | | | | | | | | 116.071 | 138.97 | | 3.30 | C/T | CC | CG | TC | CC |
| 69 | 30 | FALSE | TRUE | Urology | | | | | | | | | | | | 112.123 | 151.603 | | | T/T | CC | CG | CC | CC |
| 65 | 28 | TRUE | TRUE | None | | | | | | | | | | | | 112.913 | 146.866 | 2 | | T/T | CC | CC | TT | CC |
| 68 | 26 | TRUE | TRUE | Cardiovascular | | | | | | | | | | | | 114.492 | 172.133 | | 0.80 | T/T | CC | CG | TC | CC |
| 64 | 29 | FALSE | TRUE | None | | | | | | | | | | | | 86.856 | | | | T/T | CT | | TT | CT |
| 66 | 31 | FALSE | TRUE | Urology | | | | | | | | | | | | 94.752 | 144.497 | | | T/T | CC | CG | TC | CT |
| 34 | 25 | TRUE | TRUE | None | 369.234 | | 13.6896 | | 4.849647 | 92.5479 | | | | 17.7942 | | 123.178 | 198.979 | | 0.30 | T/T | CC | CG | TT | CT |
| 77 | 26 | TRUE | TRUE | None | | | | | | | | | | | | 195.031 | 226.615 | | | T/T | CT | CC | CC | CT |
| 59 | 25 | FALSE | TRUE | None | 386.944 | 1.340778 | 14.5238 | 2.384346 | 87.74142 | 67.8459 | 38.4876 | 13.8915 | 47.6209 | 15.064 | 29.8732 | 101.069 | 157.92 | 0.6 | 0.50 | T/T | CC | CG | TC | CT |
| 68 | 29 | TRUE | TRUE | Cardiovascular | | | | | | | | | | | | 124.757 | 174.502 | | | C/T | CT | GG | CC | CC |
| 81 | 22 | FALSE | FALSE | None | | | | | | | | | | | | 123.967 | 154.762 | | 1.60 | T/T | CC | CG | CC | CT |
| 59 | | TRUE | TRUE | None | 509.946 | | 10.8273 | | 1.927 | 53.978 | | | | 14.1514 | | 105.017 | 149.234 | 134 | | C/T | CC | CC | TC | CC |
| 74 | 25 | FALSE | TRUE | Cardiovascular | | | | | | | | | | | | 124.757 | 198.19 | 2.2 | | T/T | CC | | TC | TT |
| 67 | 31 | TRUE | TRUE | Cardiovascular | | | | | | | | | | | | 108.965 | 153.972 | | | T/T | CC | CG | CC | CC |
| 60 | 29 | TRUE | TRUE | None | | | | | | | | | | | | 142.918 | 150.024 | | | T/T | CT | CG | TC | CC |
| 66 | 41 | FALSE | TRUE | Cardiovascular | | | | | | | | | | | | 118.44 | 144.497 | | 0.50 | T/T | CC | CG | TC | CT |
| 68 | 25 | FALSE | FALSE | None | 519.896 | 0.3957939 | 12.1227 | 1.795731 | 3.54807 | 77.2799 | 39.2566 | 29.4725 | 31.2709 | 20.0194 | 42.5108 | 123.967 | 143.707 | 5.1 | 3.80 | T/T | CC | CG | TT | CC |
| 50 | 26 | FALSE | TRUE | None | 215.237 | | 11.8463 | | 3.453107 | 35.8729 | | | | 15.1608 | | 119.23 | 141.338 | | | T/T | CC | CG | TC | CC |
| 59 | 31 | FALSE | TRUE | None | | | | | | | | | | | | 116.861 | 133.442 | | | T/T | CC | CC | TC | CT |
| 42 | 24 | FALSE | TRUE | None | | | | | | | | | | | | 121.598 | 159.499 | 0.4 | 0.60 | T/T | | CC | TC | CC |
| 63 | 28 | FALSE | TRUE | None | 253.258 | 0.6674004 | 17.681 | 1.753353 | 5.576035 | 67.1539 | 36.4676 | 15.0881 | 48.4443 | 18.1037 | 42.8029 | 102.648 | 175.291 | 1.3 | 1.60 | T/T | CC | GG | TT | CT |
| 51 | 24 | FALSE | TRUE | None | 954.041 | 0.6332703 | 17.6342 | 1.499928 | 3.443162 | 67.3658 | 34.4047 | 27.1643 | 38.4311 | 14.9792 | 51.1277 | 127.915 | 174.502 | 0.6 | 0.50 | T/T | CC | CC | TC | CC |
| 62 | 27 | FALSE | TRUE | Cardiovascular | 252.917 | | 10.836 | | 4.028494 | 61.4674 | | | | 19.6483 | | 108.175 | 165.026 | | 4.10 | | | CG | | CC |
| 74 | 25 | TRUE | TRUE | inflammatory | 567.77 | 0.3108559 | 10.7089 | 1.591164 | 3.24687 | 140.843 | 35.0683 | 14.345 | 50.5867 | 18.3185 | 45.1216 | 104.227 | 145.286 | 2.7 | 2.30 | T/T | CT | CG | TT | CC |
| 68 | 29 | TRUE | TRUE | Diabetes | 447.672 | 1.424477 | 31.3349 | 2.686256 | 10.75963 | 111.105 | 45.2376 | 12.7351 | 42.0273 | 24.5356 | 24.9793 | 131.074 | 138.18 | 1.3 | 1.00 | T/T | CC | CG | TT | CC |
| 52 | 28 | FALSE | TRUE | None | 251.997 | 0.3730698 | 8.22737 | 2.148445 | 4.08725 | 107.991 | 43.8105 | 16.8767 | 39.3129 | 18.0441 | 31.3465 | 111.334 | 107.386 | 0.5 | 0.50 | T/T | CT | GG | CC | CC |
| 54 | 26 | FALSE | TRUE | None | 245.987 | 1.491623 | 19.7252 | 1.538052 | 6.262001 | 52.6315 | 41.8017 | 15.6841 | 42.5142 | 17.5662 | 28.2826 | 123.178 | 155.551 | 1.7 | 2.00 | T/T | CC | CG | TT | CT |
| 63 | 25 | FALSE | TRUE | Cardiovascular | | | | | | | | | | | | 101.858 | 145.286 | | | T/T | CC | CC | TT | CC |
| 62 | 25 | TRUE | TRUE | inflammatory | | | | | | | | | | | | 161.078 | 227.405 | | | | CC | CC | TC | CT |
| 53 | 23 | FALSE | TRUE | None | 257.106 | 1.060638 | 11.9518 | 1.72752 | 3.168756 | 45.987 | 40.5969 | 15.5505 | 43.8526 | 16.5918 | 36.6144 | 138.97 | 202.927 | 1.2 | 1.50 | T/T | TT | CG | CC | CC |
| 57 | 26 | FALSE | TRUE | None | | | | | | | | | | | | 123.178 | 198.19 | | | T/T | CC | CG | TC | CT |
| 51 | 25 | FALSE | TRUE | None | | | | | | | | | | | | 131.074 | 180.818 | | 1.30 | T/T | CC | CC | CC | CC |
| 45 | 29 | FALSE | FALSE | other | | | | | | | | | | | | 131.074 | 171.343 | 0.8 | | T/T | | | TT | CC |
| 58 | 24 | FALSE | TRUE | None | | | | | | | | | | | | 110.544 | 155.551 | | | T/T | CT | CG | TC | CT |
| 73 | 25 | FALSE | TRUE | Cardiovascular | | | | | | | | | | | | 92.3832 | 149.234 | | | T/T | CC | CC | TC | CT |
| 78 | 27 | TRUE | TRUE | Cardiovascular | | | | | | | | | | | | 87.6456 | 168.974 | 3.4 | | T/T | CC | GG | CC | CT |

| | | | | | | | | | | | | | | | | | | | | | | | | |
|---|---|---|---|---|---|---|---|---|---|---|---|---|---|---|---|---|---|---|---|---|---|---|---|---|
| 61 | 26 | FALSE | TRUE | None | | | | | | | | | | | | 114.492 | 177.66 | | | T/T | CC | CC | TC | CT |
| 75 | 31 | TRUE | TRUE | Cardiovascular | 276.412 | 0.9618874 | 13.5235 | 1.791771 | 4.992732 | 77.2404 | 38.8017 | 14.588 | 46.6103 | 18.5881 | 46.4729 | 147.655 | 146.076 | 2.3 | 2.50 | T/T | CC | GG | TT | CC |
| 48 | 27 | FALSE | TRUE | None | 218.416 | | 7.63555 | | 3.397511 | 72.5129 | | | | 11.116 | | 120.019 | 127.915 | 2.6 | | T/T | CC | CC | TT | CT |
| 72 | 27 | FALSE | TRUE | Cardiovascular | 521.598 | 0.6430817 | 11.5531 | 1.960712 | 4.252124 | 47.3729 | 47.1508 | 13.761 | 39.0883 | 22.16 | 38.9052 | 135.022 | 123.178 | 1.6 | 1.60 | T/T | CC | GG | TC | CC |
| 72 | 29 | FALSE | TRUE | Cardiovascular | | | | | | | | | | | | 112.123 | | 3.1 | | T/T | CC | CG | TT | CC |
| 46 | 29 | FALSE | TRUE | None | | | | | | | | | | | | 147.655 | 123.178 | 0.9 | 1.40 | | | | | CT |
| 37 | 33 | FALSE | TRUE | None | | | | | | | | | | | | 116.861 | 112.123 | | 0.50 | T/T | CT | CC | TC | CC |
| 49 | 36 | TRUE | TRUE | None | | | | | | | | | | | | 123.967 | 116.071 | | 1.60 | C/T | CT | GG | CC | CC |
| 30 | 28 | FALSE | FALSE | None | 1006.74 | | 23.5017 | | 8.288718 | 40.66 | | | | 13.5221 | | 82.908 | 138.97 | | | T/T | CC | CG | TC | CC |
| 65 | 28 | FALSE | TRUE | None | 448.266 | 0.5283127 | 14.4594 | 1.854195 | 66.88755 | 66.6933 | 36.3183 | 17.5917 | 46.09 | 15.543 | 43.0237 | 119.23 | 127.915 | 4.6 | 6.90 | T/T | CC | | TT | CC |
| 48 | 22 | FALSE | FALSE | None | | | | | | | | | | | | 150.814 | 168.185 | 0.9 | 1.00 | T/T | CC | CC | TT | CT |
| 66 | 24 | TRUE | TRUE | None | | | | | | | | | | | | 142.918 | 140.549 | 1.9 | 2.20 | T/T | CC | CG | TC | CT |
| 29 | 25 | TRUE | TRUE | None | | | | | | | | | | | | 122.388 | | 1.5 | | T/T | CT | CG | CC | CT |
| 54 | 26 | FALSE | TRUE | None | | | | | | | | | | | | 123.178 | 146.866 | 1.1 | 1.00 | T/T | CC | CG | CC | CC |
| 47 | 29 | TRUE | TRUE | None | | | | | | | | | | | | 123.178 | | 0.5 | | T/T | CT | CC | TT | CC |
| 72 | 29 | FALSE | TRUE | Cardiovascular | | | | | | | | | | | | 222.667 | | 1.6 | | T/T | TT | CG | TC | CC |
| 49 | 27 | TRUE | TRUE | None | | | | | | | | | | | | 181.608 | 176.87 | 0.3 | 0.30 | T/T | CC | CG | TC | CT |
| 47 | 19 | FALSE | TRUE | None | | | | | | | | | | | | 155.551 | 201.348 | 1.2 | 1.80 | T/T | CC | CG | TC | TT |
| 34 | 29 | TRUE | TRUE | None | | | | | | | | | | | | 131.074 | 161.868 | 0.9 | 1.00 | T/T | CT | CC | TT | CC |
| 60 | 24 | TRUE | TRUE | None | | | | | | | | | | | | 137.39 | 149.234 | 3.4 | 3.90 | T/T | CT | CG | TC | CC |
| 62 | 25 | TRUE | FALSE | Cardiovascular | | | | | | | | | | | | 131.074 | 127.126 | 3.2 | 2.70 | T/T | CC | CG | TC | TT |
| 67 | 32 | FALSE | TRUE | Cardiovascular | | | | | | | | | | | | 304.786 | | 2.2 | | T/T | CT | CG | TC | CC |
| 56 | 30 | FALSE | TRUE | None | | | | | | | | | | | | 155.551 | 148.445 | 1.9 | 1.60 | T/T | CC | CG | TT | CC |
| 69 | 29 | FALSE | TRUE | Cardiovascular | | | | | | | | | | | | 106.596 | 160.289 | 0.7 | 0.70 | T/T | CT | CG | TT | CC |
| 76 | 30 | FALSE | TRUE | Urology | 453.662 | 1.336559 | 20.7542 | 1.812645 | 41.18567 | 45.6111 | 33.97 | 19.978 | 46.052 | 16.5183 | 41.0409 | 127.915 | 180.029 | 4 | 4.20 | T/T | CC | CC | TC | CC |
| 54 | 33 | TRUE | TRUE | Cardiovascular | | | | | | | | | | | | 105.806 | 162.658 | | | T/T | CT | CG | TT | CT |
| 68 | 31 | FALSE | FALSE | None | | | | | | | | | | | | 123.967 | 177.66 | 3.8 | 3.60 | C/T | CC | GG | CC | CC |
| 77 | 30 | FALSE | TRUE | other | 365.548 | 1.217846 | 15.047 | 1.471645 | 5.52115 | 50.3233 | 37.2145 | 10.4959 | 52.2897 | 15.4001 | 51.8371 | 141.338 | 165.026 | 6.8 | 5.10 | | CT | CC | TT | CT |
| 77 | 28 | TRUE | FALSE | None | | | | | | | | | | | | 105.806 | 147.655 | 2 | 2.00 | T/T | CT | GG | TC | CT |
| 71 | 34 | FALSE | TRUE | Urology | 171.862 | 1.242275 | 10.2117 | 1.173393 | 3.92083 | 32.1986 | 37.1446 | 10.7597 | 52.0957 | 19.0561 | 41.6884 | 122.388 | 162.658 | 1.9 | 3.30 | T/T | CC | CC | TC | CC |
| 54 | 27 | FALSE | TRUE | inflammatory | 251.664 | 0.4638138 | 10.2199 | 1.737995 | 4.89065 | 46.2018 | 42.4895 | 17.9041 | 39.6064 | 17.0956 | 46.8719 | 149.234 | 167.395 | 0.6 | 0.40 | T/T | CC | | CC | CT |
| 69 | 33 | FALSE | TRUE | None | 182.524 | 0.6238632 | 12.0582 | 1.112942 | 3.953263 | 42.9072 | 39.1813 | 23.0809 | 37.7379 | 14.9802 | 35.8823 | 153.182 | 131.863 | 3.8 | 3.70 | C/T | CC | CC | TC | CT |
| 45 | 28 | FALSE | TRUE | None | 307.349 | 0.937851 | 11.7872 | 1.832672 | 3.387524 | 61.762 | 39.8428 | 13.1276 | 47.0295 | 14.2462 | 44.1683 | 127.126 | 150.024 | 0.6 | 0.60 | T/T | CC | CC | TT | CC |
| 59 | 26 | FALSE | TRUE | None | | | | | | | | | | | | 147.655 | 172.133 | 1.7 | 1.70 | T/T | CT | CG | TC | CT |
| 60 | 29 | FALSE | TRUE | Cardiovascular | 166.013 | 0.590332 | 9.28207 | 0.967781 | 1.581464 | 38.6442 | 37.7587 | 9.09319 | 53.1481 | 13.2827 | 31.671 | 154.762 | 149.234 | 1 | 1.80 | T/T | CC | CC | CC | CT |
| 70 | 31 | TRUE | TRUE | Cardiovascular | 149.001 | 1.044029 | 10.4667 | 1.170303 | 6.405822 | 40.2017 | 40.4335 | 18.1328 | 41.4338 | 18.2369 | 49.5648 | 132.653 | 138.97 | 2 | 2.10 | | CT | CG | TT | CC |
| 76 | 37 | TRUE | FALSE | Cardiovascular | | | | | | | | | | | | 139.759 | | 3.9 | | T/T | CT | CC | CC | CC |
| 63 | 28 | FALSE | TRUE | Depression Anixiety | | | | | | | | | | | | 156.341 | 146.866 | 0.6 | 0.70 | T/T | CT | CC | TC | CC |
| 52 | 24 | FALSE | TRUE | None | 390.301 | 0.9521637 | 14.1961 | 1.926338 | 3.967505 | 62.8269 | 39.8278 | 13.0113 | 47.1609 | 11.1606 | 41.7102 | 97.1208 | 146.866 | 0.6 | 0.70 | T/T | CT | CG | TC | CC |
| 59 | 27 | TRUE | TRUE | Depression Anixiety | | | | | | | | | | | | 135.811 | 163.447 | | 1.00 | T/T | CC | CG | TT | CC |
| 44 | 29 | FALSE | TRUE | None | 205.986 | 0.9210434 | 8.9998 | 0.828098 | 2.915546 | 72.2794 | 41.7862 | 20.2721 | 37.9416 | 28.2412 | 41.2256 | 117.65 | 139.759 | 0.3 | 0.30 | T/T | CT | CC | TT | CC |
| 46 | 23 | FALSE | TRUE | other | 216.168 | 1.170387 | 12.5329 | 1.616707 | 4.664037 | 67.9466 | 42.081 | 15.3755 | 42.5436 | 13.8778 | 42.3809 | 142.128 | 207.665 | 0.6 | 0.70 | T/T | CT | CC | TC | CC |
| 61 | 26 | FALSE | TRUE | Cardiovascular | 240.703 | 1.169815 | 9.82373 | 1.141376 | 19.07937 | 39.0224 | 37.0933 | 16.7188 | 46.1879 | 13.1791 | 45.3478 | 129.494 | 157.13 | 0.7 | 1.10 | T/T | TT | CG | TC | CC |
| 47 | 30 | TRUE | TRUE | None | | | | | | | | | | | | 154.762 | 128.705 | 1.1 | 0.90 | T/T | CC | CG | CC | CC |
| 56 | 27 | FALSE | TRUE | other | | | | | | | | | | | | 142.128 | 173.712 | 1.7 | 2.90 | | CT | CC | TT | CC |
| 66 | 21 | TRUE | TRUE | Cardiovascular | 293.873 | 0.9424515 | 14.1331 | 1.575211 | 4.884973 | 32.297 | 49.0112 | 16.3579 | 34.6309 | 17.3008 | 49.6001 | 120.019 | 153.972 | 1.5 | 2.40 | T/T | CC | GG | TC | CC |
| 43 | 28 | FALSE | TRUE | None | 223.555 | 0.9878998 | 14.6375 | 1.215294 | 7.168181 | 70.0666 | 33.8077 | 7.39118 | 58.8012 | 19.3051 | 42.7207 | 148.445 | 146.076 | 0.4 | 0.70 | T/T | CC | CC | TC | CC |
| 57 | 33 | FALSE | TRUE | Cardiovascular | | | | | | | | | | | | 127.126 | | 15.7 | | T/T | CC | CG | CC | CT |
| 59 | 28 | TRUE | TRUE | None | 588.303 | 0.6839561 | 12.4275 | 1.53096 | 3.999352 | 88.3813 | 39.5333 | 19.1313 | 41.3354 | 14.7335 | 41.6743 | 101.858 | 148.445 | 0.6 | 1.40 | T/T | CT | CG | TC | CT |
| 70 | 25 | FALSE | TRUE | Cardiovascular | | | | | | | | | | | | 131.863 | 147.655 | 2.5 | 3.40 | T/T | CC | CG | TC | CT |
| 51 | 28 | FALSE | TRUE | None | | | | | | | | | | | | 112.913 | 146.076 | 0.5 | 0.80 | T/T | CT | CG | TC | CC |
| 47 | 31 | FALSE | TRUE | None | 549.054 | 0.5935898 | 27.1867 | 1.244846 | 29.84706 | 55.3574 | 39.0527 | 20.3329 | 40.6144 | 18.3192 | 48.038 | 135.811 | 156.341 | 0.5 | 1.00 | | CC | CG | CC | CC |
| 58 | 30 | TRUE | TRUE | None | 343.415 | 1.049274 | 15.2613 | 1.57029 | 10.37264 | 62.8229 | 38.8441 | 18.2677 | 42.8882 | 25.868 | 27.6857 | 124.757 | 142.918 | 0.4 | 0.50 | C/T | CC | CC | CC | CT |
| 27 | 23 | FALSE | TRUE | None | 359.623 | 0.5733337 | 13.196 | 1.810692 | 3.942687 | 48.4652 | 40.0656 | 19.0815 | 40.8529 | 15.0199 | 49.1509 | 123.178 | 172.133 | 0.9 | 1.30 | T/T | CC | GG | CC | |
| 33 | 30 | FALSE | TRUE | None | | | | | | | | | | | | 123.178 | 136.601 | 0.3 | 0.40 | T/T | CT | CG | TT | CT |
| 80 | 28 | TRUE | FALSE | Urology | | | | | | | | | | | | 172.922 | 168.185 | 2.5 | 3.30 | C/T | CC | GG | TT | CT |
| 38 | 25 | FALSE | TRUE | None | 462.278 | 1.310707 | 21.1443 | 2.133858 | 6.093896 | 73.0901 | 64.8953 | 4.56247 | 30.5422 | 10.468 | 27.1672 | 138.97 | 166.606 | 0.6 | 0.50 | C/T | CC | CG | TT | CT |
| 39 | 28 | FALSE | TRUE | Cardiovascular | | | | | | | | | | | | 149.234 | | 0.6 | | T/T | TT | GG | TC | CC |
| 45 | 28 | FALSE | TRUE | None | 194.396 | 0.4035301 | 9.59035 | 0.645184 | 2.070306 | 126.851 | 38.6694 | 16.4796 | 44.851 | 13.8611 | 41.0661 | 151.603 | 171.343 | 0.8 | 0.80 | T/T | CC | CG | TC | CT |
| 40 | 22 | TRUE | TRUE | None | 417.667 | 1.022835 | 16.1628 | 2.008869 | 5.833 | 57.0451 | 41.664 | 13.6153 | 44.7207 | 20.4012 | 32.5679 | 146.866 | 183.977 | 1.7 | 1.60 | | CC | CG | TT | CC |
| 68 | 29 | TRUE | TRUE | Urology | | | | | | | | | | | | 157.92 | 181.608 | 3.3 | 4.40 | T/T | CT | CG | TC | CC |
| 59 | 27 | FALSE | TRUE | None | | | | | | | | | | | | 128.705 | 141.338 | 0.4 | 0.60 | C/T | CC | CC | TT | CC |
| 47 | 29 | TRUE | TRUE | None | | | | | | | | | | | | 147.655 | 123.967 | 0.6 | 0.70 | T/T | CT | CC | TT | CC |
| 57 | 24 | FALSE | TRUE | None | 263.154 | 0.7400131 | 11.0122 | 1.176525 | 8.738845 | 47.6921 | 31.4839 | 28.102 | 40.4141 | 14.178 | 48.4656 | 138.18 | 156.341 | 0.6 | 0.60 | C/T | CT | CC | TC | CT |
| 34 | 26 | FALSE | TRUE | None | 455.06 | 0.9463997 | 25.7017 | 2.5788 | 6.35593 | 63.8508 | 41.2411 | 10.5653 | 48.1936 | 18.7885 | 39.5081 | 126.336 | 140.549 | 1.5 | 1.40 | T/T | CC | CC | TT | CC |
| 66 | 34 | TRUE | TRUE | Cardiovascular | 216.958 | 0.6721039 | 23.316 | 0.370663 | 3.863506 | 30.028 | 39.4645 | 13.9878 | 46.5477 | 17.2873 | 39.5335 | 97.9104 | 179.239 | 3.7 | 4.90 | T/T | CC | CG | TC | CC |
| 65 | 27 | FALSE | TRUE | other | 619.224 | 0.7155075 | 24.9134 | 2.030788 | 36.2296 | 127.653 | 41.0144 | 14.4332 | 44.5524 | 16.3396 | 36.4428 | 127.126 | 168.185 | 1.8 | 1.50 | T/T | CT | CC | TT | CT |
| 68 | 27 | TRUE | TRUE | None | 164.762 | 0.7824364 | 8.74741 | 1.517025 | 3.528651 | 96.1905 | 35.8603 | 13.5016 | 50.6381 | 12.5537 | 48.4312 | 127.915 | 142.918 | 3.4 | 3.70 | T/T | CT | CG | TT | CC |
| 50 | 25 | FALSE | TRUE | None | | | | | | | | | | | | 142.918 | 168.974 | 0.8 | 1.30 | T/T | CT | CG | TT | CC |
| 36 | 26 | FALSE | TRUE | None | 396.293 | | 18.8189 | | 3.153554 | 218.978 | | | | 15.5728 | | 137.39 | | 0.4 | | C/T | CC | GG | TC | CT |
| 70 | 29 | FALSE | TRUE | None | 331.065 | 0.6805763 | 14.4262 | 2.564041 | 7.313337 | 87.6144 | 32.3297 | 14.3779 | 53.2924 | 17.1694 | 38.6505 | 128.705 | 114.492 | 1.3 | 1.80 | T/T | CT | GG | CC | CT |
| 49 | 29 | FALSE | TRUE | None | 436.09 | 0.4366837 | 8.95932 | 0.675453 | 52.17785 | 37.4373 | 36.5978 | 20.4486 | 42.9536 | 15.1878 | 44.919 | 117.65 | 127.915 | 1.9 | 2.00 | T/T | CC | CC | CC | CT |
| 48 | 26 | FALSE | FALSE | Depression Anix | 600.463 | 0.8824272 | 19.2302 | 2.00354 | 80.97929 | 49.7721 | 38.2668 | 15.6185 | 46.1147 | 16.1292 | 48.1918 | 145.286 | 131.863 | 0.6 | 1.00 | T/T | CT | GG | TC | CC |
| 42 | 24 | TRUE | TRUE | None | 239.537 | 0.8286171 | 11.7186 | 4.302315 | 3.393859 | 55.2208 | 34.4719 | 21.0462 | 44.4819 | 12.0179 | 52.9873 | 126.336 | 132.653 | 1.2 | 0.60 | T/T | CC | CG | CC | CT |
| 45 | 30 | FALSE | TRUE | inflammatory | 375.935 | 1.018711 | 17.1803 | 2.989552 | 4.710251 | 158.711 | 41.6399 | 23.4178 | 34.9423 | 23.5825 | 40.3221 | 119.23 | 115.282 | 0.3 | 0.30 | T/T | CT | GG | TT | CT |
| 45 | 27 | TRUE | TRUE | None | 338.803 | 0.8857193 | 11.472 | 6.597713 | 6.960601 | 74.2661 | 44.4537 | 18.0187 | 37.5276 | 12.2479 | 33.192 | 142.128 | 157.92 | 0.7 | 0.50 | | CT | CC | CC | CC |
| 38 | 23 | TRUE | TRUE | None | 322.621 | 0.8058739 | 10.9647 | 1.116951 | 3.759257 | 52.2632 | 46.9996 | 16.1412 | 36.8592 | 13.1646 | 40.2487 | 116.861 | 118.44 | 0.4 | 0.40 | T/T | CT | CC | TC | CC |
| 42 | 27 | FALSE | TRUE | None | 148.84 | 0.8808861 | 11.2583 | 0.852215 | 3.868558 | 134.173 | 37.7135 | 23.9028 | 38.3837 | 23.2743 | 39.224 | 127.126 | 154.762 | 0.7 | 0.90 | C/T | CC | CC | TC | CC |
| 62 | 21 | FALSE | TRUE | None | | | | | | | | | | | | 112.913 | | 1.4 | | T/T | CT | CG | TC | CT |
| 71 | 32 | FALSE | TRUE | Cardiovascular | 250.864 | 0.3030167 | 9.11707 | 0.984104 | 2.525462 | 83.5916 | 40.3418 | 17.8246 | 41.8336 | 10.5402 | 28.4043 | 127.126 | 122.388 | 3.3 | 3.50 | T/T | CT | CC | TT | CC |
| 47 | 28 | FALSE | TRUE | None | 283.621 | 0.9183159 | 28.6369 | 1.307892 | 3.217366 | 35.6852 | 38.3286 | 11.8855 | 49.7859 | 14.119 | 30.17 | 107.386 | 146.866 | 0.7 | 0.60 | T/T | CT | CG | TT | CT |
| 52 | 24 | FALSE | TRUE | None | | | | | | | | | | | | 141.338 | 165.816 | 0.6 | 0.30 | C/T | CC | CG | TC | CT |
| 62 | 23 | FALSE | TRUE | None | | | | | | | | | | | | 108.175 | 120.809 | 0.8 | 1.00 | T/T | TT | CG | TT | CT |
| 37 | 29 | FALSE | TRUE | inflammatory | | | | | | | | | | | | 105.806 | | 1.3 | | | CC | CG | TC | CC |
| 79 | 38 | FALSE | TRUE | Cardiovascular | 161.652 | 0.474762 | 9.36514 | 1.201073 | 7.031877 | 77.1986 | 39.4176 | 15.1009 | 45.4815 | 13.7225 | 40.3019 | 108.965 | 131.863 | 1.1 | 1.10 | T/T | CT | CG | TC | CC |
| 35 | 26 | FALSE | TRUE | None | | | | | | | | | | | | 102.648 | 142.128 | 1.2 | 1.40 | T/T | CC | CC | TT | CC |
| 35 | 29 | FALSE | TRUE | None | | | | | | | | | | | | 114.492 | 176.87 | 1.6 | 1.80 | T/T | CC | CC | TT | CT |
| 75 | 35 | FALSE | FALSE | Urology | 678.872 | | 10.3847 | | 3.159621 | 30.0777 | | | | 14.1042 | | 89.2248 | 150.024 | 5.3 | 5.70 | | CT | | CC | CC |
| 46 | 29 | FALSE | TRUE | None | 744.615 | 0.7860031 | 28.556 | 3.242687 | 59.8266 | 71.5207 | 46.077 | 18.0565 | 35.8665 | 11.9132 | 30.5387 | 151.603 | 178.45 | 0.6 | 0.60 | T/T | CC | CG | TC | CC |
| 29 | 29 | FALSE | TRUE | None | 315.979 | 0.7887268 | 19.7626 | 2.779762 | 7.49402 | 71.5361 | 36.1332 | 15.8126 | 48.0542 | 20.1736 | 41.5035 | 103.438 | 143.707 | 0.8 | 1.00 | T/T | CC | CG | TC | CC |
| 53 | 32 | FALSE | TRUE | Cardiovascular | 954.112 | 1.304302 | 18.9619 | 1.91861 | 6.059649 | 44.4307 | 40.7737 | 18.0907 | 41.1356 | 17.6979 | 39.3401 | 105.017 | 113.702 | 0.3 | 0.40 | T/T | CC | CG | TC | CC |
| 73 | 24 | FALSE | FALSE | Urology | 144.912 | | 10.1016 | | 5.053934 | 34.0334 | | | | 15.7295 | | 134.232 | 173.712 | 2.5 | 2.60 | | CC | | CC | CT |
| 57 | 34 | TRUE | TRUE | None | 409.406 | 0.6680832 | 11.0183 | 0.975052 | 4.159821 | 47.9796 | 44.4016 | 10.7472 | 44.8512 | 15.376 | 37.3145 | 120.019 | 146.076 | 2.3 | 2.80 | T/T | CT | CG | CC | CT |
| 73 | 29 | FALSE | TRUE | Cardiovascular | 330.992 | 1.375732 | 11.0741 | 1.649586 | 5.445708 | 71.7951 | 41.3529 | 22.3808 | 36.2663 | 14.2008 | 32.7107 | 106.596 | 114.492 | 2.8 | 3.80 | | CT | CG | TT | CC |
| 57 | 26 | FALSE | TRUE | None | 209.134 | 0.702301 | 11.6704 | 1.281834 | 4.576287 | 80.719 | 37.7637 | 11.9033 | 50.333 | 14.3784 | 41.0437 | 88.4352 | 148.445 | 1.1 | 1.40 | T/T | CC | CC | CC | CC |
| 63 | 30 | FALSE | TRUE | Cardiovascular | 209.121 | 0.3822441 | 9.81907 | 0.843079 | 2.289376 | 65.1848 | 37.4412 | 14.2955 | 48.2633 | 15.2539 | 46.5618 | 123.967 | 134.232 | 16.2 | 17.40 | T/T | CT | CG | TC | CC |
| 53 | 32 | FALSE | TRUE | Cardiovascular | | | | | | | | | | | | 130.284 | 114.492 | 0.5 | 1.00 | C/T | CT | CG | TC | CC |
| 78 | 22 | TRUE | TRUE | Cardiovascular | | | | | | | | | | | | 116.071 | 115.282 | 4.5 | 4.20 | | CC | | CC | CT |
| 34 | 31 | FALSE | TRUE | None | | | | | | | | | | | | 114.492 | 86.856 | 0.3 | 0.50 | | CC | CC | CC | CT |
| 58 | 24 | TRUE | FALSE | None | 414.508 | 0.1213493 | 19.8811 | 0.22551 | 25.50332 | 64.537 | 31.7055 | 22.1949 | 46.0996 | 12.0227 | 52.5549 | 128.705 | 160.289 | 1 | 0.50 | T/T | CC | CC | TC | CT |
| 62 | 25 | TRUE | TRUE | None | 307.558 | 0.9840431 | 10.3685 | 1.894951 | 5.479907 | 60.5907 | 35.6672 | 20.6306 | 43.7023 | 15.8779 | 37.6868 | 127.915 | 129.494 | 1.8 | 2.10 | T/T | CC | CC | CC | CC |
| 56 | 26 | TRUE | TRUE | Depression Anixiety | | | | | | | | | | | | 110.544 | | 1.1 | | T/T | CC | CC | CC | CT |
| 35 | 22 | TRUE | TRUE | None | | | | | | | | | | | | 86.0664 | 129.494 | 1.8 | 1.90 | | CT | CG | TC | CT |
| 48 | 26 | FALSE | TRUE | None | | | | | | | | | | | | 116.861 | 146.866 | | 3.70 | T/T | CC | CC | TT | CC |
| 58 | 31 | TRUE | TRUE | Depression Anixiety | | | | | | | | | | | | 117.65 | 123.967 | | 3.00 | T/T | CT | CC | TT | CC |
| 70 | 24 | FALSE | FALSE | other | 203.193 | 0.6703224 | 8.88823 | 1.840141 | 4.193832 | 74.2207 | 36.951 | 16.238 | 46.8111 | 12.8122 | 43.0942 | 116.861 | 129.494 | 0.9 | 2.60 | T/T | CC | CG | TC | CC |
